# Triple bonds of niobium with silicon, germaniun and tin: the tetrylidyne complexes [(κ^3^-tmps)(CO)_2_Nb≡E–R] (E = Si, Ge, Sn; tmps = MeSi(CH_2_PMe_2_)_3_; R = aryl)†
†Electronic supplementary information (ESI) available: Syntheses and analytical data of **1**, **2-Si**, **3-Ge**, **3-Sn** and **5-Ge**, illustrations of the IR and heteronuclear magnetic resonance spectra of **1**, **2-Si**, **3-Ge 3-Sn** and **5-Ge**, details of the cyclic voltammetric studies of **2-Si**, **3-Ge** and **3-Sn**, and crystal structure determination of **2-Si**, **3-Ge** (THF), **3-Sn** (toluene) and **5-Ge**. CCDC 1553387–1553389 and 1555671. For ESI and crystallographic data in CIF or other electronic format see DOI: 10.1039/c7sc02708g


**DOI:** 10.1039/c7sc02708g

**Published:** 2017-07-14

**Authors:** Alexander C. Filippou, David Hoffmann, Gregor Schnakenburg

**Affiliations:** a Institut für Anorganische Chemie, Rheinische Friedrich-Wilhelms-Universität Bonn, Gerhard-Domagk-Straße 1, 53121 Bonn, Germany. Email: filippou@uni-bonn.de

## Abstract

A systematic, efficient approach to first complexes containing a triple bond between niobium and the elements silicon, germanium or tin is reported. The approach involves a metathetical exchange of the niobium-centered nucleophile (NMe_4_)[Nb(CO)_4_(κ^2^-tmps)] (**1**) (tmps = MeSi(CH_2_PMe_2_)_3_) with a suitable organotetrel(ii)halide. Compound **1** was obtained from (NMe_4_)[Nb(CO)_6_] and the triphosphane tmps by photodecarbonylation. Reaction of **1** with the disilene *E*-Tbb(Br)Si=Si(Br)Tbb in the presence of 4-dimethylaminopyridine afforded selectively the red-brown silylidyne complex [(κ^3^-tmps)(CO)_2_Nb≡Si–Tbb] (**2-Si**, Tbb = 4-*tert*-butyl-2,6-bis(bis(trimethylsilyl)methyl)phenyl). Similarly, treatment of **1** with E(Ar^Mes^)Cl (E = Ge, Sn; Ar^Mes^ = 2,6-mesitylphenyl) afforded after elimination of (NMe_4_)Cl and two CO ligands the deep magenta colored germylidyne complex [(κ^3^-tmps)(CO)_2_Nb≡Ge–Ar^Mes^] (**3-Ge**), and the deep violet, light-sensitive stannylidyne complex [(κ^3^-tmps)(CO)_2_Nb≡Sn–Ar^Mes^] (**3-Sn**), respectively. Formation of **3-Sn** proceeds *via* the niobiastannylene [(κ^3^-tmps)(CO)_3_Nb–SnAr^Mes^] (**4-Sn**), which was detected by IR and NMR spectroscopy. The niobium tetrylidyne complexes **2-Si**, **3-Ge** and **3-Sn** were fully characterized and their solid-state structures determined by single-crystal X-ray diffraction studies. All complexes feature an almost linear tetrel coordination and the shortest Nb–E bond lengths (*d*(Nb–Si) = 232.7(2) pm; *d*(Nb–Ge) = 235.79(4) pm; *d*(Nb–Sn) = 253.3(1) pm) reported to date. Reaction of **3-Ge** with a large excess of H_2_O afforded upon cleavage of the Nb–Ge triple bond the hydridogermanediol Ge(Ar^Mes^)H(OH)_2_. Photodecarbonylation of [CpNb(CO)_4_] (Cp = η^5^-C_5_H_5_) in the presence of Ge(Ar^Mes^)Cl afforded the red-orange chlorogermylidene complex [Cp(CO)_3_Nb=Ge(Ar^Mes^)Cl] (**5-Ge**). The molecular structure of **5-Ge** features an upright conformation of the germylidene ligand, a trigonal–planar coordinated Ge atom, and a Nb–Ge double bond length of 251.78(6) pm, which lies in-between the Nb–Ge triple bond length of **3-Ge** (235.79(4) pm) and a Nb–Ge single bond length (267.3 pm). Cyclic voltammetric studies of **2-Si**, **3-Ge**, and **3-Sn** reveal several electron-transfer steps. One-electron oxidation and reduction of the germylidyne complex of **3-Ge** in THF are electrochemically reversible suggesting that both the radical cation and radical anion of **3-Ge** are accessible species in solution.

## Abstract 2

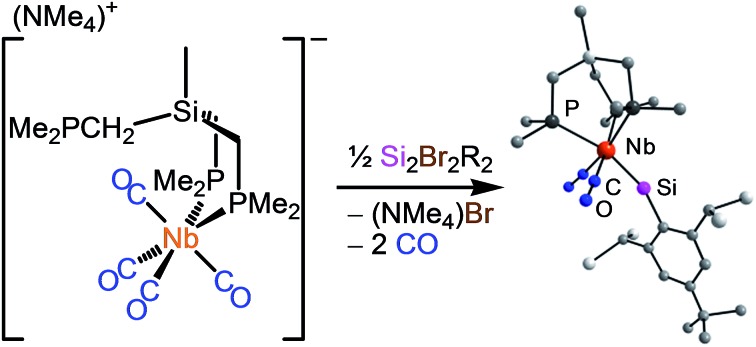
A systematic, efficient route to the first heavier tetrylidyne complexes of niobium [(κ^3^-tmps)(CO)_2_Nb≡E–R] (E = Si–Sn; R = aryl) starting from the carbonyl niobate (NMe_4_)[Nb(CO)_4_(κ^2^-tmps)] is presented.

## Introduction

Complexes of the general formula [L_*n*_M≡E–R] (M = d-block metal; E = Si–Pb; R = singly bonded group (*e.g.* alkyl, aryl); L_*n*_ = ligand sphere) featuring a triple bond between a d-block metal and the tetrels Si/Ge/Sn/Pb are an intriguing class of compounds with an auspicious synthetic potential originating from the highly reactive, polar M≡E bond.^1^–^4^ Isolation of these compounds is very challenging and requires specific stereoelectronic properties of the metal fragment L_*n*_M as well as a steric protection of the electrophilic tetrel center by a tailor-made, bulky substituent R to circumvent a head-to-tail cyclodimerisation or unintentional intra- or intermolecular σ-bond activations destroying the M≡E–R functionality. Whereas earlier work concentrated exclusively on group 6 metals, recent studies have shown that also group 7,2l,3d,4d group 8 (ref. 1*c* and 5) and even group 10 metals^6^ can be incorporated into triple bonding with the tetrels Si–Pb. Extension of this chemistry to the group 5 elements V–Ta seemed attractive to investigate whether the lower electronegativity and larger metallic radii of these elements compared to Cr–W would have an effect on the M≡E functionality. Group 5 metal complexes featuring a triple bond to the heavier tetrels (E = Si–Pb) are presently not known, and even compounds with a M=E double bond are very scarce and poorly characterized illustrating the challenge to make such compounds.^7^ We decided to address this issue, and present herein a systematic, efficient approach to the first complexes containing Nb≡E (E = Si–Sn) triple bonds.

## Results and discussion

**Scheme 1:**
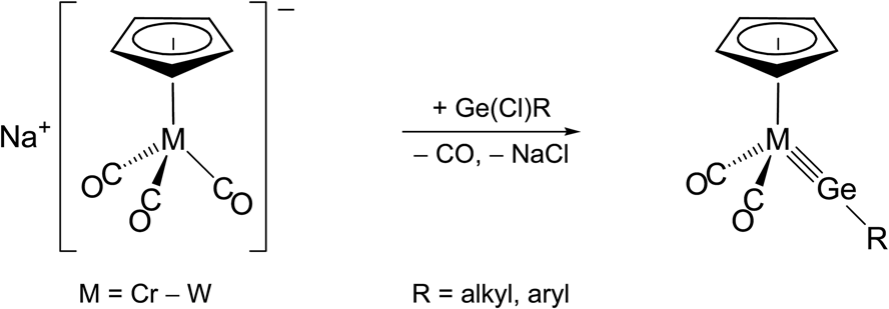
Preparation of half-sandwich group 6 metal germylidyne complexes by the salt elimination method.

Two methods have been employed so far for the formation of transition metal-tetrel (Si–Pb) triple bonds. The first method, abbreviated as the “salt elimination method”, involves a substitution reaction of a suitable anionic 18 VE metal complex with an organotetrel(ii) halide, as exemplified by the synthesis of Cp-substituted group 6 metal tetrylidyne complexes (Scheme 1).2a,2b,2i,2j,2m

**Scheme 2:**
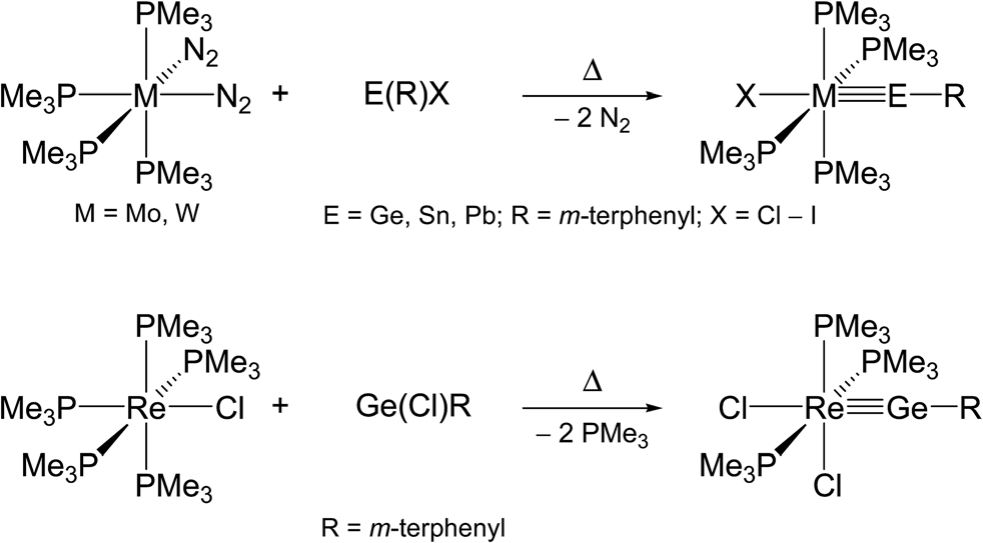
Preparation of neutral group 6 and 7 metal tetrylidyne complexes by the N_2_/PMe_3_ elimination method.

The second method, commonly termed “N_2_/PMe_3_ elimination method”, takes advantage of the exchange of labile ligands (mostly N_2_ or PMe_3_) in neutral 18 VE metal complexes by suitable organotetrel(ii) halides. This approach may afford directly neutral ylidyne complexes, as demonstrated by the syntheses of phosphane-substituted group 6 and 7 metal tetrylidyne complexes (Scheme 2).2f,2g,2l,3a,4a,4b

**Scheme 3:**
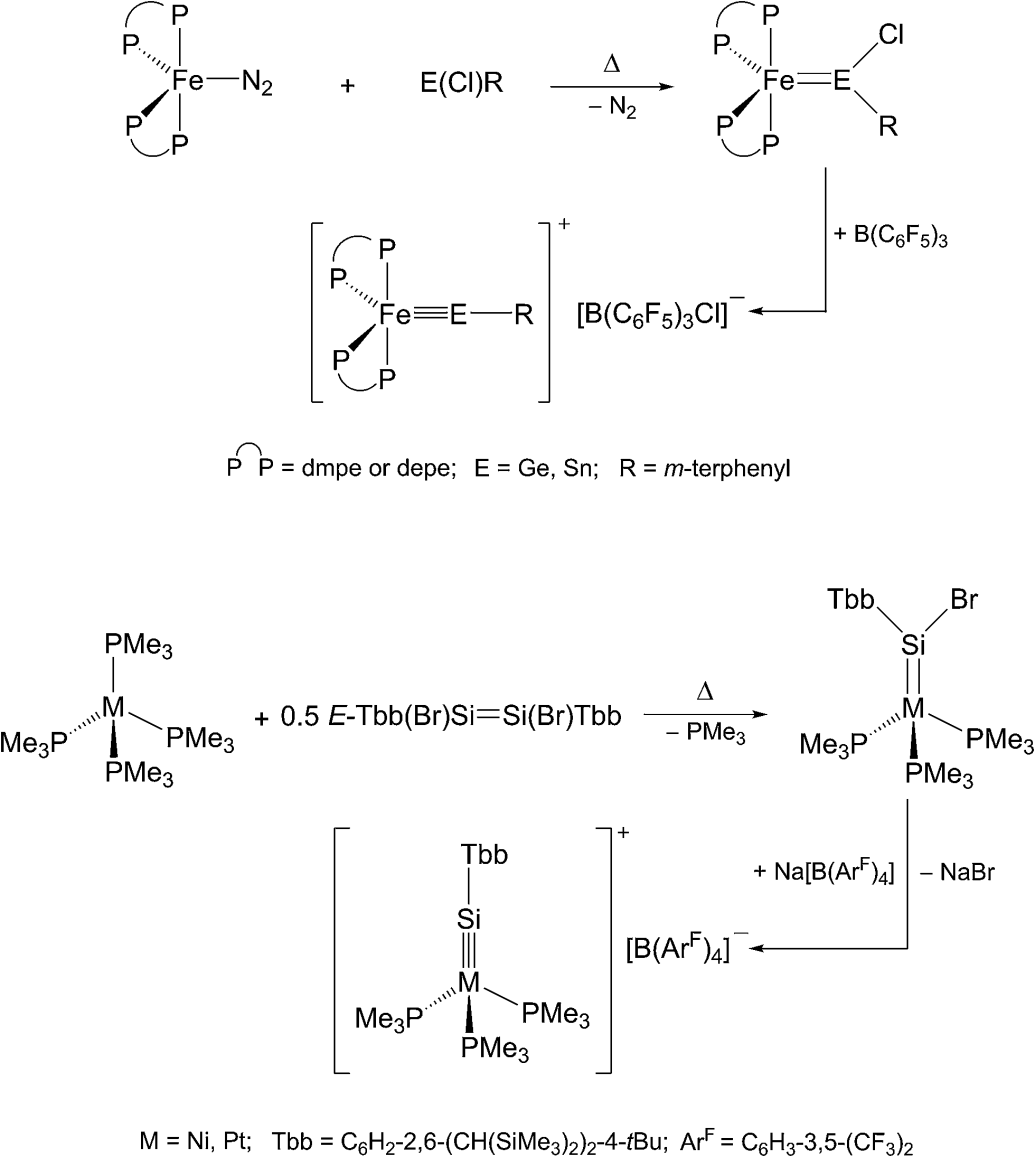
Preparation of group 8 and 10 metal tetrylidyne complexes *via* haloylidene complexes using the N_2_/PMe_3_ elimination method.

Alternatively, haloylidene complexes are initially obtained by this method, which are subsequently converted to cationic ylidyne complexes by halide abstraction. Examples demonstrating this reaction path include the preparation of group 8 and 10 ylidyne complexes (Scheme 3).^5^,^6^

We decided to apply the first method, given the availability of anionic niobium carbonyl complexes.^8^ At first, the homoleptic carbonyl niobate [Nb(CO)_6_]^–^ was chosen. For this purpose the canary yellow salts (NR_4_)[Nb(CO)_6_] (R = Me, Et) were prepared, following the method developed by J. E. Ellis *et al.*^9^ However, these compounds proved to be unreactive towards the *m*-terphenyltetrel(ii)halides E(Ar^Mes^)Cl (E = Ge, Sn; Ar^Mes^ = 2,6-mesitylphenyl; mesityl (Mes) = 2,4,6-trimethylphenyl).^10^ For example, IR monitoring of the reaction of (NEt_4_)[Nb(CO)_6_] with Ge(Ar^Mes^)Cl in refluxing toluene did not provide any evidence for a conversion of the niobate even after prolonged heating, probably due to the poor nucleophilicity of [Nb(CO)_6_]^–^. Therefore, as next we turned our attention to niobates containing ligands with a higher σ-donor/π-acceptor ratio than CO, such as trialkyl- or triarylphosphanes. Various carbonyl(phosphane) niobates of the general formula [Nb(CO)_4_L_2_]^–^ (L_2_ = bidentate di- or oligo-arylphosphane ligand) have been accessed from [Nb(CO)_6_]^–^ upon photolytic CO substitution.^11^ In order to increase the electron density at the metal centre, we decided to use the highly basic, albeit, very oxygen-sensitive, tripodal alkylphosphane MeSi(CH_2_PMe_2_)_3_ (tmps).^12^

Photolysis of (NMe_4_)[Nb(CO)_6_] was carried out in the presence of one equivalent of tmps in THF at room temperature. A high-power blue light LED (*λ* = 465 nm) was used instead of a high-pressure mercury UV-lamp. The use of a nearly monochromatic source with an exciting wavelength close to the longest-wavelength absorption maximum of [Nb(CO)_6_]^–^ (*λ*_max_ = 440 nm in CH_2_Cl_2_)^13^ was conceived to be advantageous preventing the formation of insoluble brown decomposition products formed during the photolysis using a high-pressure mercury-lamp.11*a*

**Fig. 1:**
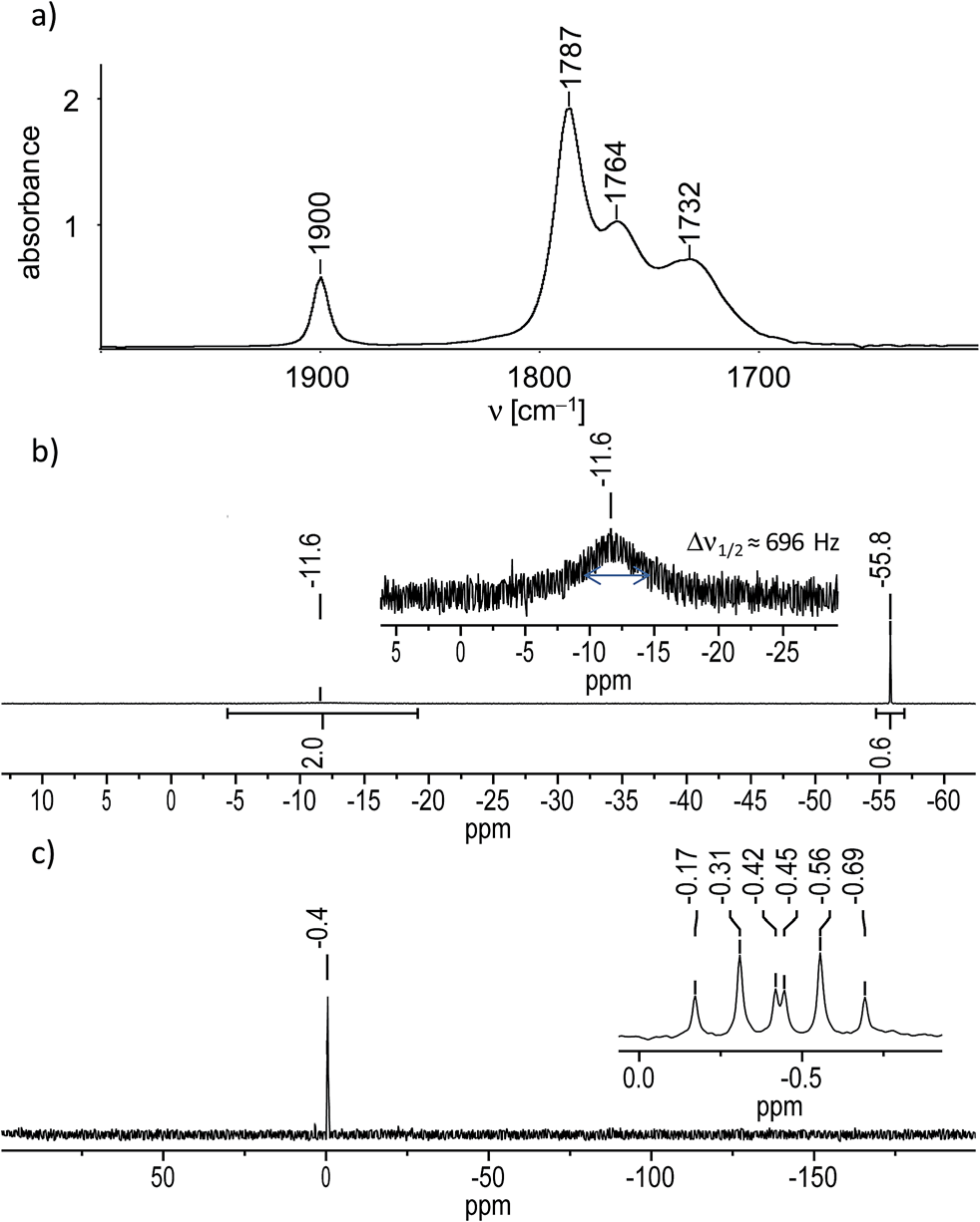
(a) FT-IR spectrum of **1** in THF in the range of 2000–1600 cm^–1^. (b) ^31^P{^1^H} NMR spectrum of **1** in benzene-*d*_6_; an enlarged excerpt with the broad signal at *δ* = –11.6 ppm is shown in the inset. (c) ^29^Si{^1^H} NMR spectrum of **1** in THF-*d*_8_; an enlarged excerpt with the signal at *δ* = –0.44 ppm is depicted in the inset.

In fact, IR-monitoring of the reaction revealed a slow, but very selective conversion into the tetracarbonyl niobate [Nb(CO)_4_(κ^2^-tmps)]^–^ proceeding *via* the pentacarbonyl intermediate [Nb(CO)_5_(κ^1^-tmps)]^–^ (*ν*(CO) in THF: 1966 (m), 1821 (vs) cm^–1^). After work-up the salt (NMe_4_)[Nb(CO)_4_(κ^2^-tmps)] (**1**) was isolated in nearly quantitative yield (97%) as an orange, analytically pure, very air-sensitive powder, which decolorizes immediately upon exposure to air. The salt decomposes upon heating at 142 °C to a dark brown mass, and is well soluble in acetonitrile and tetrahydrofurane (THF), but only moderately soluble in benzene, toluene, and diethyl ether. Attempts to grow suitable single crystals of **1** for an X-ray diffraction study failed, however unambiguous proof for the composition and structure of **1** was provided by elemental analysis, IR spectroscopy and ^1^H, ^13^C{^1^H}, ^31^P{^1^H} and ^29^Si{^1^H} NMR spectroscopy. The IR spectrum of **1** in THF displays four *ν*(CO) absorption bands at 1900, 1787, 1764 and 1732 cm^–1^ (Fig. 1a), the band pattern being typical for octahedral *cis*-disubstituted metal tetracarbonyl complexes with a local *C*_2v_ symmetry of the M(CO)_4_ fragment.^14^ All *ν*(CO) bands of **1** are shifted to lower frequencies than those of [Nb(CO)_4_(Ph_2_PCH_2_CH_2_PPh_2_)]^–^ (*ν*(CO) in THF = 1908, 1806, 1782 and 1746 cm^–1^) or related disubstituted arylphosphane-carbonyl niobates.11*b* This shift to lower frequencies evinces the stronger +I effect of the P-bonded alkyl substituents in **1**, which enhances the electron density at the metal center and leads to a stronger Nb(dπ) → CO(π*) backbonding and softening of the CO bonds in **1**. The NMR spectra of **1** corroborate the presence of an overall *C*_s_ symmetric complex, in which one of the arms of the tripodal ligand tmps is pendant and the other two arms are bonded to the niobium center. For example, the ^31^P{^1^H} NMR spectrum of **1** displays a sharp singlet for the ^31^P nucleus of the pendant CH_2_PMe_2_ arm, which appears at almost the same position (*δ*(P_A_) = –55.8 ppm in benzene-*d*_6_) as that of the non-coordinated (“free”) tmps (*δ*(P) = –55.1 ppm in benzene-*d*_6_), and a very broad signal for the two symmetry-equivalent Nb-bonded ^31^P nuclei at considerably lower field (*δ*(P_B_) = –11.6 ppm in benzene-*d*_6_) (Fig. 1b). The broadness of the second signal (Δ*ν*_1/2_ (full width at half maximum) = 696 Hz) is caused by the quadrupole moment of the ^93^Nb nucleus (*Q* = –0.32 × 10^–28^ m^2^; *I* = 9/2, 100% natural abundance) and its effect on the relaxation time.^15^ Further structural information was provided by the ^29^Si{^1^H} NMR spectrum of **1**, which shows a sharp signal for the bridgehead Si atom, that is split to a doublet of triplets (Fig. 1c) due to coupling with the two chemically different types of ^31^P nuclei in the integral ratio 1 : 2 (^2^*J*(Si,P_A_) = 14.7 Hz, ^2^*J*(Si,P_B_) = 8.2 Hz). A positional exchange of the pendant and the Nb-bonded arms of the tmps ligand in **1** was not observed in solution at 298 K.

**Scheme 4:**
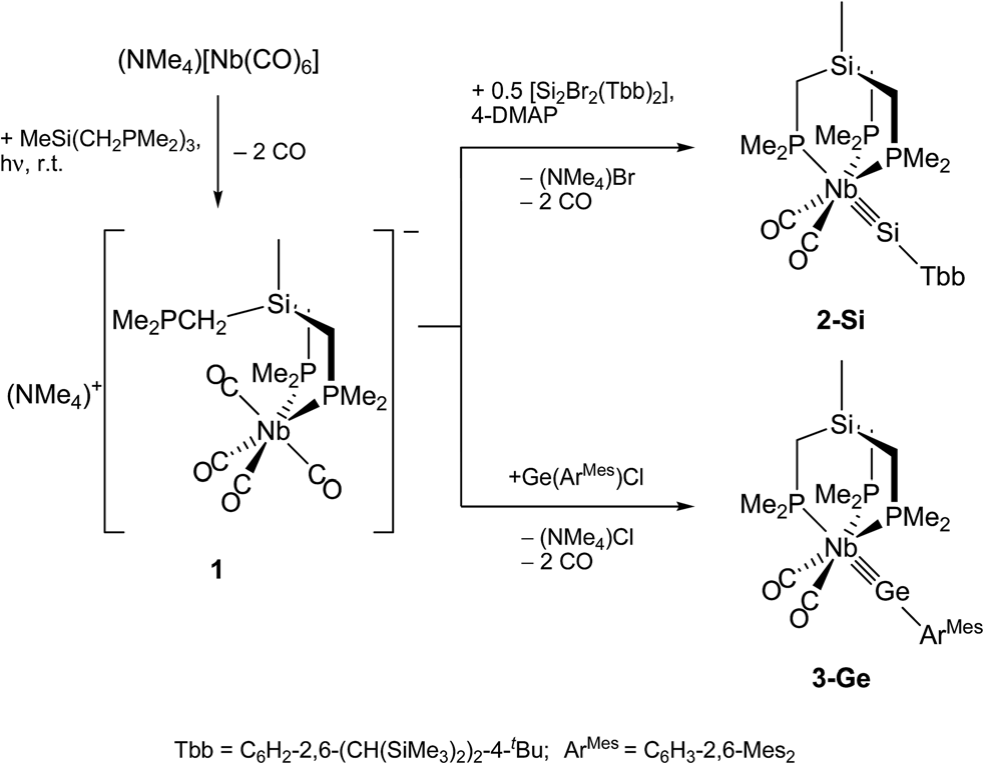
Synthesis of the niobium silylidyne complex **2-Si** and the germylidyne complex **3-Ge**.

Complex **1** was found to be a very suitable nucleophile for the formation of Nb≡E triple bonds (E = Si–Sn). Thus addition of a freshly prepared, orange-colored solution of a mixture of the 1,2-dibromodisilene *E*-Tbb(Br)Si=Si(Br)Tbb^16^ and 4-dimethylamino pyridine (4-DMAP) (molar ratio 1 : 4), to a solution of one equiv. of **1** in toluene at ambient temperature was accompanied by an immediate color change to red-brown, and precipitation of a white solid ((NMe_4_)Br). IR monitoring revealed a complete and selective conversion to the silylidyne complex [(κ^3^-tmps)(CO)_2_Nb≡Si–Tbb] (**2-Si**, Scheme 4). After work-up, complex **2-Si** was isolated in 59% yield as a red-brown, extremely air-sensitive, microcrystalline solid, which decolorizes immediately upon exposure to air. Compound **2-Si** is remarkably thermostable, and decomposes to a dark brown mass at 258 °C. It is moderately soluble in *n*-pentane, but readily soluble in benzene, toluene and THF.

Similarly, treatment of complex **1** with the *m*-terphenylgermanium(ii) chloride Ge(Ar^Mes^)Cl in toluene at –40 °C followed by warming to room temperature afforded rapidly and selectively the germylidyne complex [(κ^3^-tmps)(CO)_2_Nb≡Ge–Ar^Mes^] (**3-Ge**) (Scheme 4). Compound **3-Ge** was isolated as a deep-magenta, very air-sensitive, thermally stable powder (dec. at 284 °C), that is moderately soluble in benzene and toluene, and well soluble in THF. No evidence for the formation of the putative metallogermylene intermediate [(κ^3^-tmps)(CO)_3_Nb–GeAr^Mes^] could be obtained during IR monitoring of the reaction of **1** with Ge(Ar^Mes^)Cl in toluene, the reaction starting at –35 °C and proceeding rapidly with CO evolution below 0 °C.

**Scheme 5:**
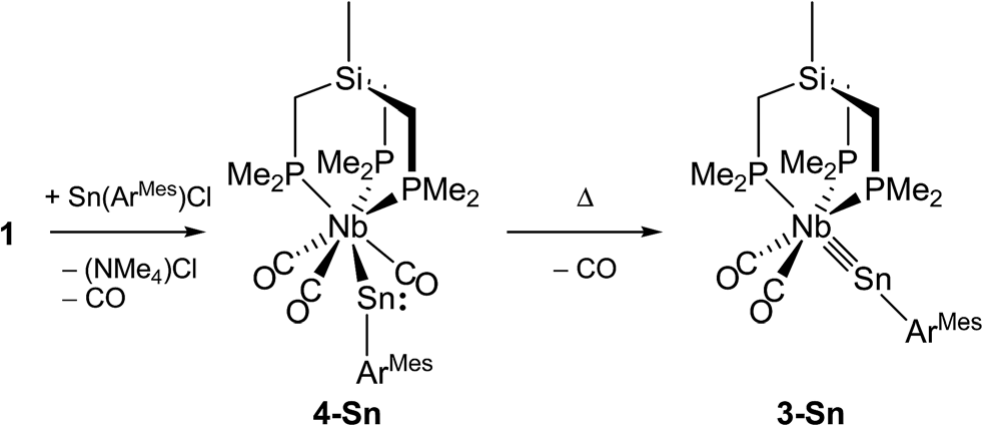
Synthesis of the niobium stannylidyne complex **3-Sn***via* the niobiastannylene **4-Sn**.

In comparison, reaction of the analogous *m*-terphenyltin(ii)chloride Sn(Ar^Mes^)Cl with **1** in toluene afforded after stirring at ambient temperature the brick-red metallostannylene [(κ^3^-tmps)(CO)_3_Nb–SnAr^Mes^] (**4-Sn**) with a small amount of the stannylidyne complex [(κ^3^-tmps)(CO)_2_Nb≡SnAr^Mes^] (**3-Sn**) (Scheme 5). Prolonged heating at 80 °C and periodic evacuation of the reaction tube was necessary to remove the released CO and to convert **4-Sn** almost quantitatively into the stannylidyne complex **3-Sn**, which after work-up was isolated as a dark violet, very air-sensitive powder in 70% yield. Complex **3-Sn** is as **3-Ge** thermally stable and decomposes upon heating at 266 °C. However, unlike **3-Ge**, complex **3-Sn** was found to be extremely light sensitive. Thus exposure of the deep-violet solutions of **3-Sn** to fluorescent, ambient light or sun light lead to deposition of a tin mirror and formation of tmps and 1,3-dimesitylbenzene as evidenced by ^1^H NMR spectroscopy. Therefore, all operations during the synthesis, isolation and characterization of **3-Sn** had to be carried out under exclusion of light.

Decarbonylation of **4-Sn** to afford **3-Sn** is a remarkable, new type of reaction in the chemistry of metallostannylenes. In fact previous attempts to transform the metallostannylenes [Cp(CO)_3_M–SnR] (M = Cr, Mo, W; R = Ar^Mes^, Ar^Trip^; Ar^Trip^ = C_6_H_3_–2,6-Trip_2_, Trip = C_6_H_2_–2,4,6-iPr_3_),^17^ [Cp(CO)_2_Fe–SnR] (R = Ar^Dipp^, Ar^Trip^; Ar^Dipp^ = C_6_H_3_–2,6-Dipp_2_, Dipp = C_6_H_3_–2,6-iPr_2_)^18^ or [Cp*(CO)_3_W–Sn(IDipp)]^+^ (Idipp = C[N(Dipp)CH]_2_, Dipp = C_6_H_3_–2,6-iPr_2_)2*n* into terminal stannylidyne complexes failed. We assume, that the increased steric pressure imposed by the tripodal ligand at the metal center weakens the Nb–CO bonds in the seven-coordinate complex **4-Sn** and decreases thereby the barrier for a CO dissociation. In addition, formation of a strong Nb≡Sn triple bond resulting from the higher energy and larger radial extension of the d orbitals, which are engaged in the Nb(dπ) → SnR(π*) back bonding, may be also a driving force for the reaction.

**Fig. 2:**
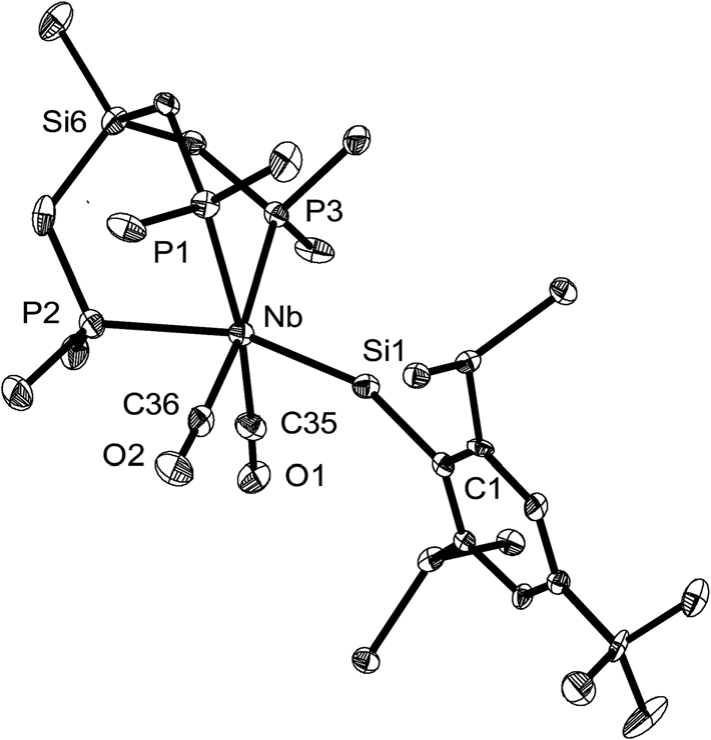
DIAMOND plot of the molecular structure of the silylidyne complex **2-Si** in the solid state. Thermal ellipsoids were set at 30% electronic probability at 100 K. Hydrogen atoms and the methyl groups of the C^2,6^–CH(SiMe_3_)_2_ substituents were omitted for clarity. Selected bond lengths [pm] and angles [°]: Nb–Si1 232.7(2), Nb–P1 259.9(2), Nb–P2 258.4(2), Nb–P3 259.3(2), Nb–C35 206.8(9), Nb–C36 206.3(7), Si1–C1 189.0(7), C35–O1 117.6(8), C36–O2 117.9(7); Nb–Si1–C1 159.2(2), P1–Nb–P2 85.61(7), P1–Nb–P3 85.31(6), P2–Nb–P3 87.92(6), C35–Nb–C36 93.3(3).

**Fig. 3:**
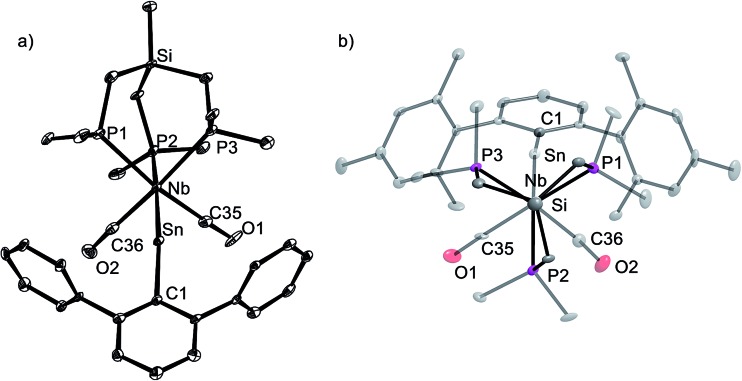
(a) DIAMOND plot of the molecular structure of the stannylidyne complex **3-Sn** (toluene) in the solid state. Thermal ellipsoids were set at 30% electronic probability at 100 K. Hydrogen atoms and methyl groups of the Ar^Mes^ substituent were omitted for clarity. Selected bond lengths [pm] and angles [°] of **3-Sn** (toluene) (bond lengths and angles for **3-Ge** (THF) are given in brackets): Nb–Sn 253.3(1) [235.79(4)], Nb–P1 260.6(4) [259.5(1)], Nb–P2 255.1(3) [258.0(1)], Nb–P3 258.6(4) [261.2(1)], Nb–C35 205.7(14) [206.0(5)], Nb–C36 207.1(16) [206.5(5)], Sn–C1 214.2(1) [196.3(4)], C35–O1 116.8(18) [116.8(6)], C36–O2 115.4(18) [115.5(6)]; Nb–Sn–C1 160.9(3) [164.0(1)], P1–Nb–P2 87.7(1) [86.30(4)], P1–Nb–P3 85.9(1) [86.08(4)], P2–Nb–P3 87.4(1) [87.82(4)], C35–Nb–C36 90.9(5) [92.5(2)]. (b) Top view of **3-Sn** along the Si···Nb vector illustrating the *C*_3_-symmetric twist of the tmps ligand.

The tetrylidyne complexes **2-Si**, **3-Ge** and **3-Sn** were characterized by elemental analyses, IR spectroscopy and ^1^H, ^13^C{^1^H}, ^31^P{^1^H}, ^29^Si{^1^H} and ^119^Sn{^1^H} NMR spectroscopy. In addition their molecular structures were determined by single-crystal X-ray crystallography (Fig. 2 and 3). All complexes are distorted octahedral and feature a tridentate (κ^3^-bonded) tmps ligand, which spans three facial coordination sites with the P–Nb–P bite angles varying in a small range (85.3–87.9°). A view along the Si···Nb vector reveals that the CH_2_ groups connecting the bridgehead Si atom with the P donors are twisted out creating a local *C*_3_ symmetric, right or left-handed conformation, which reduces the bite of the chelating triphosphane ligand and optimizes the bonding with the niobium center (Fig. 3b). In solution, however, a rapid interchange of the two conformational enantiomers occurs according to NMR spectroscopy leading to averaged *C*_s_ symmetric structures.

The tetrylidyne complexes **2-Si**, **3-Ge** and **3-Sn** feature the shortest Nb–Si, Nb–Ge and Nb–Sn bonds reported to date. In practice, the Nb–Si bond of **2-Si** (232.7(2) pm) is *ca.* 28 pm shorter than the Nb–Si single bonds of silyl complexes (*d*(Nb–Si)_mean_ of 28 structurally characterized complexes = 261.3 pm),^19^ and the Nb–Ge bond of **3-Ge** (235.79(4) pm) *ca.* 31 pm shorter than a Nb–Ge single bond (*d*(Nb–Ge)_mean_ = 267.3 pm).^20^ Similarly, the Nb–Sn bond of **3-Sn** (253.3(1) pm) is *ca.* 30 pm shorter than a Nb–Sn single bond (*d*(Nb–Sn)_mean_ 282.9 pm).^21^ Notably, a comparison of the Nb–E triple bond lengths of **2-Si**, **3-Ge** and **3-Sn** with those of related molybdenum tetrylidyne complexes (*e.g. d*(Mo≡Si) in [Cp(CO)_2_Mo≡Si–Ar^Trip^] = 222.41(7) pm;1*a**d*(Mo≡Ge) in [Cp(CO)_2_Mo≡Ge–R] (R = C(SiMe_3_)_3_, Ar^Mes^, Ar^Trip^) = 227–228 pm;2a,2b,2i,2m *d*(Mo≡Sn) in *trans*-[X(PMe_4_)Mo≡Sn–Ar^Mes^] (X = Cl, Br, I) = 248–249 pm)^22^) reveals that the differences in the M≡E triple bond lengths (E = Si: 10 pm; E = Ge: 8–9 pm; E = Sn: 5–6 pm) compare reasonably well with the difference (7 pm) of the metallic radii of the two elements (*r*_Nb_ = 147 pm, *r*_Mo_ = 140 pm; radii for a coordination number of 12).^23^ A series of additive triple bond radii for most elements of the periodic table have been predicted by P. Pyykkö *et al.*^24^ The experimental Nb–E triple bond lengths **2-Si**, **3-Ge** and **3-Sn**, are however, longer than the sum of the theoretically predicted triple bond radii (*d*(Nb≡E)_calc_ = Si: 218 pm, Ge: 230 pm, Sn: 248 pm).

In all complexes the tetrylidyne ligand is slightly bent at the tetrel center as evidenced by the bonding angle Nb–E–C1 (**2-Si**: 159.2(2)°, **3-Ge**: 164.0(1)°, **3-Sn**: 160.9(3)°). Bending occurs in all cases towards the CO ligands. It is presently unclear, whether this phenomenon, which is also observed in a series of group 6 metal dicarbonyl ylidyne complexes, is of steric or electronic origin or both. No clear evidence for steric congestion is at least provided by the molecular structures of **2-Si**, **3-Ge** and **3-Sn**. For example, the closest van der Waals contacts were found in **2-Si** between the methyl groups of the tmps ligand and the SiMe_3_ methyl groups of the Tbb substituent (*d*(H···H) = 244 pm). These contacts are longer than twice the van der Waals radius of hydrogen (*r*_vdW_(H) = 110 pm).^25^ It should be also taken into consideration, that deviation of the M≡E–R atom sequence from linearity does not require a lot of energy, indicating that subtle electronic effects may cause such a bending.^26^

**Fig. 4:**
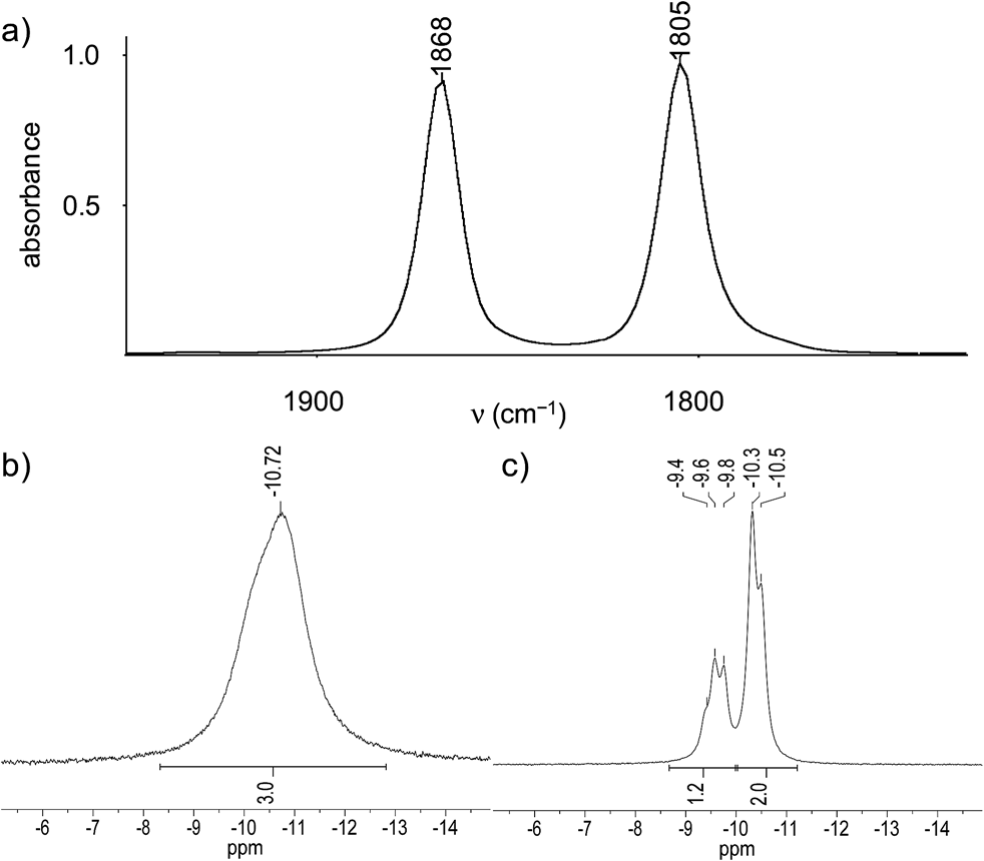
(a) IR *ν*(CO) absorption bands of the germylidyne complex **3-Ge** in toluene. (b) ^31^P{^1^H} NMR signal of the germylidyne complex **3-Ge** in THF-*d*_8_ at 283 K. (c) ^31^P{^1^H} NMR signals of the germylidyne complex **3-Ge** in THF-*d*_8_ at 193 K.

Further structural information was obtained from the IR and NMR spectra of the tetrylidyne complexes. The IR spectra of **2-Si**, **3-Ge** and **3-Sn** display two *ν*(CO) bands of almost equal intensity, which are typical for *cis*-dicarbonyl complexes and can be assigned to the in-phase (A′ symmetric) and out-of-phase (A′′ symmetric) CO stretching modes assuming local *C*_s_ symmetry of the M(CO)_2_ fragment (Fig. 4a). The *ν*(CO) bands of **3-Sn** appear at lower frequencies (1851 and 1791 cm^–1^ in toluene) than those of **3-Ge** (1868 and 1805 cm^–1^ in toluene), which suggests that the stannylidyne ligand SnAr^Mes^ has a higher σ-donor/π-acceptor ratio than the germylidyne ligand GeAr^Mes^. Notably, the *ν*(CO) bands of **2-Si** appear also at lower wavenumbers (1855 and 1790 cm^–1^ in toluene) than those of **3-Ge**. This shift can be rationalized with the stronger +I effect of the Tbb substituent, leading to a higher σ-donor/π-acceptor ratio of the silylidyne ligand SiTbb than that of the germylidyne ligand GeAr^Mes^. The low-frequency position of the *ν*(CO) bands of **2-Si**, **3-Ge** and **3-Sn** suggests the presence of an electron-rich Nb center that is engaged in strong Nb(dπ) → CO(π*) backbonding. Additional evidence for a strong Nb(dπ) → CO(π*) backbonding is provided by the ^13^C{^1^H} NMR spectra, which all display a broad CO signal at even lower field (*δ*_CO_ = 238.7 ppm (**2-Si**), 239.2 ppm (**3-Ge**), 238.9 ppm (**3-Sn**)) than that of **1** (*δ*_CO_ = 226.5 ppm).^27^ The number and relative intensity of the NMR signals indicate an averaged *C*_s_ symmetry of the tetrylidyne complexes in solution and a rapid rotation of the tetrel-bonded aryl group about the E–C_aryl_ bond. The signals of all nuclei directly attached to the quadrupolar ^93^Nb nucleus are significantly broadened due to fast relaxation (*vide supra*). For example, the ^29^Si{^1^H} NMR spectrum of **2-Si** displays at 298 K a very broad signal (Δ*ν*_1/2_ = 130 Hz) for the Nb≡Si nucleus at *δ* = 267.8 ppm, for which the ^2^*J*(^29^Si,^31^P) coupling could not be resolved. In comparison, the remote positioned bridgehead Si atom of the tmps ligand and the SiMe_3_ groups of the Tbb substituent give rise to sharp signals at *δ* = –0.7 ppm and +1.5 ppm, respectively, with the first of these signals being split into a quartet due to coupling to the three ^31^P nuclei (^2^*J*(^29^Si,^31^P) = 9.7 Hz) (Fig. S16 and S17 (ESI†)). Similarly, the ^31^P{^1^H} NMR spectra of **2-Si** and **3-Ge** show only one broad signal at *δ* = –13.0 ppm (Δ*ν*_1/2_ ≈ 182 Hz at 298 K) and –10.7 ppm (Δ*ν*_1/2_ ≈ 187 Hz at 283 K), respectively, instead of two ^31^P NMR signals expected for an AX_2_ spin system (Fig. 4b). Broadness of the signals can be influenced by the temperature given the well known relationship between the quadrupole-coupled nuclear relaxation time and the temperature dependent molecular correlation time.^28^ In fact, lowering of the temperature lead to a “decoupling” of the Nb nucleus and allowed to resolve the two ^31^P NMR signals and their ^2^*J*(P,P) coupling of 20.9 Hz as illustrated by the ^31^P NMR spectrum of **3-Ge** at 193 K (Fig. 4c). Taking advantage of the same effect, also the ^119^Sn resonance of **3-Sn**, that was not observable in THF-*d*_8_ at room temperature, could be detected at 243 K as a very broad signal (Δ*ν*_1/2_ ≈ 1297 Hz) at *δ* = 829.7 ppm (Fig. S36 (ESI†)).

First studies reveal a marked difference in the reactivity of the niobium germylidyne complex **3-Ge** and the related molybdenum germylidyne complexes [Cp(CO)_2_Mo≡Ge–R] (R = C(SiMe_3_)_3_, Ar^Mes^, Ar^Trip^). Thus treatment of [Cp(CO)_2_Mo≡Ge–R] with H_2_O or MeOH (one equiv.) in diethyl ether at 0 °C followed by warming to ambient temperature afforded within one hour selectively the brown hydroxy/methoxygermylidene complexes [Cp(CO)_2_(H)Mo=Ge(OR′)R] (R′ = H, Me), which were fully characterized.2*m* In contrast, no reaction of **3-Ge** with H_2_O (one equiv.) was observed in THF even at 60 °C. The inertness of **3-Ge** can be rationalized with the stronger metal-germylidyne Nb(dπ) → GeR(π*) back bonding, which reduces the electrophilicity of the Ge center in **3-Ge**, and increases in combination with the steric protection of the metal center by the tridentate tmps ligand the activation barrier for the H_2_O addition at the Nb≡Ge bond. In fact, a large excess of water (925 equiv.) and prolonged heating (3 h) was necessary to effectuate a full conversion of **3-Ge** accompanied by a color change of the reaction solution from magenta to orange. IR monitoring of the reaction did not provide any evidence for the formation of the anticipated H_2_O addition products. Instead, a continuous decrease in intensity of the two *ν*(CO) bands of **3-Ge** was observed suggesting the formation of mainly CO-free products. Benzene extraction of the orange-brown solid obtained after solvent evaporation afforded a benzene soluble, pale-orange part containing mainly the germanediol Ge(Ar^Mes^)H(OH)_2_, as well as a benzene-insoluble brownish part. The unprecedented hydridogermanediol^29^ was isolated as a pale yellow solid and characterized by IR and ^1^H NMR spectroscopy. Its IR spectrum displays two *ν*(OH) bands at 3600 and 3398 cm^–1^ and a characteristic *ν*(Ge–H) band at 2104 cm^–1^, the latter one appearing at a close position to that of GeBr_2_HMes (*ν*(Ge–H) = 2105 cm^–1^).^30^ In the ^1^H NMR spectrum a distinctive doublet signal is observed for the Ge(OH)_2_ protons at *δ* = 0.91 ppm and a triplet signal for the Ge–H functionality at *δ* = 5.61 ppm (^2^*J*(H,H) = 3.5 Hz) in the integral ratio of 2 : 1. Notably, the Ge–OH protons of the germanetriol Ge(Ar^Trip^)(OH)_3_ have a similar chemical shift (*δ* = 0.77 ppm in CDCl_3_).29*k*

**Scheme 6:**
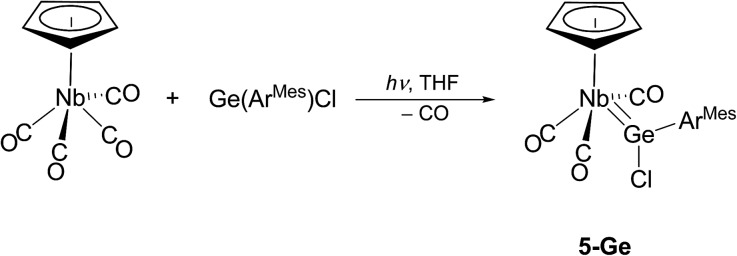
Synthesis of the niobium chlorogermylidene complex **5-Ge**.

Attempts were also undertaken to access cationic tetrylidyne complexes. For this purpose, [CpNb(CO)_4_]^31^ was prepared using a slightly modified procedure^32^ and irradiated in THF with a high-power blue light LED (*λ* = 465 nm) in the presence of one equivalent of Ge(Ar^Mes^)Cl. IR monitoring of the reaction revealed a quite selective decarbonylation leading to the chlorogermylidene complex **5-Ge**, which after work-up was isolated as red-orange, air-sensitive crystals in 25% yield (Scheme 6). Remarkably attempts to abstract the chloride from **5-Ge** and to form the putative germylidyne complex cation [Cp(CO)_3_Nb≡GeAr^Mes^]^+^ were not successful so far. For example, no reaction of **5-Ge** with Na[B(Ar^F^)_4_] (Ar^F^ = C_6_H_3_–3,5-(CF_3_)_2_) was observed in C_6_H_5_F at room temperature.

**Fig. 5:**
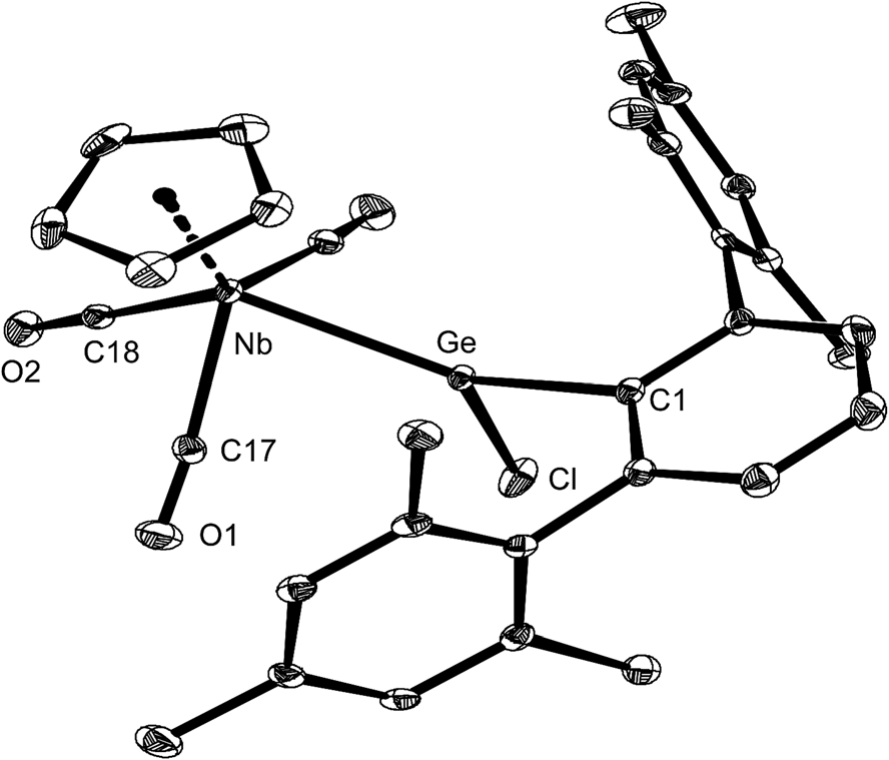
DIAMOND plot of the molecular structure of **5-Ge** in the solid state. Thermal ellipsoids were set at 30% electronic probability at 100 K, and hydrogen atoms were omitted for clarity. Selected bond lengths [pm] and angles [°]: Nb–Ge 251.78(6), Ge–C1 196.2(4), Ge–Cl 219.1(1), Nb–C17 207.4(3), Nb–C18 206.1(4), C17–O1 115.2(4), C18–O2 114.5(5); Nb–Ge–C1 141.4(1), Nb–Ge–Cl 118.75(4), Cl–Ge–C1 99.8(1).

Complex **5-Ge** is the first niobium germylidene complex to be reported. Its solid-state molecular structure was determined by single-crystal X-ray crystallography (Fig. 5). The four-legged piano stool complex is *C*_s_ symmetric and features a trigonal–planar coordinated Ge-atom (sum of angles at the Ge atom = 360.0°). The symmetry plane passes through the atoms Nb, Ge, C1 and Cl, and bisects the CpNb(CO)_3_ fragment and the central ring of the *m*-terphenyl substituent.

The germylidene ligand adopts an upright conformation, with the Ar^Mes^ substituent pointing towards the cyclopentadienyl ring. The Nb–Ge distance (251.78(6) pm) of **5-Ge** lies in-between that found for the Nb–Ge triple bond of **3-Ge** (235.79(4) pm) (*vide supra*) and that of a Nb–Ge single bond (*d*(Nb–Ge) = 267.3 pm)^20^ indicating the presence of a Nb–Ge double bond in **5-Ge**. The angles at the Ge atom differ markedly with the Nb–Ge–C_aryl_ angle (141.4(1)°) being much larger than the C_aryl_–Ge–Cl angle (99.8(1)°). This distortion can be attributed to the large steric demand of the Ar^Mes^ substituent and the low tendency of germanium for isovalent hybridization.1a,1b,2l,3d The Ge–Cl bond of **5-Ge** (219.1(1) pm) compares well with that of Ge(Ar^Trip^)Cl (220.3(2) pm),^33^ but is considerably shorter than those of chlorogermylidene complexes containing electron-rich metal centers, such as [(dmpe)_2_Fe=Ge(Ar^Mes^)Cl] (*d*(Ge–Cl) = 232.2(1) pm),^5^ [(PMe_3_)_3_Ni=Ge(Ar^Mes^)Cl] (*d*(Ge–Cl) = 230.03(8) pm)^6^ or [(PMe_3_)_3_Pd=Ge(Ar^Mes^)Cl] (*d*(Ge–Cl) = 227.3(1) pm),^6^ in which a strong M(dπ) → Ge(pπ) back bonding is presumed to cause a strong polarization of Ge–Cl bond leading to a facile chloride abstraction by Lewis acids. The reduced polarization of the Ge–Cl bond of **5-Ge** provides a rationale for its inertness towards mild chloride abstraction reagents.

The solution IR and NMR spectra of **5-Ge** are fully consistent with its solid-state molecular structure. Thus, the IR spectrum of **5-Ge** in THF displays three intense *ν*(CO) absorption bands at 1980, 1910 and 1899 cm^–1^, as expected for a Nb(CO)_3_ fragment with local *C*_s_ symmetry, which are assigned to the A′ (all three CO modes in phase), A′ (two CO_lat_ modes in phase; CO_diag_ mode out-of-phase) and A′′ symmetric (two CO_lat_ modes out-of-phase) CO stretching modes, respectively. The *ν*(CO) absorption bands of **5-Ge** are high-frequency shifted compared to those of [CpNb(CO)_3_THF] (*ν*(CO) in THF = 1961, 1840 cm^–1^)^31^ or [CpNb(CO)_3_PEt_3_] (*ν*(CO) in THF = 1953, 1850 cm^–1^),28*b* but appear at roughly the same position as those of [CpNb(CO)_3_N_2_] (*ν*(CO) in *n*-heptane = 1991, 1905 cm^–1^)^34^ suggesting a similar σ-donor/π-acceptor ratio of the germylidene GeAr^Mes^Cl and the N_2_ ligand. The ^1^H and ^13^C{^1^H} NMR spectra also confirm the *C*_s_ symmetry of **5-Ge** in solution. Rotation of the *m*-terphenyl substituent about the Ge–C_aryl_ bond occurs fast on the NMR time-scale at ambient temperature leading to an exchange of the two diastereotopic *ortho* (C^2,6^) and *meta* (C^3,5^) positions of the enantiotopic mesityl substituents. Therefore, only one singlet signal is observed in the ^1^H NMR spectrum of **5-Ge** for the C^2,6^-bonded methyl groups and C^3,5^-bonded protons of the mesityl substituents, respectively.

### Electrochemical studies

**Fig. 6:**
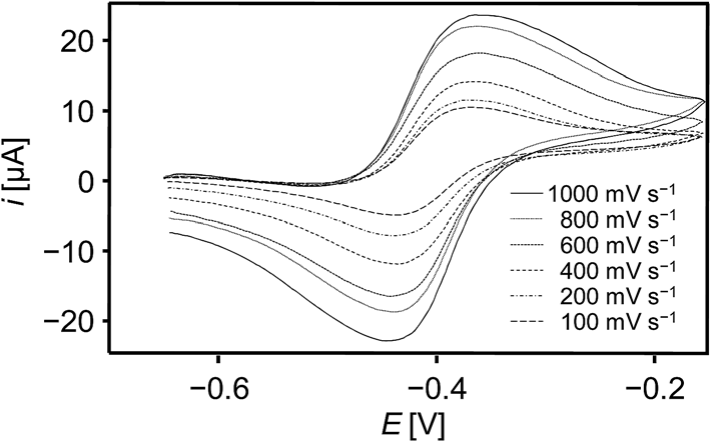
Single-scan cyclic voltammograms of the reversible one-electron oxidation of **3-Ge** at different scan rates in THF at –11 °C (supporting electrolyte: [NBu_4_][PF_6_] (0.1 M); reference electrode: 0.004 M [Fe(C_5_Me_5_)_2_]^+1/0^/0.1 M [NBu_4_][PF_6_]/THF).

Electrochemical studies of the tetrylidyne complexes **2-Si**, **3-Ge** and **3-Sn** were carried out using cyclic voltammetry to elucidate the redox properties of these compounds. All complexes display a rich electrochemistry involving several electron-transfer steps (see ESI, chapter 3†). Remarkably, both the one-electron reduction and oxidation of the germylidyne complex **3-Ge** are electrochemically reversible occurring at a half wave potential (*E*_1/2_) of –2.612 mV and –405 mV *vs.* the dmfc^1+/0^ redox couple (dmfc = decamethylferrocene), respectively (Fig. 6).^35^

In comparison, the corresponding redox steps of **2-Si** and **3-Sn** are irreversible (ESI, chapter 3†), but one-electron oxidation **2-Si** and **3-Sn** occurs at similar potentials as that of **3-Ge** (**2**: *E*_pa_ + *E*_pc_/2 = –468 mV, **3-Sn**: *E*_pa_ + *E*_pc_/2 = –435 mV (scan rate = 100 mV s^–1^)). Evidence that the redox process at *E*_1/2_ = –405 mV involves a one electron oxidation of **3-Ge** was provided by chemical means. Thus, no reaction of **3-Ge** with the one-electron reducing agent cobaltocene (*E*_1/2_ of CoCp_2_ in DME = –740 mV) was observed in fluorobenzene even at 70 °C, whereas an instantaneous oxidation of **3-Ge** occurred upon treatment with one equivalent of [Fe(η^5^-C_5_Me_5_)_2_][B(Ar^F^)_4_] in fluorobenzene solution at –30 °C. Unfortunately, attempts to isolate the putative germylidyne complex radical cation [(κ^3^-tmps)(CO)_2_Nb(GeAr^Mes^)]^+^ failed so far.^36^ Notably, the redox potential for the one-electron oxidation of **3-Ge** is slightly lower than that of the molybdenum tetrylidyne complexes *trans*-[ClMo(PMe_3_)_4_≡E–Ar^Mes^] (E = Ge: *E*_1/2_ in C_6_H_5_F = –340 mV; E = Sn: *E*_1/2_ in THF = –350 mV; E = Pb: *E*_1/2_ in THF = –358 mV) verifying the presence of an electron-rich Nb center in **3-Ge**.

## Conclusion

The synthesis of the tailor-made carbonyl-niobate (NMe_4_)[Nb(CO)_4_(κ^2^-tmps)] allowed to explore its reactivity towards a series of organotetrel(ii) halides, which lead to the isolation of the first niobium complexes featuring triple bonds with the elements Si, Ge and Sn. Photochemical CO substitution in [CpNb(CO)_4_] (Cp = η^5^-C_5_H_5_) by Ge(Ar^Mes^)Cl afforded also the novel chlorogermylidene complex [Cp(CO)_3_Nb=Ge(Ar^Mes^)Cl]. The structural, spectroscopic and electrochemical data of the tetrylidyne complexes [(κ^3^-tmps)(CO)_2_Nb≡Si–Tbb] (**2-Si**), [(κ^3^-tmps)(CO)_2_Nb≡Ge–Ar^Mes^] (**3-Ge**) and [(κ^3^-tmps)(CO)_2_Nb≡Sn–Ar^Mes^] (**3-Sn**) suggest the presence of an electron-rich metal center that is engaged into strong metal (dπ) → ER(π*) and metal (dπ) → CO(π*) back bonding. Remarkably, one-electron oxidation and reduction of the germylidyne complex **3-Ge** are electrochemically reversible.

## Supplementary Material

Supplementary information

Crystal structure data
