# Supplementary material for: Triple bonds of niobium with silicon, germaniun and tin: the tetrylidyne complexes [(κ3-tmps)(CO)2Nb[triple bond, length as m-dash]E–R] (E = Si, Ge, Sn; tmps = MeSi(CH2PMe2)3; R = aryl)
Source: Chem Sci. 2017 Jul 14;8(9):6290–9. doi: 10.1039/c7sc02708g (PMC5956830; doi:10.1039/c7sc02708g)
Supplement: Supplementary file 1 [file SC-008-C7SC02708G-s001.pdf]

Electronic Supplementary Material (ESI) for Chemical Science.
This journal is © The Royal Society of Chemistry 2017

Supporting Information

Content

1 Experimental Section – General part S2
2 Syntheses and analytical data of compounds S4
2.1 $(NMe_4)[Nb(CO)_4(\kappa^2\text{-tmps})]$ (**1**) S4
2.2 $[(\kappa^3\text{-tmps})(CO)_2Nb{\equiv}SiTbb]$ (**2-Si**) S10
2.3 $[(\kappa^3\text{-tmps})(CO)_2Nb{\equiv}GeAr^{Mes}]$ (**3-Ge**) S16
2.4 $[(\kappa^3\text{-tmps})(CO)_2Nb{\equiv}SnAr^{Mes}]$ (**3-Sn**) S23
2.5 Reaction of **3-Ge** with water S30
2.6 $[Cp(CO)_3Nb{=}Ge(Ar^{Mes})Cl)]$ (**5-Ge**) S31
3 Cyclic Voltammetry S33
4 Crystal structure determination S38
4.1 Crystal data and structure refinement for **2-Si** S39
4.2 Crystal data and structure refinement for **3-Ge**(THF) S40
4.3 Crystal data and structure refinement for **3-Sn**(toluene) S41
4.4 Crystal data and structure refinement for **5-Ge** S42

1 Experimental Section – General part

All experiments were carried out in an argon atmosphere under the strict exclusion of oxygen and moisture using Schlenk or glove box techniques. Argon was commercially received with a purity of ≥ 99.999 % and passed through a gas purification system composed of two consecutive columns to remove traces of water and oxygen. The first column was filled with BASF R 3-11G catalyst and heated at 100 °C and the second column with 4 Å molecular sieves. All glassware was dried at 110 °C in a drying oven, baked out under vacuum and filled with the dry inert gas during cooling prior to use. Brown glassware was employed during the synthesis of **3-Sn** due to its light sensitivity. Each solvent was heated to reflux over a suitable drying agent, purged several times with argon during reflux and distilled under argon. The following drying agents were used: sodium wire in the presence of benzophenone and tetraglyme (0.5 vol%) for *n*-pentane, *n*-hexane, petroleum ether 40/60, sodium wire for benzene and toluene, and sodium wire with some benzophenone for tetrahydrofurane (THF), diethyl ether and 1,2-dimethoxyethane (DME). All solvents were degassed by two freeze-pump-thaw cycles and stored in the glove box. Water was freed from oxygen by purging slowly with argon for one hour.

The C, H, N elemental analyses were carried out on an Elementar Vario Micro analyser in triplicate for each sample. The individual C, H, and N values did not differ by more than ± 0.3 %. The mean C, H, and N values are given for each compound. Melting points were determined on a Büchi M-560 melting point apparatus. Internal temperature correction was done by calibration against the melting points of 4-nitro toluene, diphenylacetic acid, caffeine and potassium nitrate. The samples were sealed in glass capillaries under vacuum and heated once with a temperature gradient of 5 $Kmin^{-1}$ for a rough determination of the melting point or temperature of starting decomposition. Heating of the second and third sample was then repeated with a gradient of 2 $Kmin^{-1}$ starting 20 K below the temperature of melting or decomposition determined in the first experiment. Decomposition of the compounds was verified by visual inspection.

The mid-infrared spectra of solutions were recorded on a Nicolet 380 FT-IR spectrometer or a Bruker Vector 22 FT-IR spectrometer using a stainless steel cell with NaCl windows and were background corrected for the solvent absorptions. The NaCl windows were separated by a teflon spacer with a thickness of 0.2 mm. The mid-infrared spectra of solids were recorded on a Bruker Alpha FT-IR spectrometer using a diamond single-reflection platinum-ATR-module in a glove box. The intensities and shape of the IR absorption bands were abbreviated as follows: vs (very strong), s (strong), m (medium), w (weak), vw (very weak), sh (shoulder). The NMR spectra were recorded on a Bruker Avance DMX-300, DPX-300, DPX-400 or a DMX-500 NMR spectrometer in dry, deoxygenated benzene-d_6, tetrahydrofurane-d_8 or acetonitrile-d_3. Benzene-d_6 and tetrahydrofurane-d_8 were dried upon

stirring over Na/K-alloy and acetonitrile-d_3 over CaH_2. After drying, the deuterated solvents were trap-to-trap condensed and stored over molecular sieves (4 Å). The ^{1}H NMR spectra were calibrated against the residual proton resonances and the $^{13}C\{^{1}H\}$ NMR spectra against the natural abundance ^{13}C resonances of the corresponding deuterated solvent relative to tetramethylsilane ($SiMe_4$) (C_6D_6: δ_H = 7.15 ppm, δ_C = 128.0 ppm; CD_3CN: δ_H = 1.93 ppm, δ_C = 118.2 ppm; THF-d_8: δ_H = 1.73 ppm, δ_C = 25.3 ppm). The $^{31}P\{^{1}H\}$ NMR spectra were calibrated externally against a 85 % aqueous solution of H_3PO_4, and the $^{29}Si\{^{1}H\}$ and $^{119}Sn\{^{1}H\}$ NMR spectra against pure $SiMe_4$ and $SnMe_4$, respectively. The NMR standards were sealed in capillaries and measured in vacuum-sealed 5 mm NMR tubes containing the cooresponding deuterated solvent. The multiplicities and shape of the NMR signals are given using the following abbreviations: s (singlet), d (doublet), dd (doublet of doublets) t (triplet), dt (doublet of triplets), q (quartet), dec (decet), m (multiplet), br (broad). The full width at half maximum intensity of broadened signals is designated with $\Delta\nu_{1/2}$. The ^{1}H and ^{13}C NMR signals of the compounds **1**, **2-Si**, **3-Ge** and **3-Sn** were assigned by a combination of HMQC, HMBC and DEPT experiments. This allowed an unequivocal assignment of all proton and carbon resonances including those of the diastereotopic P-bonded methyl groups and methylene protons of **1**, **2-Si**, **3-Ge** and **3-Sn**, which were labeled with the subscript letters A and B, respectively. The label A was always used for the methyl group with the lower ^{1}H chemical shift.

The triphosphane $MeSi(CH_2PMe_2)_3$ (tmps),[S1] the 1,2-dibromodisilene *E*-Tbb(Br)Si=Si(Br)Tbb[S2] (Tbb = 4-*tert*-butyl-2,6-bis(bis(trimethylsilyl)methyl)phenyl) and the aryltin(II) chloride $[Sn(Ar^{Mes})Cl]_2$[S3] (Ar^{Mes} = 2,6-mesitylphenyl; mesityl (Mes) = 2,4,6-trimethylphenyl) were prepared as described in the corresponding reference. The synthesis of $[Ge(Ar^{Mes})Cl]_2$ reported by P. Power et al.[S3] could not be reproduced and had to be modified to afford the germanium(II) compound in high yield as a bright orange to yellow solid depending on the crystallization conditions. For simplicity reasons the monomeric formulas $E(Ar^{Mes})Cl$ (E = Ge, Sn) were used in the text for the compounds, which forms dimers in the solid-state.[S3] The purity of all starting materials was checked by NMR spectroscopy. The tetramethylammonium niobate $(NMe_4)[Nb(CO)_6]$ was prepared following the procedure used for the synthesis of the tetraethylammonium salt $(NEt_4)[Nb(CO)_6]$[S4] and isolated as a canary yellow solid, in 34 % yield. $(NMe_4)[Nb(CO)_6]$ was characterized by

[S1] H. H. Karsch, A. Appelt, *Z. Naturforsch. B* **1983**, *38*, 1399–1405.

[S2] P. Ghana, M. I. Arz, U. Das, G. Schnakenburg, A. C. Filippou, *Angew. Chem. Int. Ed.* **2015**, *54*, 9980–9985; *Angew. Chem.* **2015**, *127*, 10118–10123.

[S3] R. S. Simons, L. Pu, M. M. Olmstead, P. P. Power, *Organometallics* **1997**, *16*, 1920–1925.

[S4] C. G. Dewey, J. E. Ellis, K. L. Fjare, K. M. Pfahl, G. F. P. Warnock, *Organometallics* **1983**, *2*, 388–391.

elemental analysis, IR spectroscopy, ^{1}H NMR and $^{13}C\{^{1}H\}$ NMR spectroscopy.[S5] [CpNb(CO)$_4$] was prepared following a slightly modified procedure from that reported for [(η^5-C$_9$H$_7$)Nb(CO)$_4$][S6] and isolated as an orange-red, crystalline solid in 75 % yield. The procedure involved oxidation of (NEt$_4$)[Nb(CO)$_6$] with one equiv. of I_2 in THF at 0 °C leading to (NEt$_4$)[(CO)$_4$Nb(μ-I)$_3$Nb(CO)$_4$], which was then directly treated with 1.5 equiv. of NaCp at 0 °C. The purity of [CpNb(CO)$_4$] was confirmed by IR, ^{1}H and $^{13}C\{^{1}H\}$ NMR spectroscopy.[S7]

2 Syntheses and analytical data of compounds

2.1 (NMe$_4$)[Nb(CO)$_4$(κ^2-tmps)] (1)

A Schlenk tube was charged with a yellow solution of (NMe$_4$)[Nb(CO)$_6$] (680 mg, 2.03 mmol) and tmps (592 mg, 2.21 mmol, 1.09 eq.) in 80 mL of THF and then closed with a mercury bubbler. The reaction mixture was vigorously stirred and irradiated with two external LED lamps (λ = 465 nm, P = 2.5 W each), which were placed at a distance of 2 cm from the Schlenk tube. After a few minutes, the color of the solution changed to orange and gas (CO) was released via the mercury bubbler. During photolysis a colorless precipitate deposited on the glass surface of the reaction vessel and was removed mechanically after 14 h by use of a strong magnet and the magnetic stirring bar. The course of the reaction was followed by FT-IR spectroscopy and completion of the reaction after 38 h of irradiation was confirmed by FT-IR spectroscopy.[S8] The obtained orange turbid solution was filtered and the filtrate concentrated to approximately 1 mL under reduced pressure. 50 mL of a 1:1 mixture of petrol ether 40/60 and diethyl ether were slowly added at –30 °C to the stirred orange solution to precipitate the product. The solution was filtered off and the precipitate washed with a 1:1 mixture of petrol ether 40/60 and diethyl ether (3 × 17 mL) and dried under vacuum to yield complex **1** as an orange powder. Yield: 1.08 g (1.97 mmol, 97 %)

[S5] Spectroscopic data of (NMe$_4$)[Nb(CO)$_6$]: IR (CH$_3$CN, cm^{-1}): $\tilde{\nu}$ = 1864 (vs) (ν_{CO}). IR (THF, cm^{-1}): $\tilde{\nu}$ = 1894 (w, sh), 1862 (vs) (ν_{CO}). ^{1}H NMR (400.1 MHz, CD$_3$CN, 298 K, ppm): δ = 3.06 (t, ^{2}J(H,^{14}N) = 0.6 Hz, 12H, N*Me$_4$*$^+$). $^{13}C\{^{1}H\}$ NMR (100.1 MHz, CD$_3$CN, 298 K, ppm): δ = 56.3 (t, ^{1}J(C,^{14}N) = 4.1 Hz, 4C, N*Me$_4$*$^+$), 217.5 (dec, ^{1}J(Nb,C) = 236 Hz, 6C, 6 x *C*O). Elemental analysis calcd. (%) for $C_{10}H_{12}NNbO_6$ (335.11 g mol^{-1}): C 35.84, H 3.61, N 4.18; found: C 35.92, H 3.38, N 4.16.

[S6] T. E. Bitterwolf, S. Gallagher, J. T. Bays, B. Scallorn, A. L. Rheingold, I. A. Guzei, L. Liable-Sands, J. C. Linehan, *J. Organomet. Chem.* **1998**, *557*, 77–92.

[S7] Spectroscopic data of [CpNb(CO)$_4$]: IR (THF, cm^{-1}): $\tilde{\nu}$ = 2031 (s), 1918 (vs) (ν_{CO}). ^{1}H NMR (300.1 MHz, benzene-*d$_6$*, 298 K, ppm): δ = 4.72 (br, $\Delta\nu_{1/2}$ = 12 Hz, 5H, C$_5$*H$_5$*). $^{13}C\{^{1}H\}$ NMR (125.8 MHz, benzene-*d$_6$*, 298 K, ppm): δ = 94.7 (br, $\Delta\nu_{1/2}$ = 113 Hz, 5C, *C$_5$*H$_5$). The ^{13}C NMR signal of the carbonyl ligands could not detected after 10k scans at a concentration of 0.46 mmol mL^{-1}.

[S8] An intermediate was formed during photolysis, which displayed two ν(CO) absorption bands at 1966 and 1821 cm^{-1}. This intermediate is suggested to be (NMe$_4$)[Nb(CO)$_5$(κ^1-tmps)].

The salt is insoluble in *n*-pentane and *n*-hexane, moderately soluble in benzene, toluene and diethyl ether and good soluble in THF and acetonitrile. It turns into a dark brown mass at 142 °C and also decomposes upon heating in boiling toluene to give a brown precipitate, which is not IR active in the region of the carbonyl stretching vibrations. Elemental analysis calcd. (%) for $C_{18}H_{39}NNbO_4P_3Si$ (547.42 g mol^{-1}): C 39.49, H 7.18, N 2.56; found: C 38.93, H 7.13, N 2.51.

IR (THF, cm^{-1}, *Figure S1*): $\tilde{\nu}$ = 1900 (m), 1787 (vs), 1764 (m), 1732 (m) (ν_{CO}).

IR (toluene, cm^{-1}): $\tilde{\nu}$ = 1897 (m), 1783 (vs), 1761 (m), 1727 (m) (ν_{CO}).

IR (solid, cm^{-1}, *Figure S2 and Figure S3*): $\tilde{\nu}$ = 3028 (w), 2958 (w), 2892 (w), 2857 (w), 2806 (w), 1891 (m, ν_{CO}), 1739 (s, ν_{CO}), 1706 (vs, ν_{CO}), 1684 (vs, ν_{CO}), 1481 (m), 1445 (m), 1413 (m), 1362 (w), 1287 (w), 1274 (m), 1249 (m), 1103 (sh), 1093 (m), 1031 (m), 945 (sh), 933 (m), 897 (s), 863 (m), 832 (m), 816 (w), 788 (m), 765 (m), 737 (m), 712 (m), 702 (m), 680 (m), 641 (w), 588 (m), 554 (s), 525 (m), 461 (w), 433 (w), 406 (m).

^{1}H NMR (300.1 MHz, THF-d_8, 298 K, ppm, *Figure S4*): δ = 0.23 (s, 3H, Si*Me*), 0.69 (d, ^{2}J(H,P) = 2.5 Hz, 2H, CH_2PMe$_2$), 0.96 – 0.99 (m, 4H, 2 × CH_AH_BPMe$_A$Me$_B$), 1.00 (d, ^{2}J(H,P) = 3.1 Hz, 6H, CH$_2$PMe_2), 1.36 – 1.37 (m, 6H, 2 × CH$_A$H$_B$PMe_AMe$_B$), 1.37 – 1.39 (m, 6H, 2 × CH$_A$H$_B$PMe$_A$$Me_B$), 3.38 (s, 12H, N$Me_4^+$).

^{1}H NMR (300.1 MHz, benzene-d_6, 298 K, ppm): δ = 0.28 (s, 3H, Si*Me*), 0.52 (d, ^{2}J(H,P) = 2.5 Hz, 2H, CH_2PMe$_2$), 0.91 (d, ^{2}J(H,P) = 3.1 Hz, 6H, CH$_2$PMe_2), 0.95 – 0.99 (m, 4H, 2 × CH_AH_BPMe$_A$Me$_B$), 1.58 – 1.59 (m, 6H, 2 × CH$_A$H$_B$PMe_AMe_B), 1.64 – 1.65 (m, 6H, 2 × CH$_A$H$_B$PMe_AMe_B), 2.70 (s, 12H, NMe_4^+).[S9]

^{13}C{^{1}H} NMR (100.6 MHz, THF-d_8, 298 K, ppm, *Figure S5* and *Figure S6*): δ = 0.9 (dt, ^{3}J(C,P) = 4.6 Hz, ^{3}J(C,P) = 2.4 Hz, 1C, Si*Me*), 18.4 (d, ^{1}J(C,P) = 15.2 Hz, 2C, CH$_2$PMe_2), 22.1 (dt, ^{1}J(C,P) = 28.7 Hz, ^{3}J(C,P) = 4.0 Hz, 1C, *C*H$_2$PMe$_2$), 22.5 (dd, ^{1}J(C,P) = 4.2 Hz, ^{3}J(C,P) = 0.8 Hz, 2C, 2 × *C*H$_A$H$_B$PMe$_A$Me$_B$), 25.3 (dd, ^{1}J(C,P) = 9.3 Hz, ^{3}J(C,P) = 6.8 Hz, 2C, 2 × CH$_A$H$_B$PMe_AMe$_B$), 26.5 (dd, ^{1}J(C,P) = 9.4 Hz, ^{3}J(C,P) = 7.6 Hz, 2C, 2 × CH$_A$H$_B$PMe$_A$$Me_B$), 56.4 (t, ^{1}J(C,^{14}N) = 4.0 Hz, 4C, NMe_4^+), 226.5 (br, $\Delta\nu_{1/2}$ ≈ 186 Hz, 4C, 4 × *C*O).

^{31}P{^{1}H} NMR (121.5 MHz, THF-d_8, 298 K, ppm): δ = −55.6 (s, 1P, CH$_2$*P*Me$_2$), −10.9 (br, $\Delta\nu_{1/2}$ ≈ 1300 Hz, 2P, 2 × CH$_A$H$_B$*P*Me$_A$Me$_B$).

^{31}P{^{1}H} NMR (121.5 MHz, benzene-d_6, 298 K, ppm, *Figure S7*): δ = −55.8 (s, 1P, CH$_2$*P*Me$_2$), −11.6 (br, $\Delta\nu_{1/2}$ ≈ 696 Hz, 2P, 2 × CH$_A$H$_B$*P*Me$_A$Me$_B$).

^{29}Si{^{1}H} NMR (59.63 MHz, THF-d_8, 298 K, ppm, *Figure S8*): δ = −0.44 (dt, ^{2}J(Si,P) = 14.7 Hz, ^{2}J(Si,P) = 8.2 Hz, 1Si, *Si*Me).

[S9] Due to the moderate solubility of **1** in benzene-d_6, no ^{13}C{^{1}H} NMR and ^{1}H-^{13}C correlated NMR spectra were recorded in this solvent.

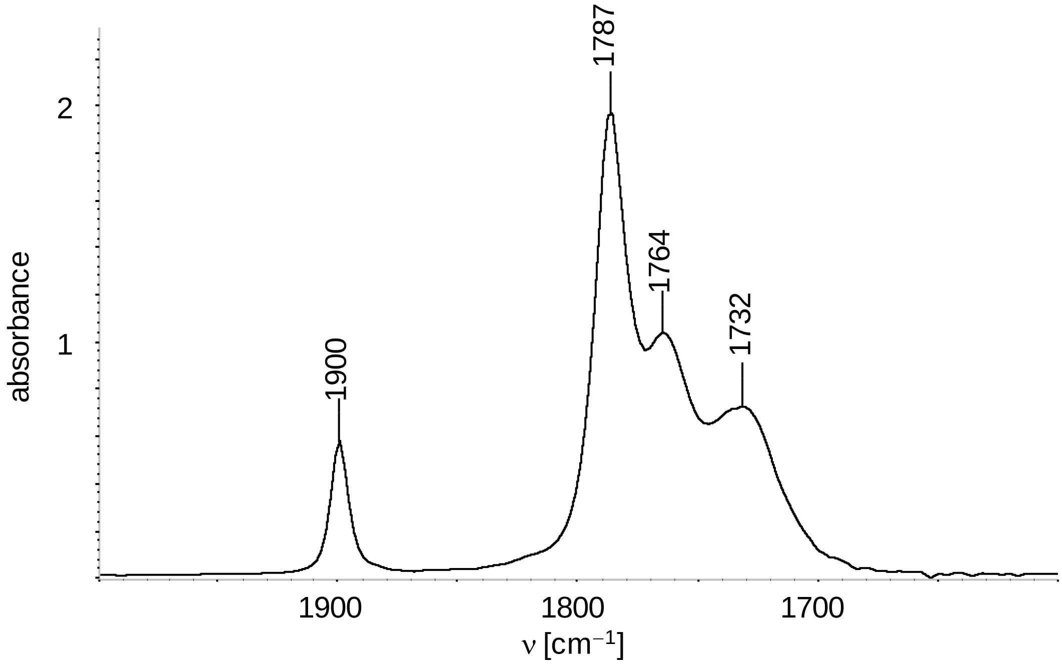

Figure S1. FT-IR spectrum of compound **1** in THF in the range of 2000 – 1600 cm^{-1}.

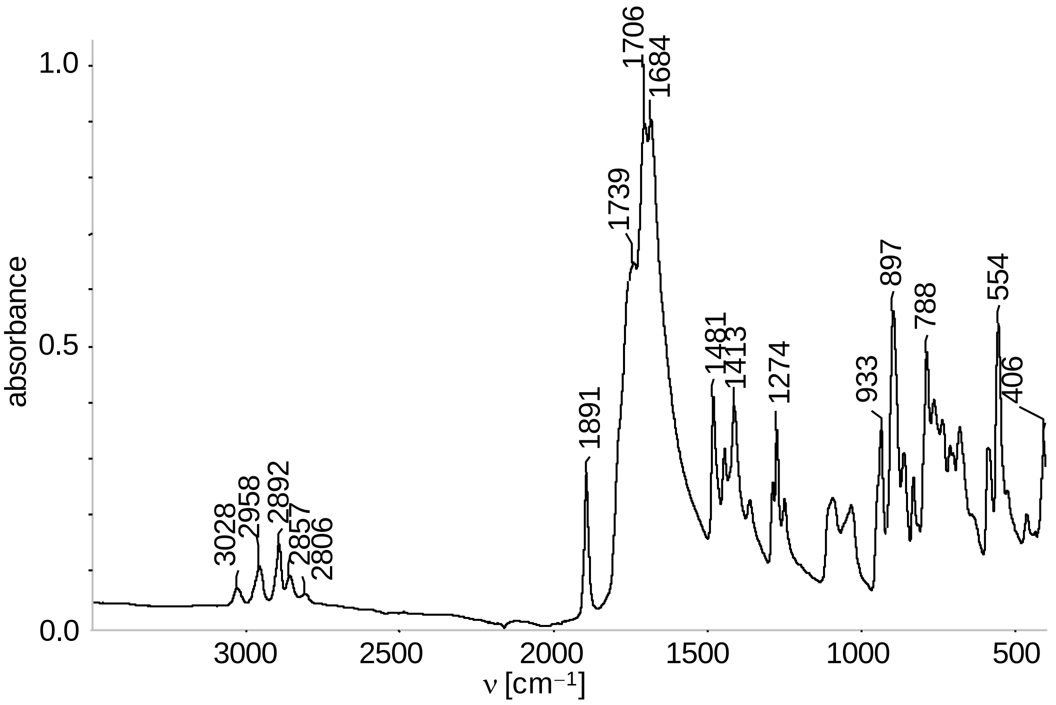

Figure S2. Solid state FT-IR spectrum of compound **1** in the range of 3500 – 400 cm^{-1}.

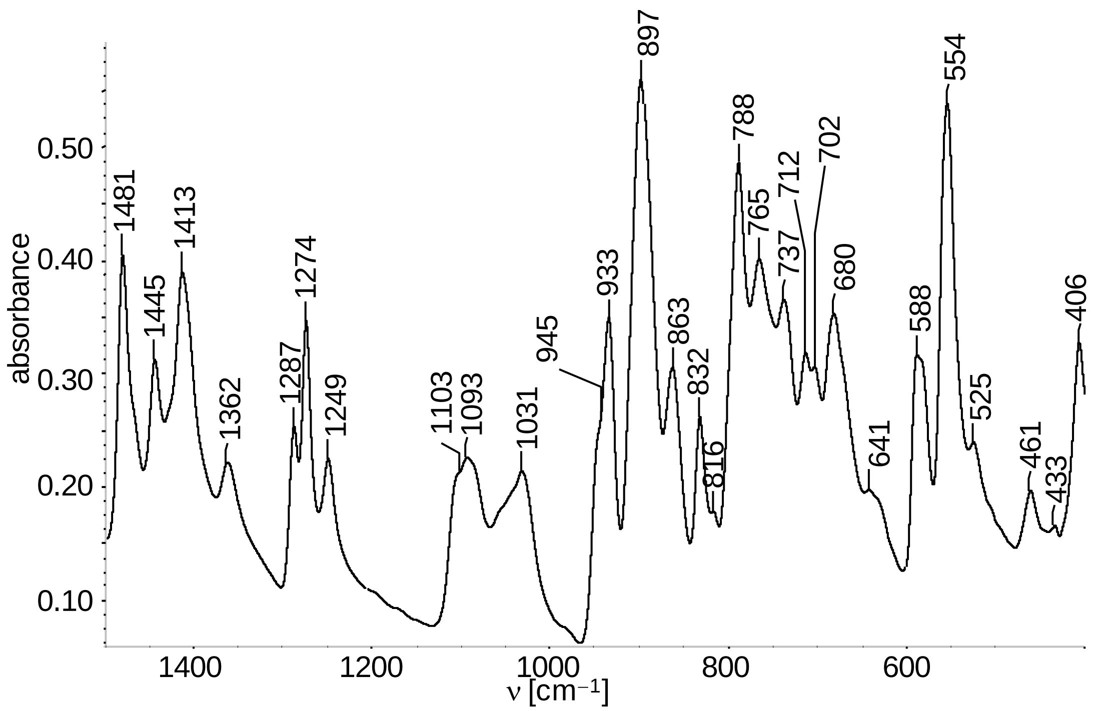

Figure S3. Expanded section (1500 – 400 cm^{-1}) of the solid-state FT-IR spectrum of compound **1** shown in *Figure S2*.

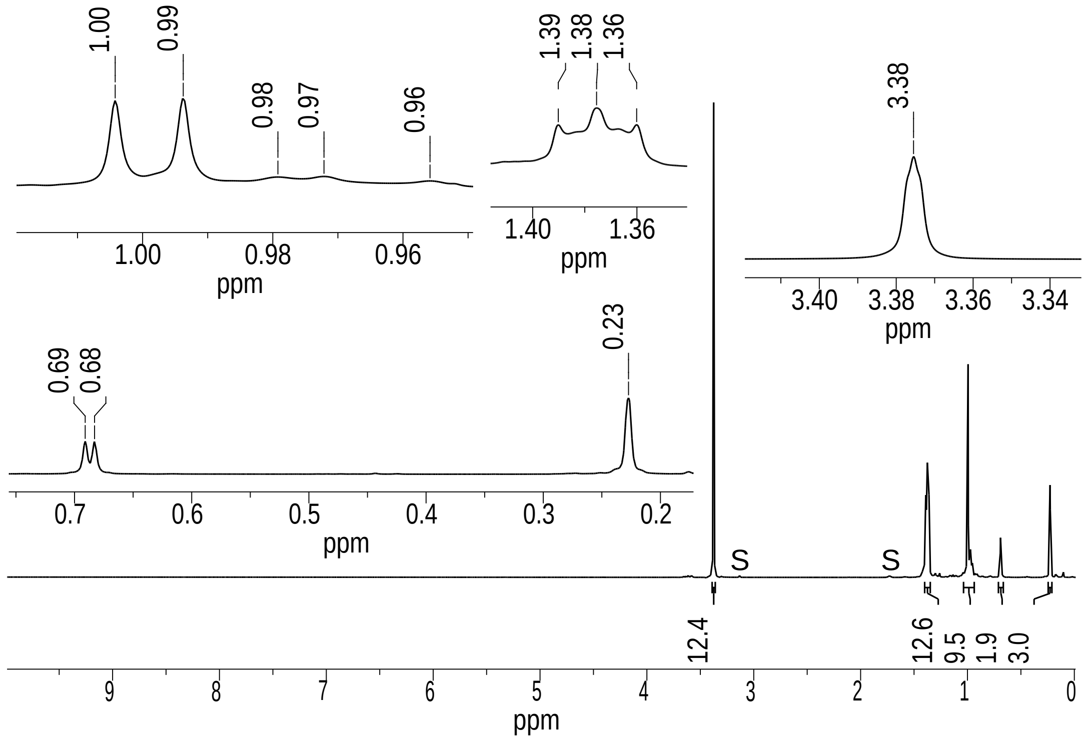

Figure S4. ^{1}H NMR spectrum (300.1 MHz) of compound **1** in THF-d_8 at 298 K; the character S marks the residual proton signals of the deuterated solvent. Enlarged excerpts are shown in the insets.

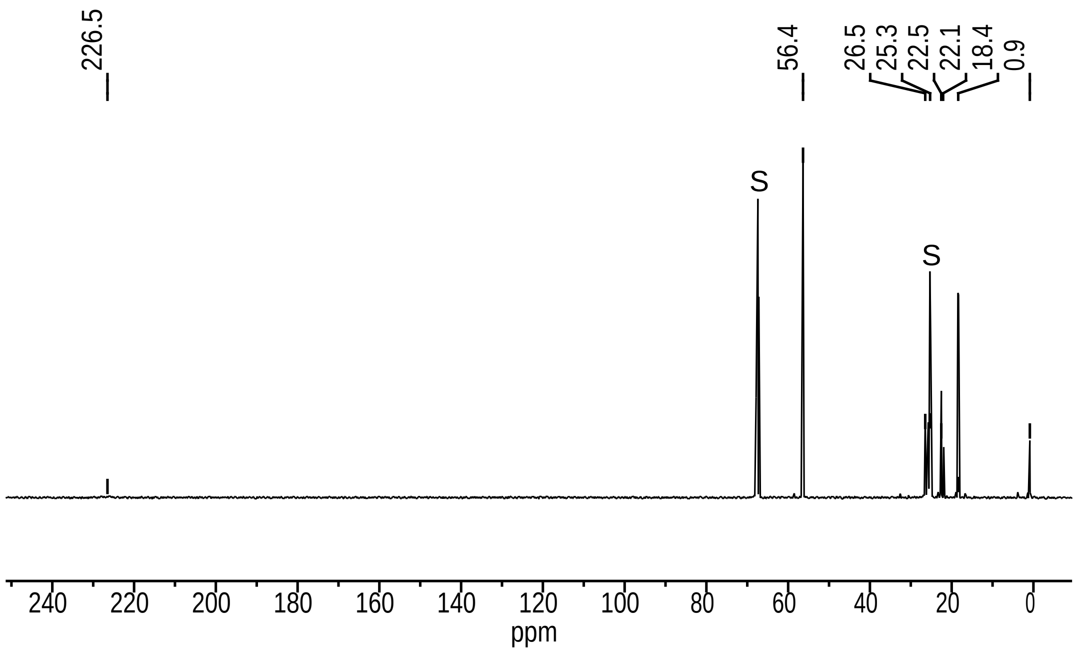

Figure S5. $^{13}C\{^1H\}$ NMR spectrum (100.6 MHz) of compound **1** in THF-d_8 at 298 K; the character S marks the ^{13}C signals of the deuterated solvent.

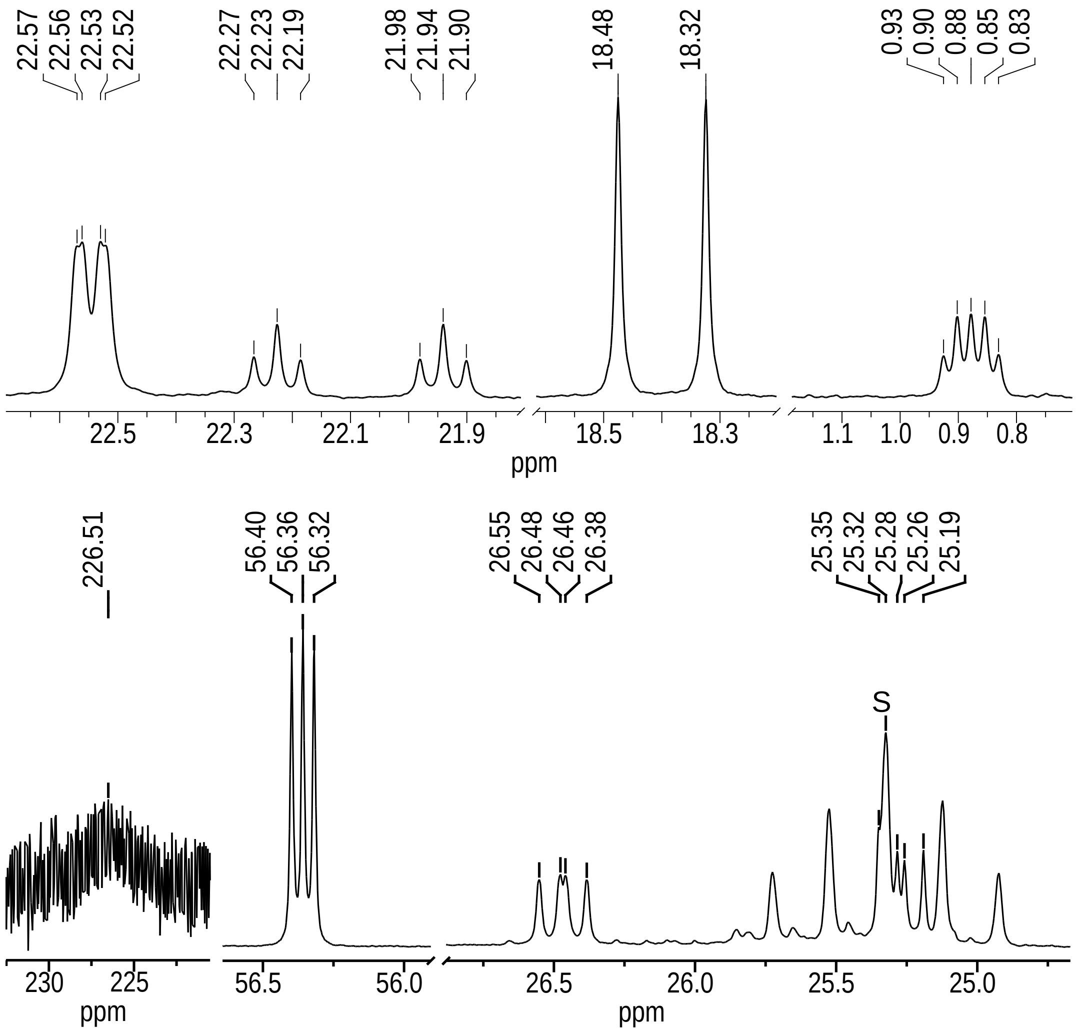

Figure S6. Expanded sections of the $^{13}C\{^{1}H\}$ NMR spectrum (100.6 MHz) of compound **1** in THF-d_8 at 298 K shown in *Figure S5*; the character S marks the natural abundance ^{13}C signal of the deuterated solvent.

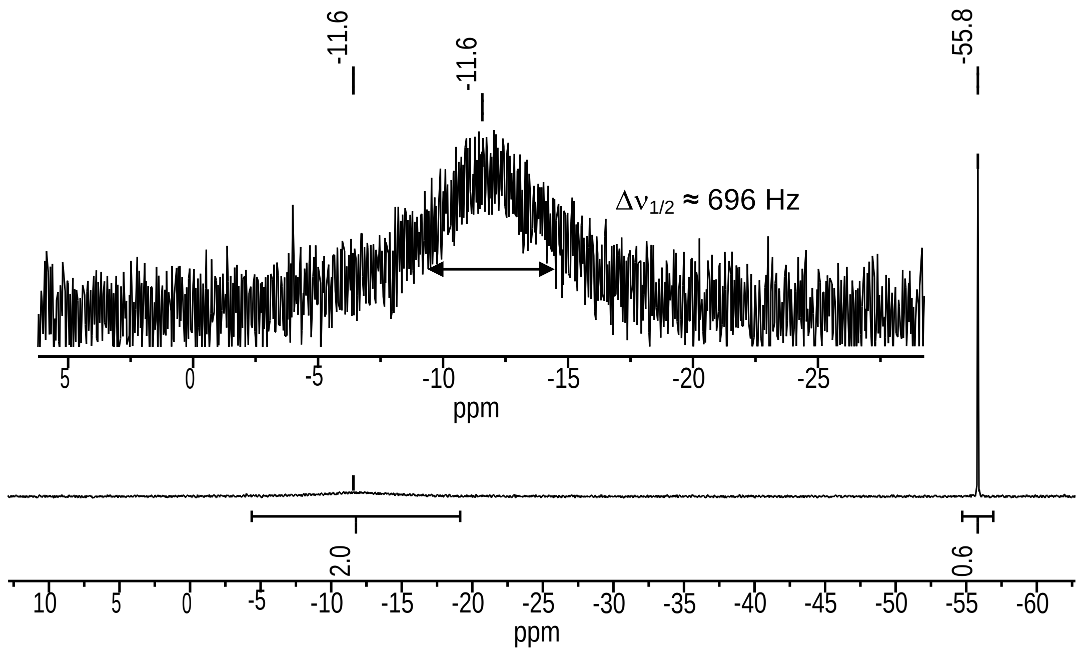

Figure S7. $^{31}P\{^{1}H\}$ NMR spectrum (121.5 MHz) of compound **1** in benzene-d_6 at 298 K. An enlarged excerpt of the spectrum is shown in the inset.

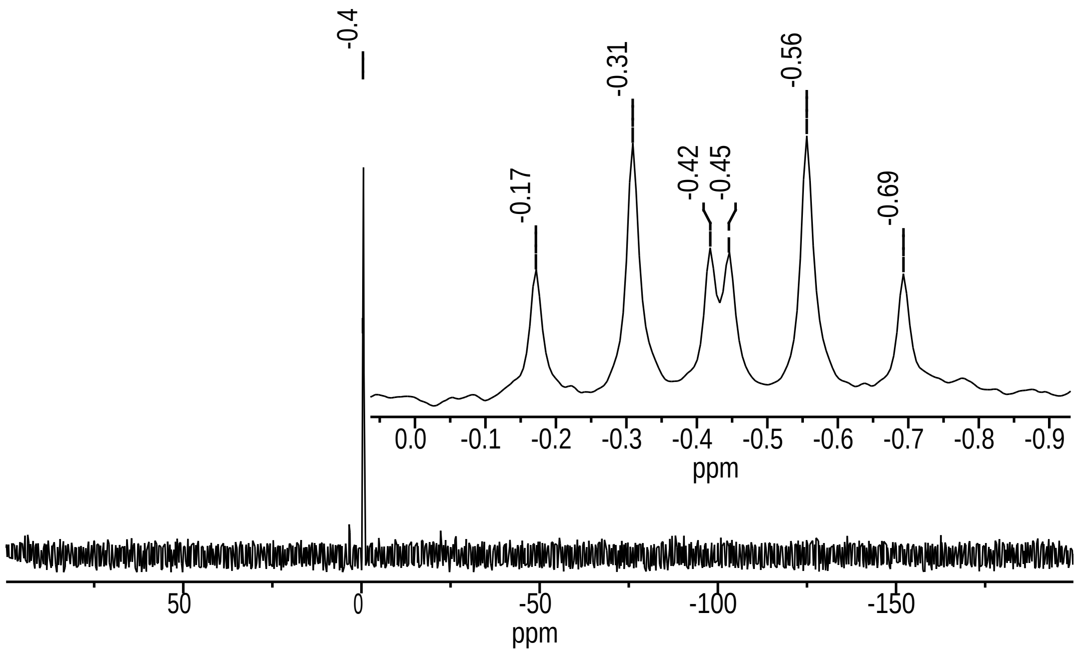

Figure S8. $^{29}Si\{^{1}H\}$ NMR spectrum (59.63 MHz) of compound **1** in THF-d_8 at 298 K. An enlarged excerpt of the spectrum is shown in the inset.

2.2 [(κ^3-tmps)(CO)$_2$Nb≡SiTbb] (2-Si)

A freshly prepared orange solution of the disilene *E*-Tbb(Br)Si=Si(Br)Tbb (516 mg, 0.46 mmol, 0.5 eq.) and 4-dmap (223 mg,1.82 mmol, 2.0 eq.) in 15 mL of toluene was added at room temperature within ten minutes to a stirred, orange solution of **1** (500 mg (0.91 mmol, 1.0 eq.) in 15 mL of toluene. The color of the reaction mixture turned immediately red-brown,

and a colorless solid precipitated out of the solution. The reaction mixture was stirred for additional 1.5 h, and the complete and selective conversion of the starting material **1** to the silylidyne complex **2-Si** was confirmed by FT-IR spectroscopy. The solvent was removed under reduced pressure, and the obtained red-brown residue was lyophilized. 4-DMAP (200 mg) was removed by vacuum sublimation at 80 °C for three hours. The remaining solid was extracted with a 1:2 mixture of petrol ether 40/60 and toluene (3 × 5 mL), and the combined red-brown extracts were concentrated under reduced pressure to approximately 3 mL. Storage of the solution at − 30 °C over night yielded only a small amount of crystalline material and rapid cooling of the solution to − 78 °C for ten minutes resulted in the separation of a brown oil. Therefore, the solvent was removed *in vacuo* again, and the residue was crystallized from 1.5 mL of a toluene/*n*-hexane mixture (1:2) at − 30 °C. The brown, microcrystalline solid was collected by filtration at − 30 °C, washed with *n*-pentane (2 × 2 mL) at the same temperature and dried for two hours under vacuum at ambient temperature to afford the silylidyne complex **2-Si** as a red-brown, microcrystalline solid. Yield: 480 mg (0.54 mmol, 59 %).[S10]

Compound **2-Si** is moderately soluble in *n*-pentane and *n*-hexane, and readily soluble in benzene, toluene, diethyl ether and THF. It turns into a dark brown mass upon heating to 258 °C.

Elemental analysis calcd. (%) for $C_{36}H_{76}NbO_2P_3Si_6$ (895.33 g mol^{-1}): C 48.29, H 8.56; found: C 47.64, H 8.42.

IR (toluene, cm^{-1}, *Figure S9*): $\tilde{\nu}$ = 1855 (s), 1790 (vs) (ν_{CO}).

IR (solid, cm^{-1}, *Figure S10* and *Figure S11*): $\tilde{\nu}$ = 2948 (w), 2895 (w), 2868 (w), 1848 (s, ν_{CO}), 1780 (vs, ν_{CO}), 1582 (w), 1523 (w), 1476 (vw), 1461 (vw), 1417 (w), 1406 (w), 1393 (w), 1360 (w), 1288 (w), 1276 (w), 1256 (m), 1242 (m), 1202 (vw,sh), 1162 (w), 1103 (vw), 1081 (w), 1028 (w), 1013 (w), 953 (w), 937 (m), 905 (m), 889 (m), 856 (m,sh), 832 (vs), 798 (m), 757 (s), 722 (m), 683 (m), 660 (m), 643 (w), 627 (m), 618 (m), 604 (m), 578 (w), 559 (m), 529 (m), 497 (m), 489 (m), 475 (m), 434 (w,sh), 420 (m).

^{1}H NMR (300.1 MHz, benzene-d_6, 298 K, ppm, *Figure S12*): δ = − 0.19 (q, ^4J(H,P) ≈ 0.8 Hz, 3H, Si*Me*), 0.13 (br, $\Delta\nu_{1/2}$ ≈ 31 Hz, 2H, *trans*-CH_2PMe$_2$), 0.26 (br, $\Delta\nu_{1/2}$ ≈ 28 Hz, 4H, 2 × *cis*-CH_AH_BPMe$_A$Me$_B$), 0.39 (s, ^1J(^{13}C,H) = 118.8 Hz, 36H, 2 x CH(SiMe_3)$_2$, Tbb), 1.28 (br, $\Delta\nu_{1/2}$ ≈ 18 Hz, 6H, *trans*-CH$_2$PMe_2), 1.32 (s, 9H, CMe_3, Tbb), 1.42 (br, $\Delta\nu_{1/2}$ ≈ 11 Hz, 12H, 2 × *cis*-CH$_A$H$_B$P$Me_A$$Me_B$), 3.84 (s, 2H, 2 × C*H*(SiMe$_3$)$_2$, Tbb), 6.88 (s, 2H, $C^{3,5}$-*H*, Tbb).

^{13}C{^{1}H} NMR (75.47 MHz, benzene-d_6, 298 K, ppm, *Figure S13 and Figure S14*): δ = 0.7 (q, ^3J(C,P) = 6.7 Hz, 1C, Si*Me*), 1.0 (s, 12C, 2 × CH(SiMe_3)$_2$, Tbb), 15.1 (s, 3C, 2 × *cis*-*C*H$_A$H$_B$PMe$_A$Me$_B$ + *trans*-*C*H$_2$PMe$_2$), 22.1 (br, $\Delta\nu_{1/2}$ ≈ 37 Hz, 2C, *trans*-CH$_2$PMe_2), 25.2

[S10] No further attempts were undertaken to obtain another crop of the compound from the combined red-brown mother liquor and washing solutions.

(br, $\Delta\nu_{1/2}$ ≈ 33 Hz, 2C, 2 × *cis*-$CH_AH_BPMe_AMe_B$), 29.9 (s, 2C, 2 × *C*H(SiMe$_3$)$_2$, Tbb), 31.3 (s, 3C, C*Me$_3$*, Tbb), 31.6 (br, $\Delta\nu_{1/2}$ ≈ 16 Hz, 2C, 2 × *cis*-$CH_AH_BPMe_AMe_B$), 34.7 (s, 1C, *C*Me$_3$, Tbb), 121.3 (s, 2C, $C^{3,5}$-H, Tbb), 148.0 (s, 2C, $C^{2,6}$, Tbb), 150.56 (s, 1C, C^4, Tbb), 150.63 (br, 1C, C^1, Tbb),[S11] 238.7 (br, $\Delta\nu_{1/2}$ ≈ 54 Hz, 2C, 2 × *C*O).

^{31}P{^{1}H} NMR (121.5 MHz, benzene-*d*$_6$, 298 K, ppm, *Figure S15*): δ = − 13.0 (br, $\Delta\nu_{1/2}$ ≈ 182 Hz, 3P, 2 x *cis*-$CH_AH_BPMe_AMe_B$, *trans*-CH_2PMe_2).

^{29}Si{^{1}H} NMR (59.63 MHz, benzene-*d*$_6$, 298 K, ppm, *Figure S16* and *Figure S17*): δ = − 0.7 (q, ^2J(Si,P) = 9.7 Hz, 1Si, *Si*Me), 1.5 (s, 4Si, 2 × CH(*Si*Me$_3$)$_2$, Tbb), 267.8 (br, Δ $\nu_{1/2}$ ≈ 130 Hz, 1Si, Nb≡*Si*).

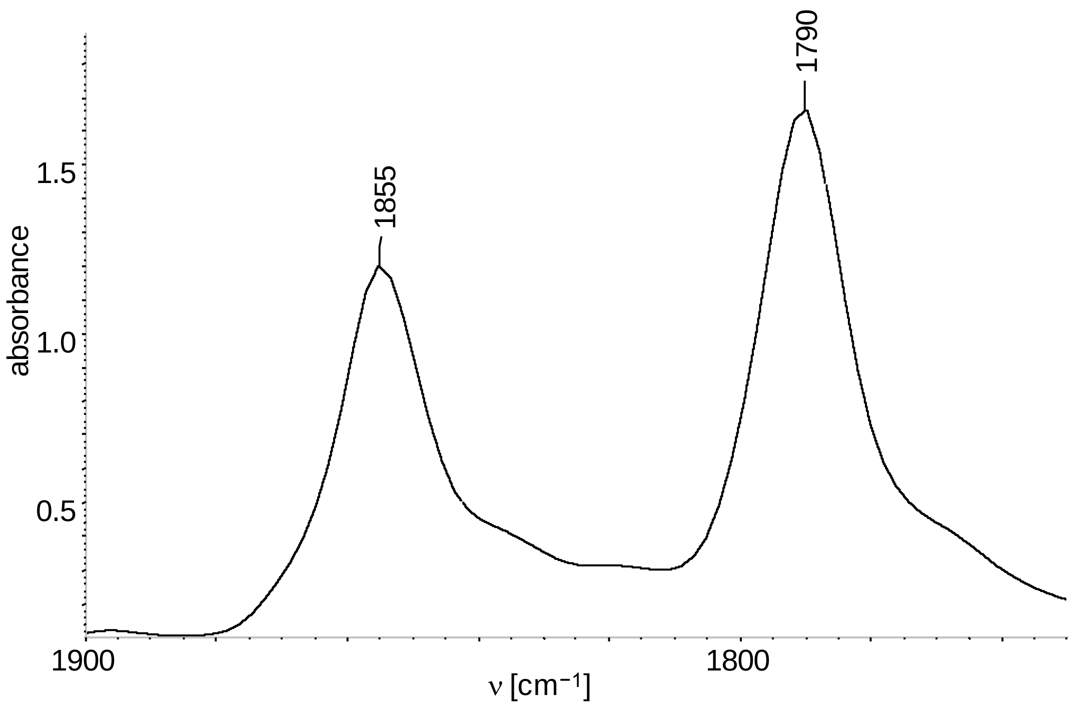

Figure S9. FT-IR spectrum of compound **2-Si** in toluene in the range of 1900 – 1750 cm^{-1}.

[S11] The broad signal of the C^1 carbon atom at δ = 150.63 ppm appearing next to the sharp signal of the C^4 carbon atom at δ = 150.56 ppm (*Figure S14*) was unambiguously assigned by ^{1}H-^{13}C correlation spectroscopy.

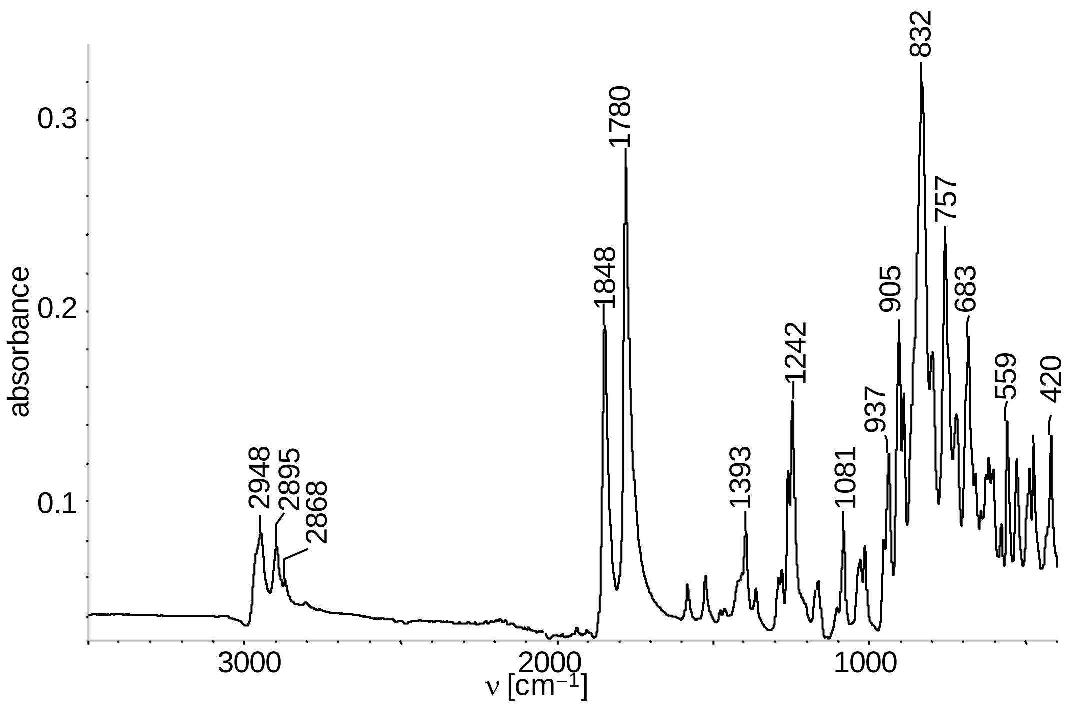

Figure S10. Solid state FT-IR spectrum of compound **2-Si** in the range of 3500 – 400 cm^{-1}.

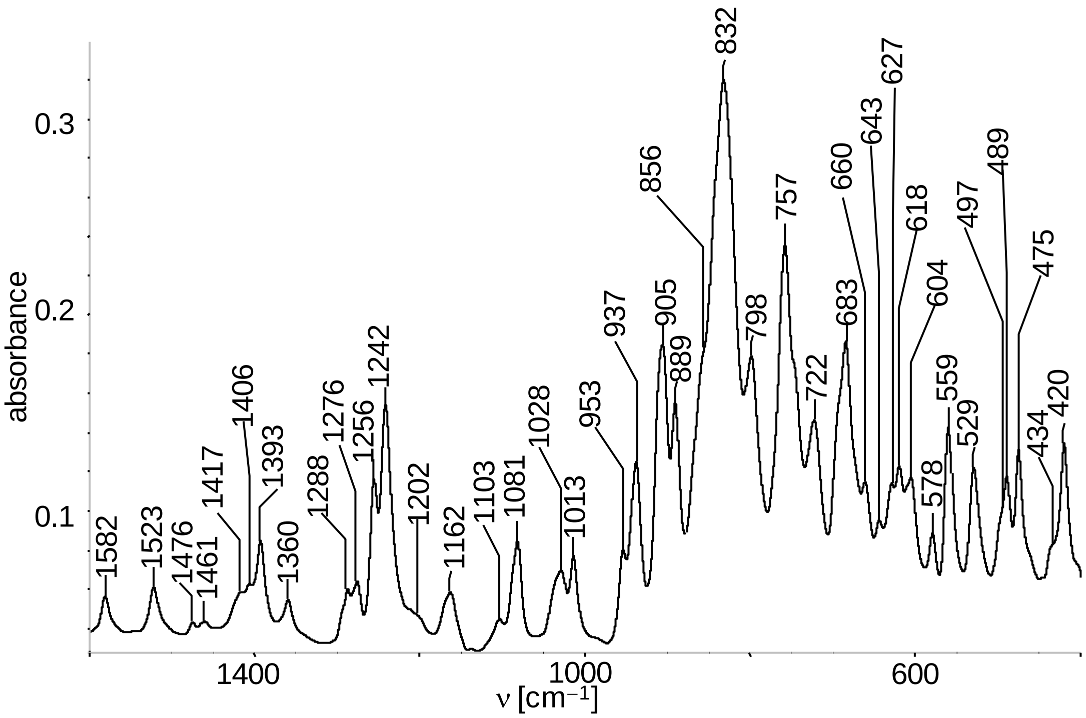

Figure S11. Expanded section (1600 – 400 cm^{-1}) of the solid-state FT-IR spectrum of compound **2-Si** shown in *Figure S10*.

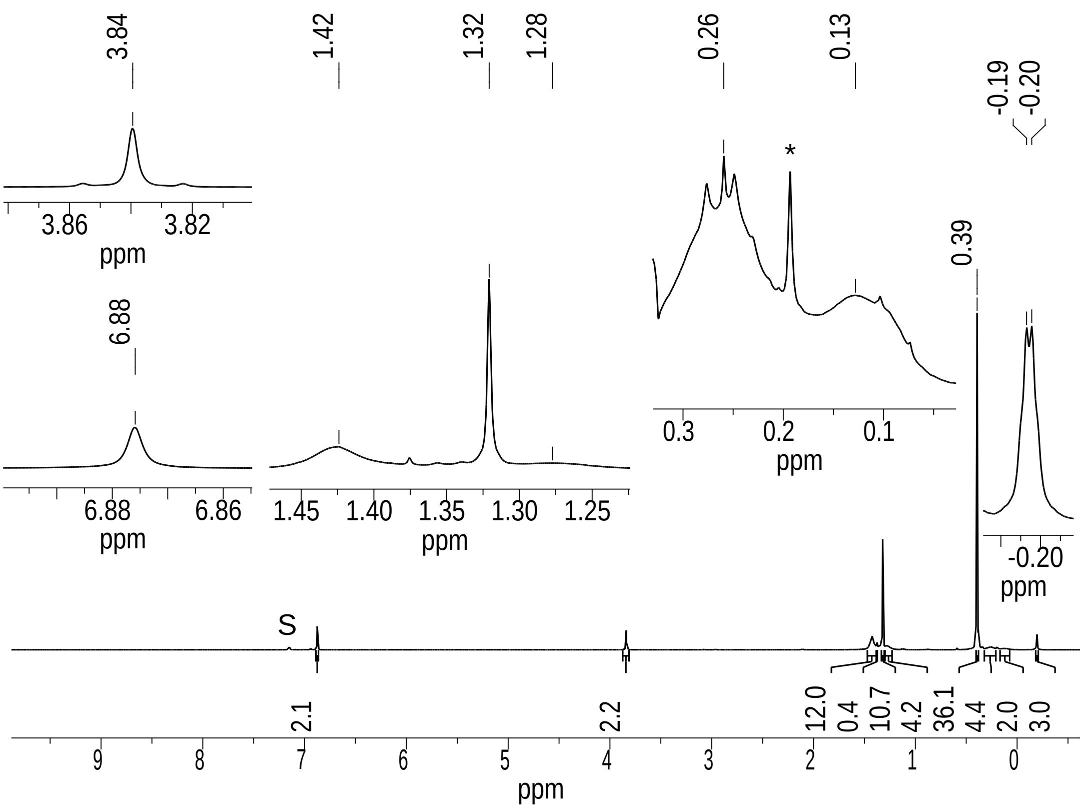

Figure S12. ^{1}H NMR spectrum (300.1 MHz) of compound **2-Si** in benzene-*d*$_6$ at 298 K; the character S marks the residual proton signal of the deuterated solvent. Enlarged excerpts of the spectrum are shown in the insets; the signal marked with an asterisk was identified as one of the ^{13}C satellites of the signal at δ = 0.39 ppm.

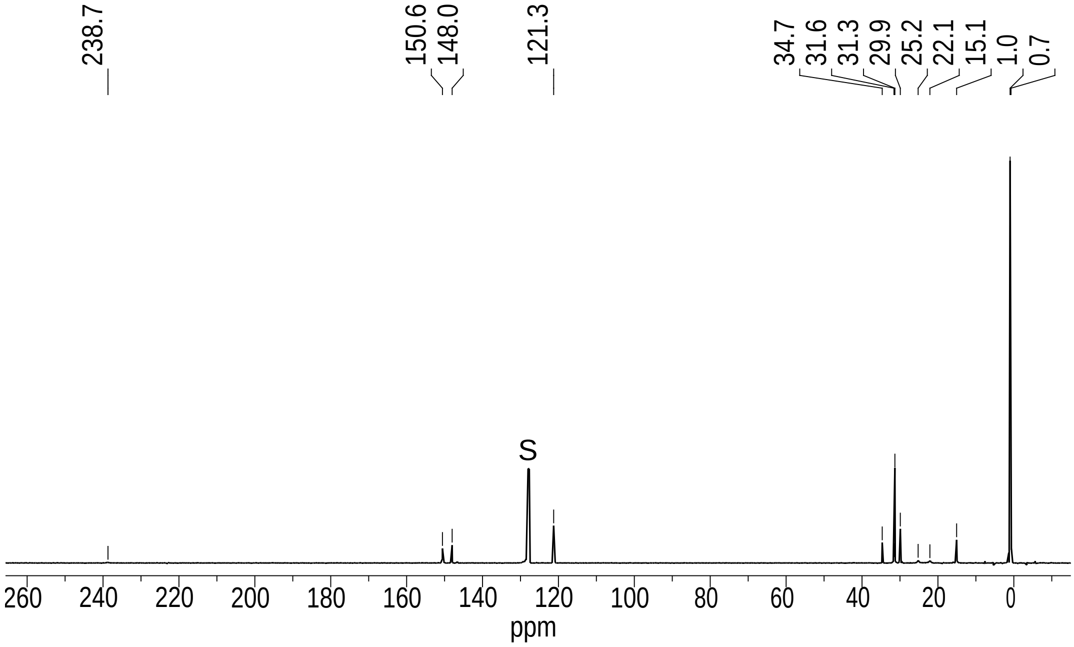

Figure S13. ^{13}C{^{1}H} NMR spectrum (75.47 MHz) of compound **2-Si** in benzene-*d*$_6$ at 298 K; the character S marks the ^{13}C signal of the deuterated solvent.

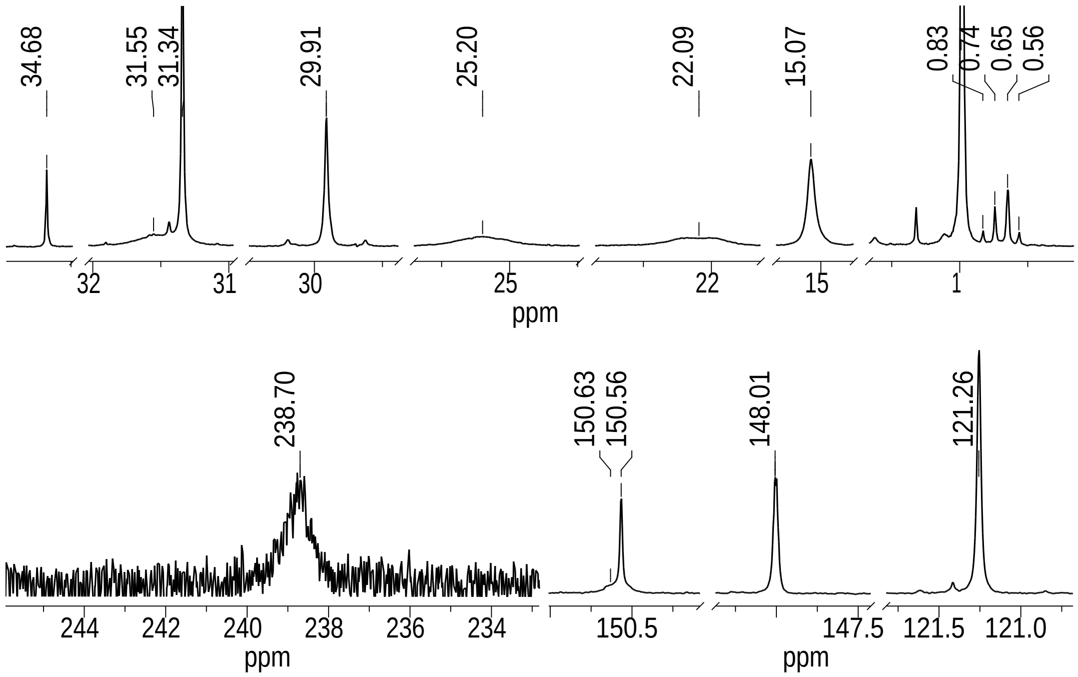

Figure S14. Expanded sections of the $^{13}C\{^{1}H\}$ NMR spectrum (75.47 MHz) of compound **2-Si** in benzene-d_6 at 298 K shown in *Figure S13*.

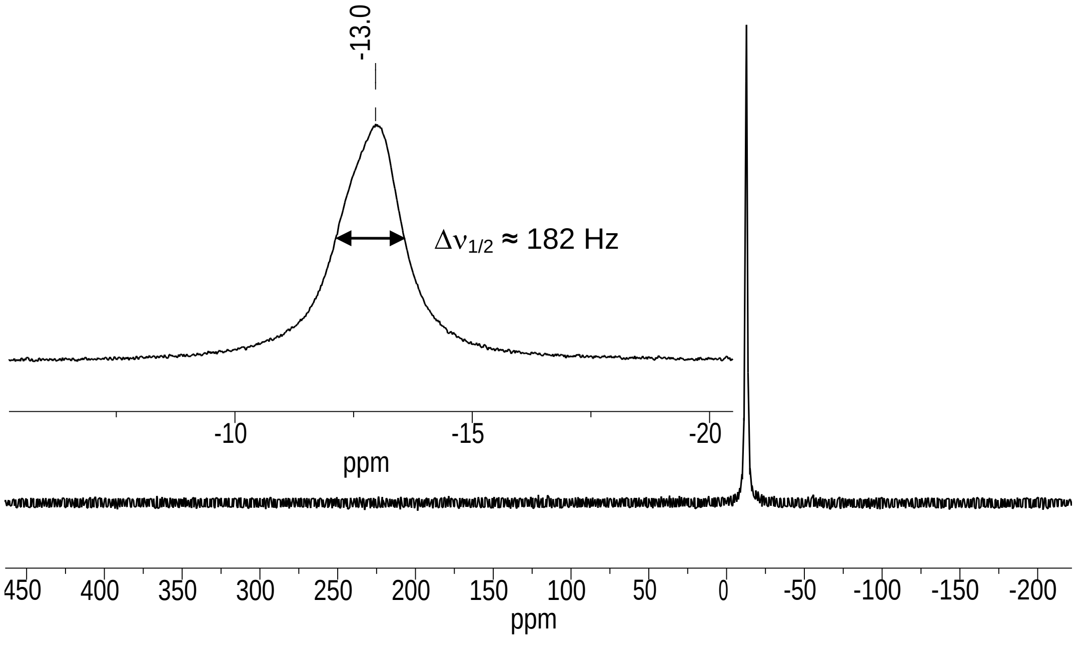

Figure S15. $^{31}P\{^{1}H\}$ NMR spectrum (121.5 MHz) of compound **2-Si** in benzene-d_6 at 298 K. An enlarged excerpt of the spectrum is shown in the inset.

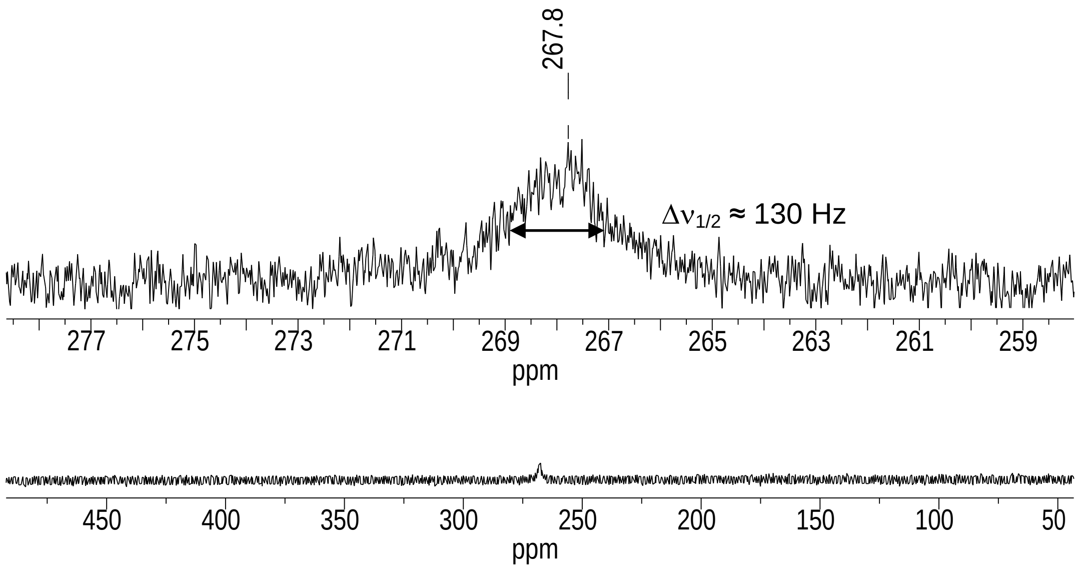

Figure S16. $^{29}Si\{^{1}H\}$ NMR spectrum (59.63 MHz) of compound **2-Si** in benzene-*d*$_6$ at 298 K from 50 to 500 ppm. The inset shows an enlarged excerpt of the spectrum.

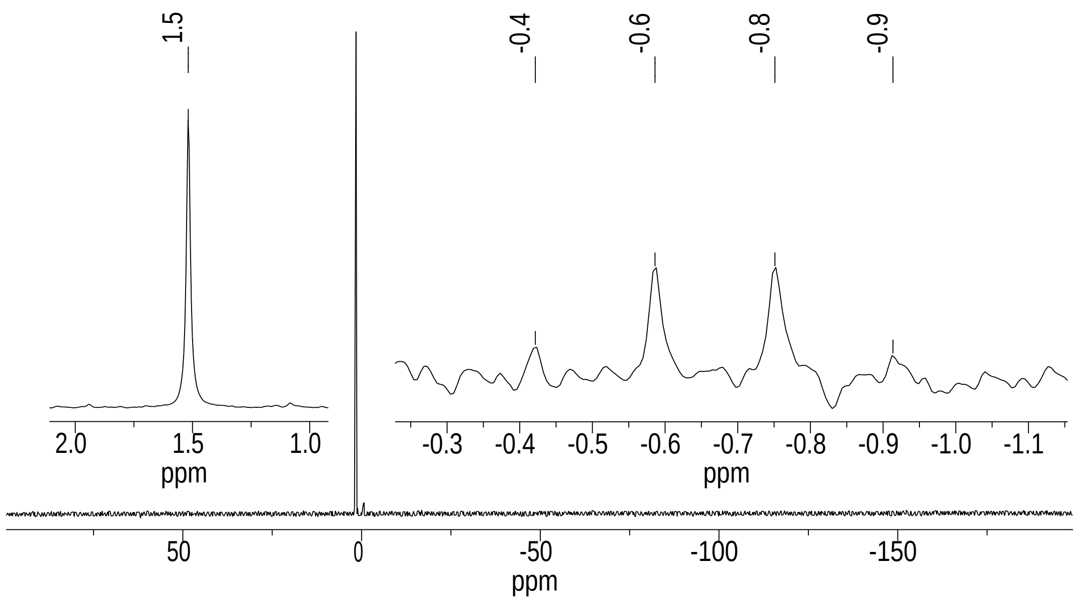

Figure S17. $^{29}Si\{^{1}H\}$ NMR spectrum (59.63 MHz) of compound **2-Si** in benzene-*d*$_6$ at 298 K from − 200 to 100 ppm. Enlarged excerpts are shown in the insets.

2.3 [(κ^3-tmps)(CO)$_2$Nb≡GeAr^{Mes}] (3-Ge)

30 mL of precooled toluene were added to a mixture of the orange complex **1** (500 mg, 0.91 mmol, 1 eq.) and the orange arylgermylene Ge(Ar^{Mes})Cl (385 mg, 0.91 mmol, 1 eq.) under stirring at – 40 °C, and the Schlenk tube was closed with a mercury bubbler. The

resulting orange-brown mixture was allowed to warm to room temperature within 1.5 h. The color of the suspension turned violet-brown at a temperature of – 35 °C under gas evolution. The reaction mixture was stirred for one hour at ambient temperature and then a FT-IR spectrum of the mixture was recorded to confirm the complete conversion of the starting material **1** to the germylidyne complex **3-Ge**. All volatiles were removed under reduced pressure, and the resulting violet-brown residue was extracted with a 1:1 mixture of toluene and petrol ether 40/60 (3 × 17 mL). The combined extracts were concentrated under reduced pressure to a volume of approximately 2 mL, upon which a brown, crystalline precipitate formed. The suspension was stored at – 30 °C for 14 h to complete the crystallization of complex **3-Ge**. The suspension was filtered at – 30 °C, and the dark brown crystals were washed with a 1:2 mixture of diethyl ether and *n*-pentane (3 × 5 mL) at room temperature. Drying of the solid under vacuum for one hour yielded the toluene-hemisolvate of **3-Ge** as dark violet crystals. The crystals were grinded and then suspended in 5 mL of *n*-pentane and stirred for 24 h to remove the toluene, which was accomplished after filtration of the pale violet *n*-pentane supernatant. The resulting deep magenta powder was dried under high vacuum for one hour to obtain 392 mg (0.49 mmol, 54 %) of the germylidyne complex **3-Ge**. The compound is moderately soluble in benzene and toluene, and shows a good solubility in THF. It decomposes without melting to a dark brown substance at 284 °C.

Elemental analysis calcd. (%) for $C_{36}H_{52}GeNbO_2P_3Si$ (803.32 g mol^{-1}): C 53.82, H 6.52; found: C 53.29, H 6.59.

IR (toluene, cm^{-1}, *Figure S18*): $\tilde{\nu}$ = 1868 (vs), 1805 (vs) (ν_{CO}).

IR (solid, cm^{-1}, *Figure S19 and Figure S20*): $\tilde{\nu}$ = 3027 (w), 2960 (w), 2907 (w), 2893 (w), 2870 (w), 1848 (s, ν_{CO}), 1785 (vs, ν_{CO}), 1610 (w), 1570 (vw), 1554 (vw), 1482 (vw), 1446 (w), 1417 (w), 1405 (w), 1375 (w), 1362 (w), 1290 (w), 1278 (m), 1250 (w), 1173 (vw), 1096 (w), 1081 (m), 1035 (w), 1014 (m), 936 (m), 906 (s), 868 (w), 846 (m), 831 (m), 797 (m), 756 (s), 735 (m), 726 (m), 710 (m), 695 (m), 682 (w), 673 (w), 621 (m), 579 (w), 554 (m), 516 (m), 487 (m), 473 (m), 427 (m), 405 (w).

^{1}H NMR (300.1 MHz, benzene-*d*$_6$, 298 K, ppm, *Figure S21*): δ = − 0.26 (q, ^{4}J(H,P) ≈ 0.8 Hz, 3H, Si*Me*), − 0.04 (d, ^{2}J(H,P) = 8.5 Hz, 2H, *trans*-C*H*$_2$PMe$_2$), 0.18 (br, $\Delta\nu_{1/2}$ ≈ 14 Hz, 4H, 2 × *cis*-C*H*$_A$*H*$_B$PMe$_A$Me$_B$), 1.10 (br, $\Delta\nu_{1/2}$ ≈ 9 Hz, 6H, 2 × *cis*-CH$_A$H$_B$P*Me*$_A$Me$_B$), 1.11 (d, ^{2}J(H,P) ≈ 4.1 Hz, 6H, *trans*-CH$_2$P*Me*$_2$), 1.21 (br, $\Delta\nu_{1/2}$ ≈ 10 Hz, 6H, 2 × *cis*-CH$_A$H$_B$PMe$_A$*Me*$_B$), 2.37 (s, 6H, 2 × C^{4}-*Me*, Mes), 2.42 (s, 12H, 2 × C^{2,6}-*Me*, Mes), 6.99 (d, ^{3}J(H,H) = 7.5 Hz, 2H, C^{3,5}-*H*, C$_6$H$_3$), 7.06 (br, $\Delta\nu_{1/2}$ ≈ 3 Hz, 4H, 2 × C^{3,5}-*H*, Mes), 7.21 (t, ^{3}J(H,H) = 7.5 Hz, 1H, C^{4}-*H*, C$_6$H$_3$).

^{13}C{^{1}H} NMR (100.6 MHz, THF-*d*$_8$, 298 K, ppm, *Figure S22 and Figure S23*): δ = 0.6 (q, ^{3}J(C,P) = 6.7 Hz, 1C, Si*Me*), 15.3 (br, $\Delta\nu_{1/2}$ ≈ 9 Hz, 1C, *trans*-*C*H$_2$PMe$_2$), 15.7 (s, 2C, 2 × *cis*-*C*H$_A$H$_B$PMe$_A$Me$_B$), 21.3 (s, 4C, 2 × C^{2,6}-*Me*, Mes), 21.4 (s, 2C, 2 × C^{4}-*Me*, Mes), 21.8 (d,

^{1}J(C,P) ≈ 17.5 Hz, 2C, *trans*-CH$_2$P*Me$_2$*), 24.8 – 25.0 (m, 2C, 2 × *cis*-CH$_A$H$_B$PMe$_A$*Me$_B$*, signal overlaps with the ^{13}C NMR signal of the solvent), 33.0 (q, ^{n}J(C,P) = 7.6 Hz, 2C, 2 × *cis*-CH$_A$H$_B$P*Me$_A$*Me$_B$), 127.8 (s, 2C, *C^{3,5}*-H, C$_6$H$_3$), 128.5 (s, 1C, *C^4*-H, C$_6$H$_3$), 129.1 (s, 4C, 2 × *C^{3,5}*-H, Mes), 136.1 (s, 2C, 2 × *C^4*, Mes), 137.1 (s, 4C, 2 × *C^{2,6}*, Mes), 140.0 (s, 2C, 2 × *C^1*, Mes), 145.0 (s, 2C, *C^{2,6}*, C$_6$H$_3$), 166.6 (m, 1C, *C^1*, C$_6$H$_3$), 239.2 (br, $\Delta\nu_{1/2}$ ≈ 79 Hz, 2C, 2 × *C*O).

^{31}P{^{1}H} NMR (121.5 MHz, benzene-*d$_6$*, 298 K, ppm, *Figure S24*): δ = −11.0 (br, $\Delta\nu_{1/2}$ ≈ 187 Hz, 3P, 2 × *cis*-CH$_A$H$_B$*P*Me$_A$Me$_B$ + *trans*-CH$_2$*P*Me$_2$).

^{31}P{^{1}H} NMR (121.5 MHz, THF-*d$_8$*, 283 K, ppm, *Figure S25*): δ = −10.7 (br, $\Delta\nu_{1/2}$ ≈ 153 Hz, 3P, 2 × *cis*-CH$_A$H$_B$*P*Me$_A$Me$_B$ + *trans*-CH$_2$*P*Me$_2$).

^{31}P{^{1}H} NMR (121.5 MHz, THF-*d$_8$*, 193 K, ppm, *Figure S26*): δ = −10.4 (d, ^{2}J(P,P) = 20.9 Hz 2P, 2 × *cis*-CH$_A$H$_B$*P*Me$_A$Me$_B$), −9.6 (t, ^{2}J(P,P) = 20.9 Hz 1P, *trans*-CH$_2$*P*Me$_2$).

^{29}Si{^{1}H} NMR (79.49 MHz, THF-*d$_8$*, 298 K, ppm, *Figure S27*): δ = 0.6 (q, ^{2}J(P,Si) = 9.8 Hz, 1Si, *Si*Me).

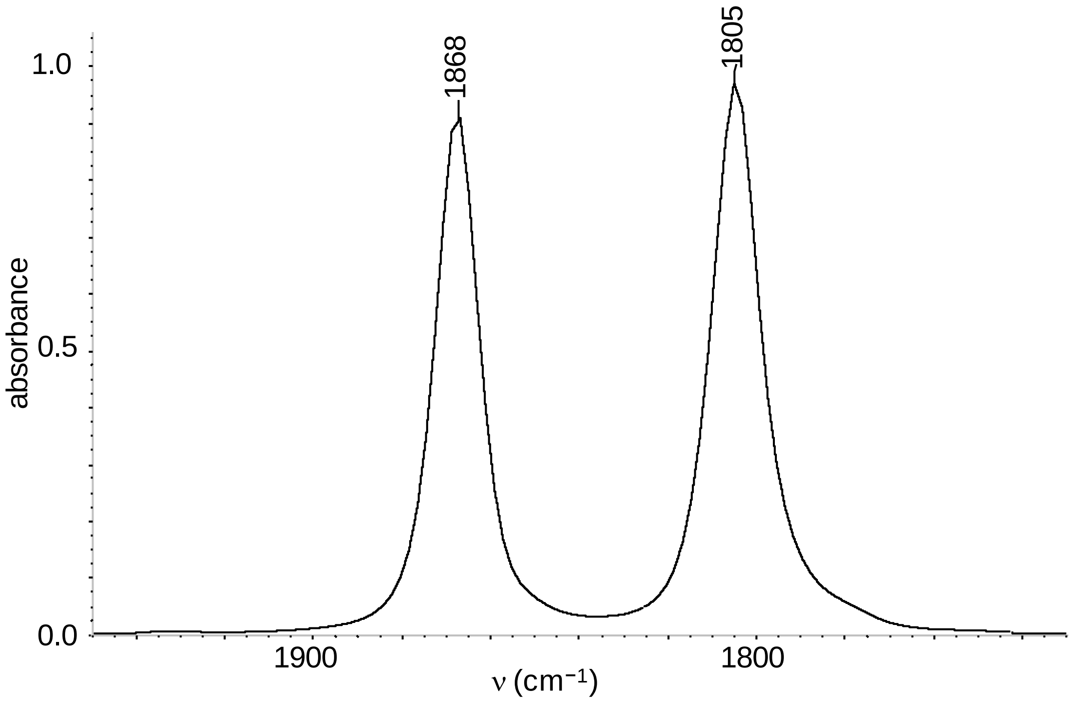

Figure S18. FT-IR spectrum of **3-Ge** in toluene in the range of 1950 – 1730 cm^{-1}.

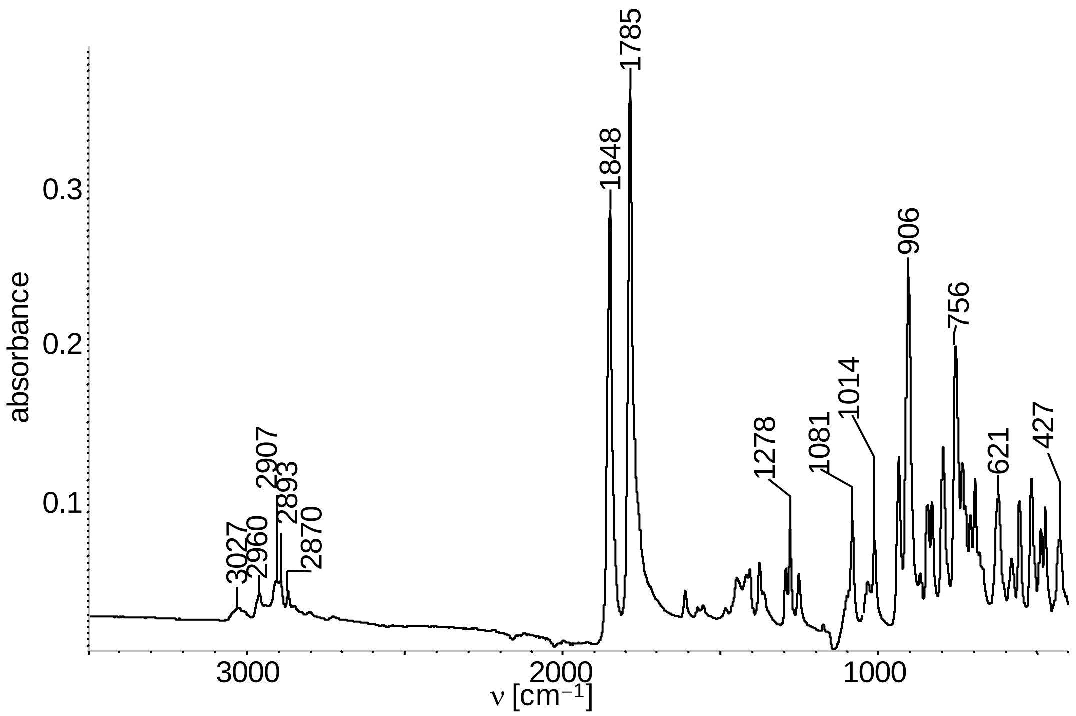

Figure S19. Solid state FT-IR spectrum of **3-Ge** in the range of 3500 – 400 cm^{-1}.

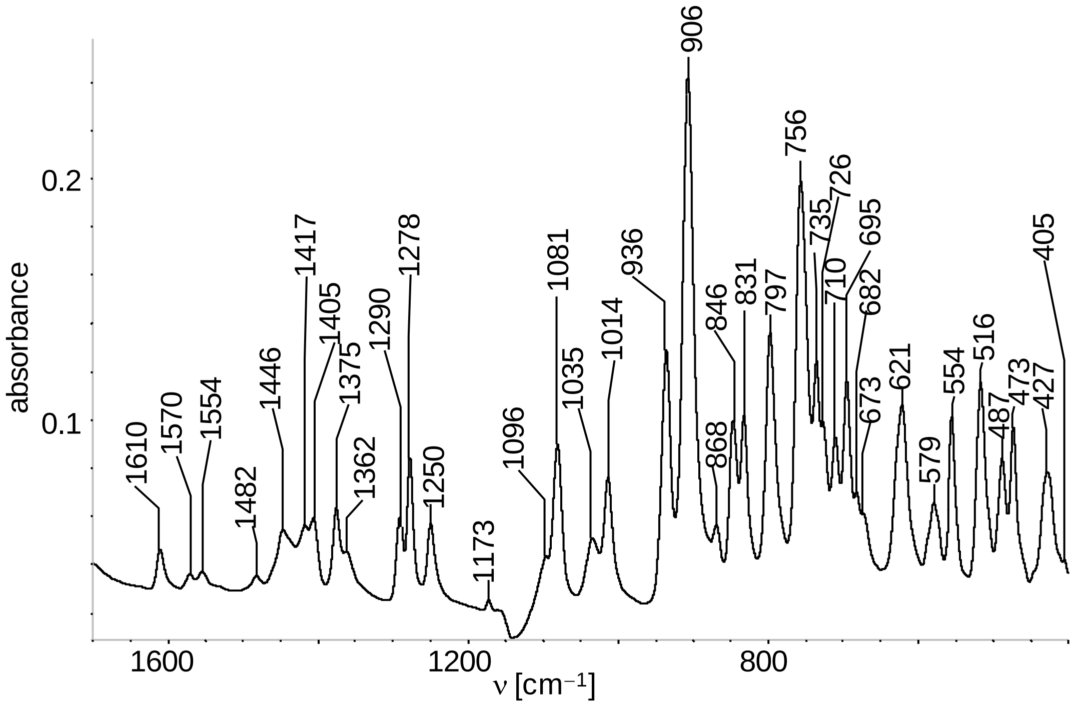

Figure S20. Expanded section (1700 – 400 cm^{-1}) of the solid-state FT-IR spectrum of **3-Ge** shown in *Figure S19*.

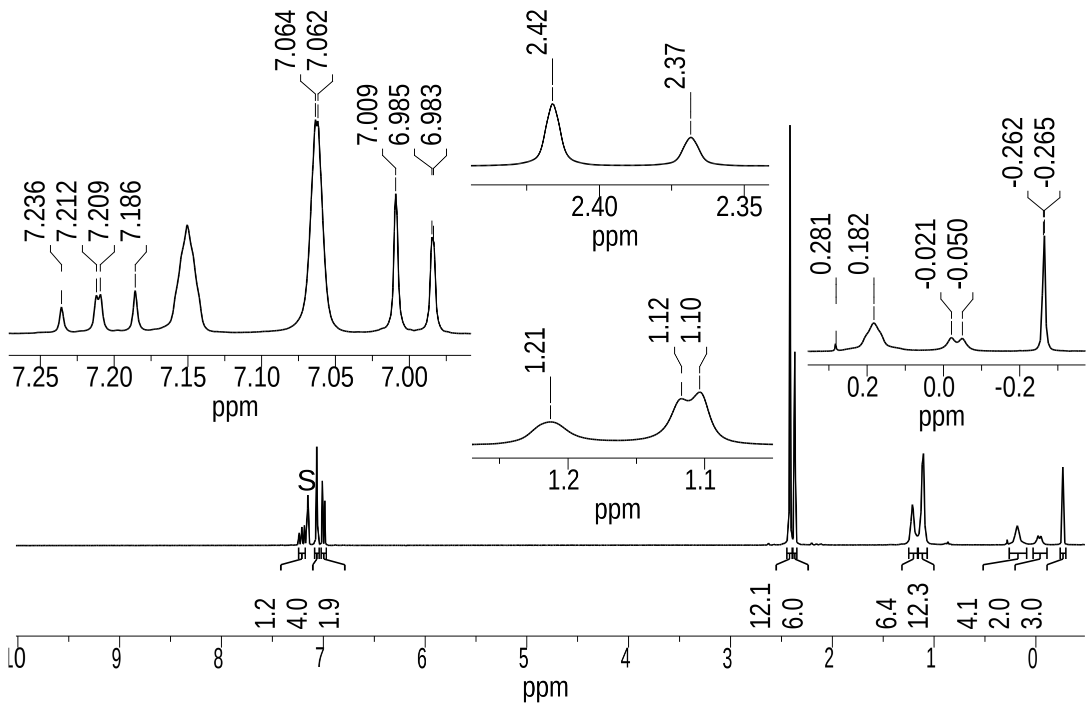

Figure S21. ^{1}H NMR spectrum (300.1 MHz) of **3-Ge** in benzene-*d*$_6$ at 298 K; the residual proton signal of the deuterated solvent is marked with the character S. Enlarged excerpts of the spectrum are shown in the insets.

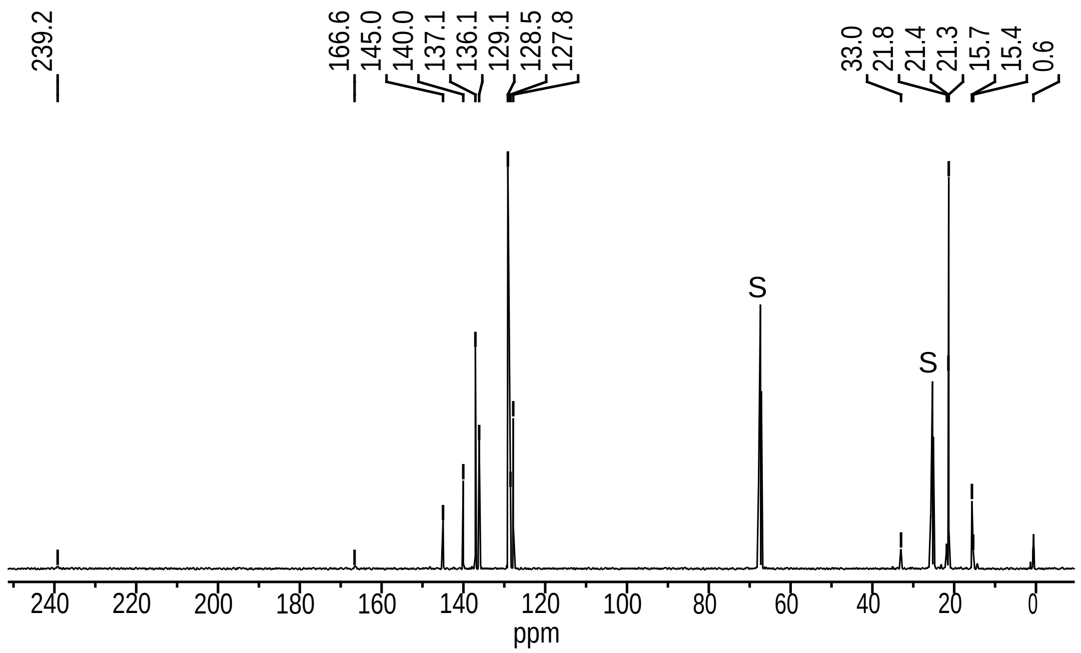

Figure S22. ^{13}C{^{1}H} NMR spectrum (100.6 MHz) of compound **3-Ge** in THF-*d*$_8$ at 298 K; the character S marks the ^{13}C signals of the deuterated solvent.

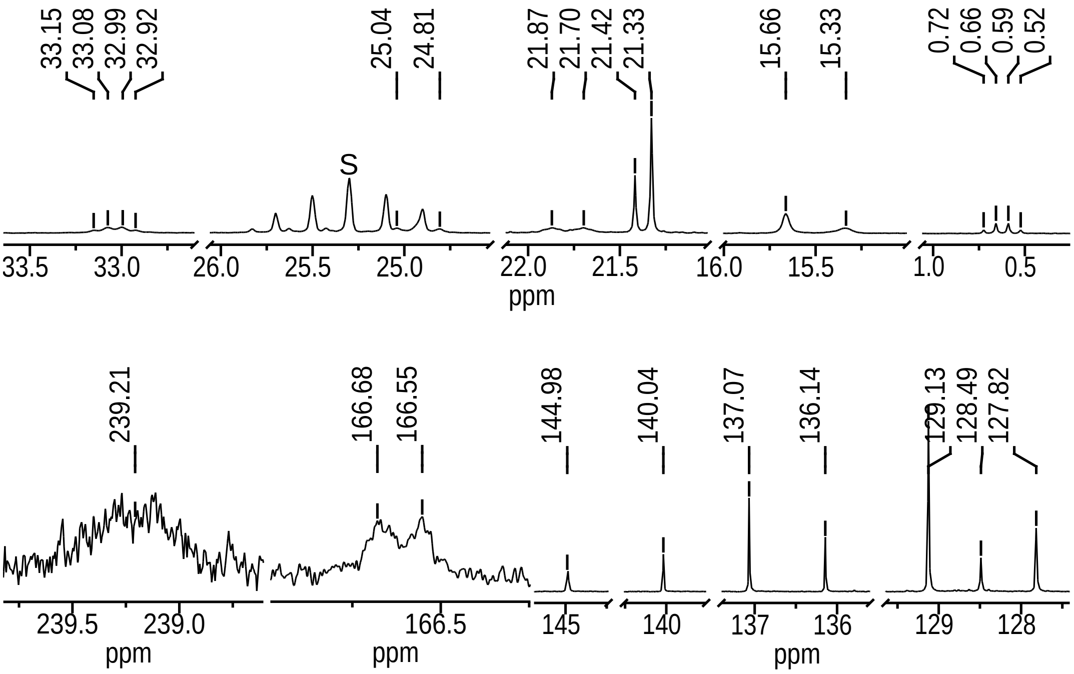

Figure S23. Expanded sections of the $^{13}C\{^{1}H\}$ NMR spectrum (100.6 MHz) of compound **3-Ge** in THF-d_8 at 298 K shown in *Figure S22*; the character S marks the ^{13}C signal of the deuterated solvent.

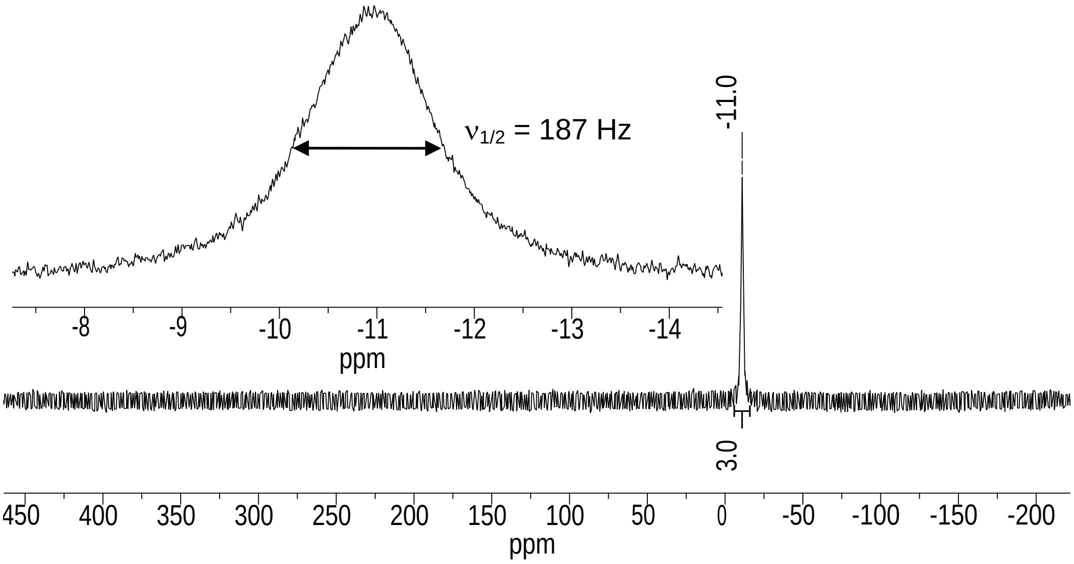

Figure S24. $^{31}P\{^{1}H\}$ NMR spectrum (121.5 MHz) of compound **3-Ge** in benzene-d_6 at 298 K. An enlarged excerpt of the spectrum is shown in the inset.

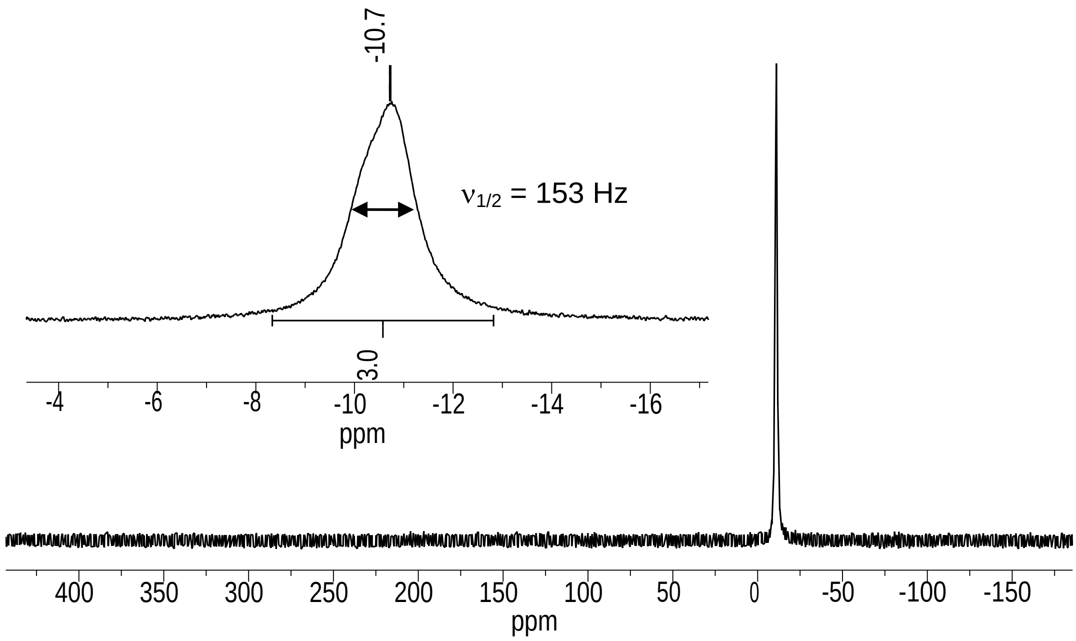

Figure S25. ^{31}P{^{1}H} NMR spectrum (121.5 MHz) of compound **3-Ge** in THF-d_8 at 283 K. An enlarged excerpt of the spectrum is shown in the inset.

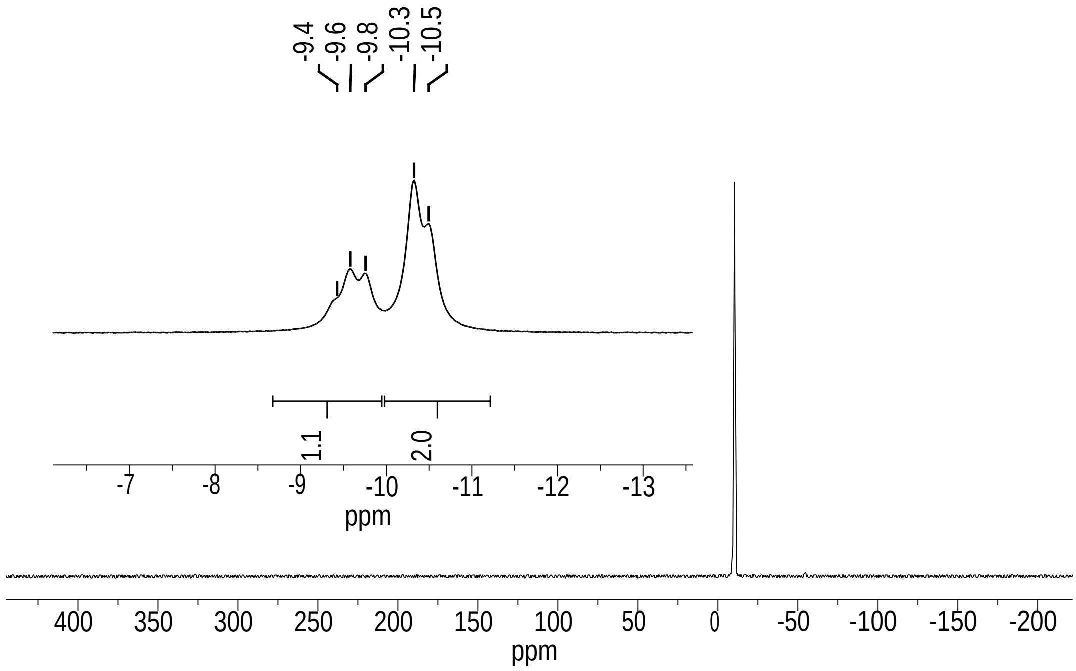

Figure S26. ^{31}P{^{1}H} NMR spectrum (121.5 MHz) of compound **3-Ge** in THF-d_8 at 193 K. An enlarged excerpt of the spectrum is shown in the inset.

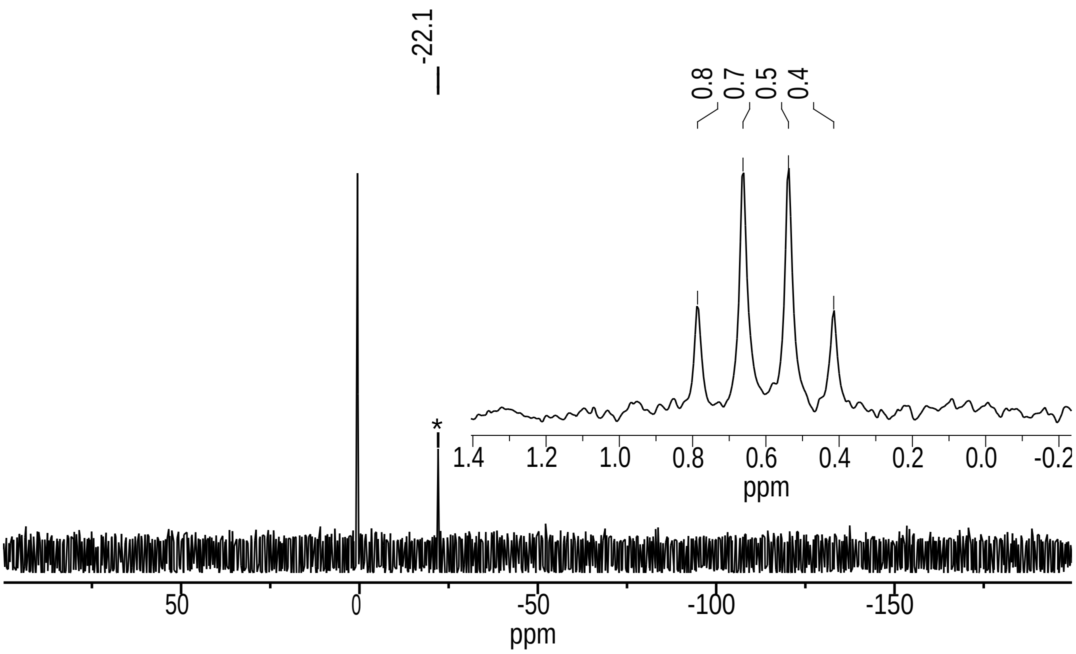

Figure S27. ^{29}Si{^{1}H} NMR spectrum (79.49 MHz) of compound **3-Ge** in THF-d_8 at 298 K. An enlarged excerpt is shown in the inset; the signal marked with an asterisk originates from oligomeric siloxanes coming from the polymer syringe used for the sample preparation.

2.4 [(κ^3-tmps)(CO)$_2$Nb≡SnAr^{Mes}] (3-Sn)

836 mg (1.53 mmol) of the orange complex **1** and 714 mg (1.53 mmol, 1 eq.) of the yellow arylstannylene Sn(Ar^{Mes})Cl **2-Sn** were premixed in a Schlenk tube and 80 mL of precooled toluene were added to the solids under stirring at − 78 °C. The Schlenk tube was closed with a mercury bubbler, and the orange suspension was warmed up to ambient temperature within 4 h. During this time the color of the solution changed to red-brown and gas evolution was observed. A FT-IR spectrum of the solution was recorded, which revealed the complete consumption of the starting material **1** and the formation of the metallostannylene [(κ^3-tmps)(CO)$_3$Nb–SnAr^{Mes}] (**4-Sn**)[S12] as the main product along with a small amount of the stannylidyne complex **3-Sn**. The mercury bubbler was then replaced by a Schlenk cap, and the mixture was heated to 80 °C under static vacuum for 1.5 h. Every 10 to 15 minutes

[S12] Several attempts were undertaken to obtain compound **4-Sn** in pure form, free from **3-Sn.** The attempts failed due to the extreme sensivity of **4-Sn** towards air and light and its partial conversion to **3-Sn** during purification. However, compound **4-Sn** was characterized by IR, ^{1}H and ^{31}P NMR spectroscopy. IR (toluene, cm^{-1}): $\tilde{\nu}$ = 1887 (m), 1818 (vs), 1796 (s) (ν_{CO}). ^{1}H NMR (300.1 MHz, benzene-d_6, 298 K, ppm): δ = −0.29 (s, 3H, Si*Me*), 0.02 – 0.05 (m, 6H, 2 × CH_AH$_B$PMe$_A$Me$_B$, 2 × CH$_A$$H_BPMe_AMe_B$, and C$H_2PMe_2$), 1.08 – 1.10 (m, 18H, 2 × CH$_A$H$_B$P$Me_A$$Me_B$ and *trans*-CH$_2$PMe_2), 2.25 (s, 6H, 2 × C^4-*Me*, Mes), 2.65 (s, 12H, 2 × C^{2,6}-*Me*, Mes), 6.88 (s, 4H, 2 × C^{3,5}-*H*, Mes), 7.31 (d, ^3J(H,H) = 7.5 Hz, C^{3,5}-*H*, C$_6$H$_3$), 7.50 (t, ^3J(H,H) = 7.5 Hz, 1H, C^4-*H*, C$_6$H$_3$). ^{31}P{^{1}H} NMR (121.5 MHz, benzene-d_6, 298 K, ppm): δ = −18.6 (br, $\Delta\nu_{1/2}$ ≈ 151 Hz, 3P, 2 × CH$_A$H$_B$*P*Me$_A$Me$_B$ and CH$_2$*P*Me$_2$).

vacuum was shortly applied to the system to remove the released carbon monoxide. At the end of the heating period the color of the solution had changed to deep violet and the metallostannylene **4-Sn** had been almost completely converted to the stannylidyne complex **3-Sn**, which was confirmed by an IR spectrum of the deep violet solution. The solvent was then removed at 60 °C under reduced pressure and the obtained violet residue was extracted with a 1:1 mixture of petrol ether 40/60 and toluene (30 + 20 + 25 + 25 mL). The violet extracts were combined and concentrated *in vacuo* to approximately 1 mL leading to the formation of dark crystals of **3-Sn**. The suspension was stored at − 30 °C over night to complete the crystallization of **3-Sn**. The crystals were separated from the violet solution by filtration, washed with *n*-pentane (3 × 2 mL) and finally dried under vacuum for one hour to afford 1029 mg of crude **3-Sn**, which contained according to IR and NMR spectroscopy 3 mol% of the metallostannylene **4-Sn**. The crude product was recrystallized from 2 mL of toluene at − 30 °C. The obtained dark crystals were redissolved in 20 mL of toluene and 30 mL of *n*-hexane were added slowly. The precipitated solid was filtered off and discarded, The violet supernatant solution was evaporated to dryness *in vacuo*, and the obtained dark violet solid was suspended and stirred in 10 mL of *n*-hexane over night to dissolve the toluene cocrystallized with the solid. The pale violet supernatant was filtered off, and the bright violet solid was washed with a 2:1 toluene petrolether (40/60) mixture (1 + 2 mL) and dried under high vacuum for one hour to give 910 mg (1.07 mmol, 70 %) of **3-Sn** as a violet powder. The product still contained a tiny amount of the metallostannylene **4-Sn** according to NMR spectroscopy (cf. *Figure S34*).

Compound **3-Sn** is sparingly soluble in *n*-hexane, moderately soluble in benzene and toluene, but shows a good solubility in THF. It decomposes without melting at 266 °C to a black mass, which melts into a brown oil upon further heating to 271 °C.

Elemental analysis calcd. (%) for $C_{36}H_{52}NbO_2P_3SiSn$ (849.42 g mol^{-1}): C 50.90, H 6.17; found: C 50.55, H 6.17.

IR (toluene, cm^{-1}, *Figure S28*): $\tilde{\nu}$ = 1851 (vs), 1791 (vs) (ν_{CO}).

IR (solid, cm^{-1}, *Figure S29* and *Figure* S30): $\tilde{\nu}$ = 3026 (w), 2960 (w), 2894 (w), 2870 (w), 1831 (s, ν_{CO}), 1771 (vs ν_{CO}), 1610 (w), 1569 (w), 1556 (w), 1479 (w), 1445 (w), 1416 (w), 1405 (w), 1375 (w), 1361 (w), 1290 (w), 1277 (m), 1250 (w), 1172 (vw), 1160 (vw), 1099 (w), 1080 (m), 1034 (w), 1012 (m), 935 (m), 904 (s), 867 (w), 846 (m), 831 (m), 796 (m), 753 (s), 732 (m), 709 (m), 693 (m), 680 (m), 621 (m), 579 (w), 570 (w), 553 (m), 516 (m), 485 (w), 475 (m), 427 (w).

^{1}H NMR (300.1 MHz, THF-d_8, 298 K, ppm, *Figure S31*): δ = 0.04 (q, ^4J(H,P) ≈ 0.8 Hz, 3H, Si*Me*), 0.74 (d, ^2J(H,P) = 7.9 Hz, 6H, 2 × *cis*-CH_AH$_B$PMe$_A$Me$_B$, 2 × *cis*-CH$_A$$H_BPMe_AMe_B$, *trans*-C$H_2PMe_2$), 1.15 (br, $\Delta\nu_{1/2}$ ≈ 14 Hz, 6H, 2 × *cis*-CH$_A$H$_B$PMe_AMe$_B$), 1.39 (br, $\Delta\nu_{1/2}$ ≈ 14 Hz, 12H, 2 × *cis*-CH$_A$H$_B$PMe$_A$$Me_B$, *trans*-CH$_2PMe_2$), 2.09 (s, 12H, 2 × C^{2,6}-*Me*, Mes),

2.27 (s, 6H, 2 × C^4-*Me*, Mes), 6.88 (s, 4H, 2 × $C^{3,5}$-*H*, Mes), 7.03 (d, ^3J(H,H) = 7.5 Hz, $C^{3,5}$-*H*, C_6H_3), 7.31 (t, ^3J(H,H) = 7.5 Hz, 1H, C^4-*H*, C_6H_3).

$^{13}C\{^1H\}$ NMR (75.47 MHz, THF-d_8, 298 K, ppm, *Figure S32* and *Figure* S33): δ = 0.6 (q, ^3J(C,P) = 6.8 Hz, 1C, Si*Me*), 16.1 (br, $\nu_{1/2}$ ≈ 7 Hz, 2C, 2 × *cis*-*C*H_AH_BP$Me_A$$Me_B$), 16.9 (br, $\nu_{1/2}$ ≈ 13 Hz, 1C, *trans*-*C*H_2PMe_2), 21.1 (s, 4C, 2 × $C^{2,6}$-*Me*, Mes), 21.4 (s, 2C, 2 × C^4-*Me*, Mes), 22.0 (d, ^1J(C,P) ≈ 18.1 Hz, 2C, *trans*-CH_2P*Me*$_2$), 24.7 – 25.8 (m, 2C, 2 × *cis*-CH_AH_BPMe_A*Me*$_B$, the signal overlaps with the ^{13}C NMR signal of the solvent), 35.2 (br, $\Delta\nu_{1/2}$ ≈ 25 Hz, 2C, 2 × *cis*-CH_AH_BP*Me*$_A$$Me_B$), 128.3 (s, 3C, $C^{3,5}$-H + C^4-H, C_6H_3), 129.3 (s, 4C, 2 × $C^{3,5}$-H, Mes), 136.5 (s, 2C, 2 × C^4, Mes), 136.9 (s, 4C, 2 × $C^{2,6}$, Mes), 140.6 (s, 2C, 2 × C^1, Mes), 146.0 (s, 2C, $C^{2,6}$, C_6H_3), 184.4 (br, $\nu_{1/2}$ ≈ 15 Hz, 1C, C^1, C_6H_3), 238.9 (br, $\Delta\nu_{1/2}$ ≈ 63 Hz, 2C, 2 × *C*O).

$^{31}P\{^1H\}$ NMR (121.5 MHz, THF-d_8, 298 K, ppm, *Figure S34*): δ = − 11.1 (br, $\Delta\nu_{1/2}$ ≈ 112 Hz, 2P, 2 × *cis*-CH_AH_B*P*$Me_A$$Me_B$), − 3.0 (br, $\Delta\nu_{1/2}$ ≈ 141 Hz, 1P, *trans*-CH_2*P*Me_2).

$^{29}Si\{^1H\}$ NMR (59.63 MHz, THF-d_8, 298 K, ppm, *Figure S35*): δ = 0.3 (q, ^2J(P,Si) = 9.7 Hz, 1Si, *Si*Me).

$^{119}Sn\{^1H\}$ NMR (111.9 MHz, THF-d_8, 243 K, ppm, *Figure S36*): δ = 829.7 (br, $\Delta\nu_{1/2}$ ≈ 1297 Hz, 1Sn, Nb≡*Sn*).

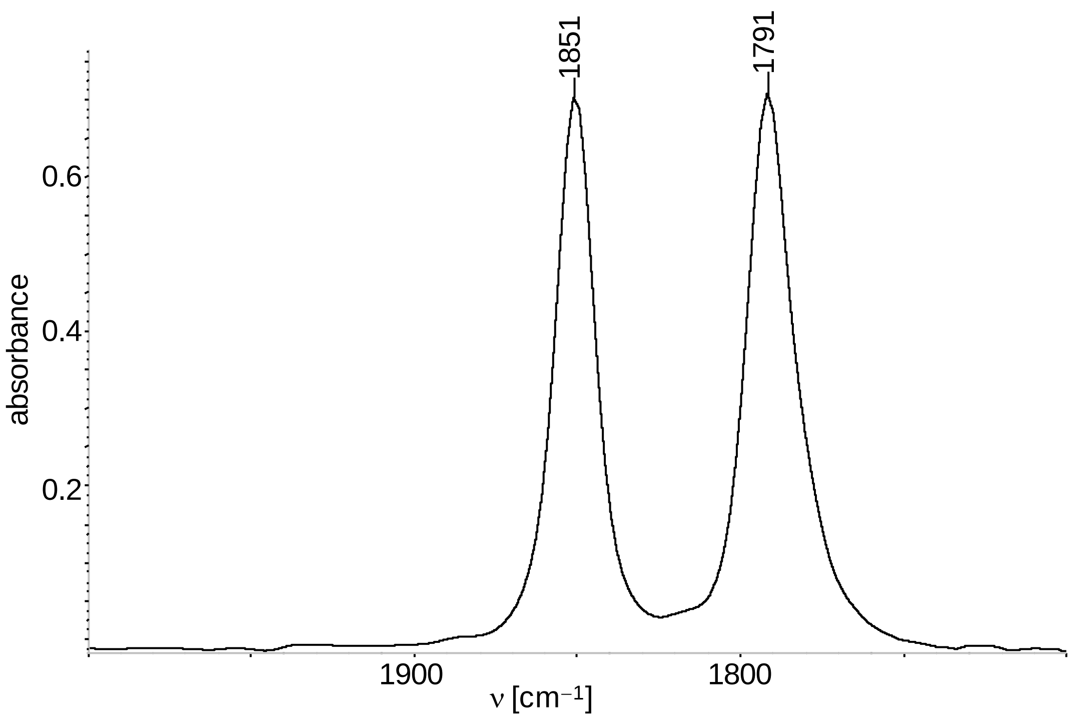

Figure S28. FT-IR spectrum of **3-Sn** in toluene in the range of 2000 – 1700 cm^{-1}.

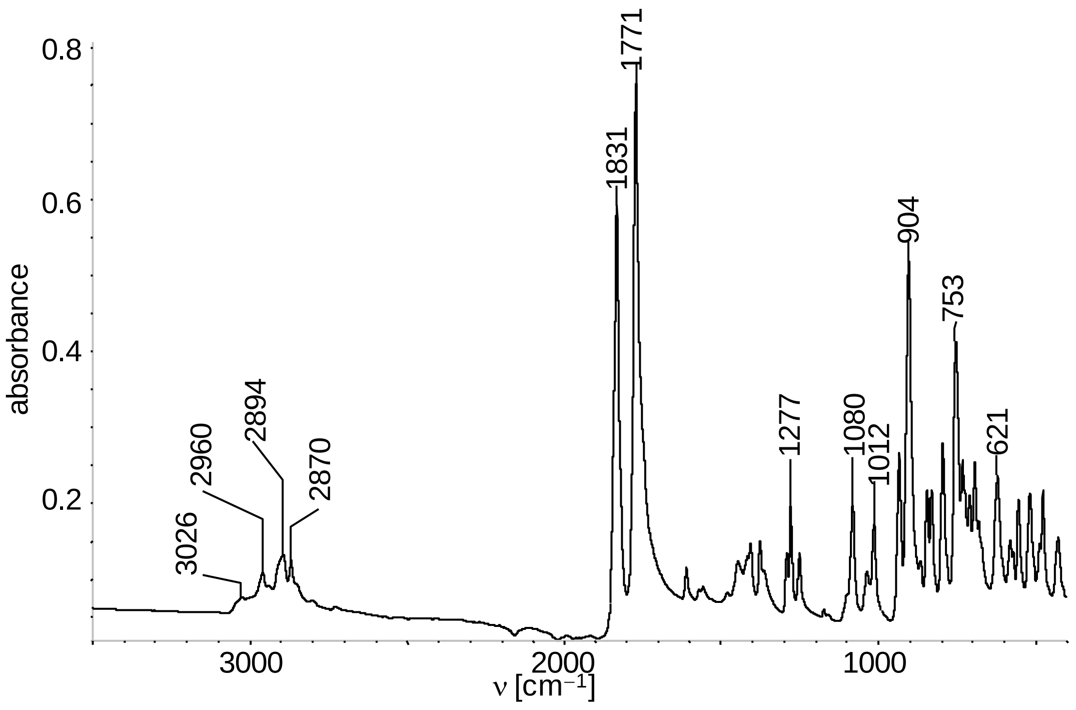

Figure S29. Solid state FT-IR spectrum of **3-Sn** in the range of 3500 – 400 cm^{-1}.

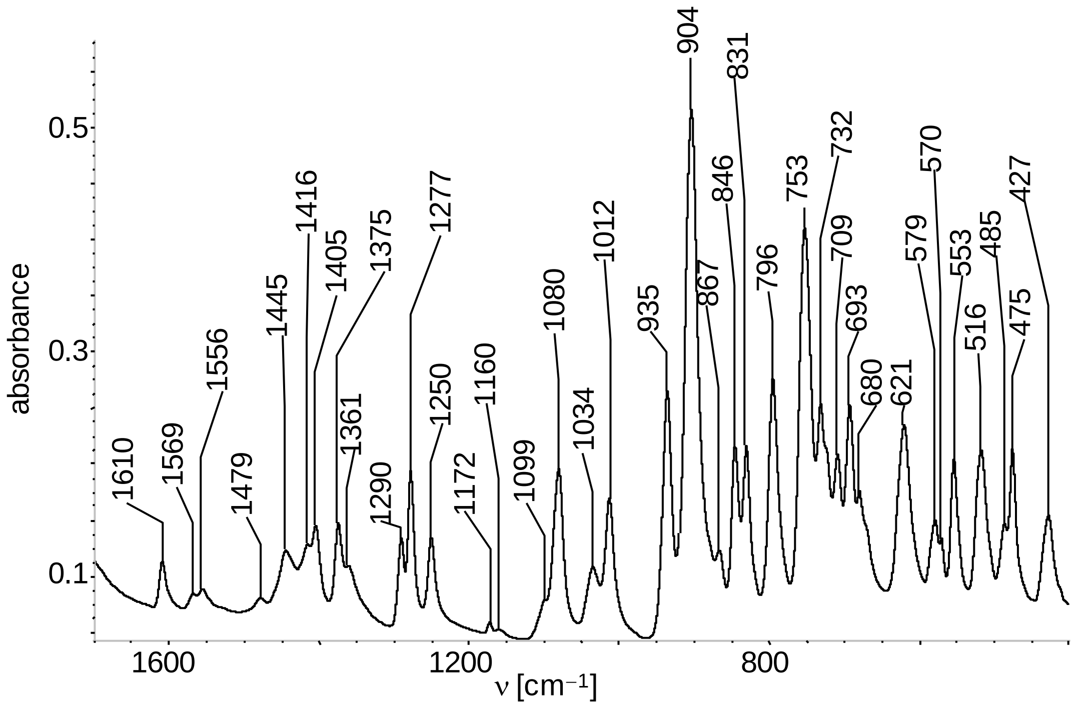

Figure S30. Expanded section (1500 – 400 cm^{-1}) of the solid-state FT-IR spectrum of **3-Sn**.

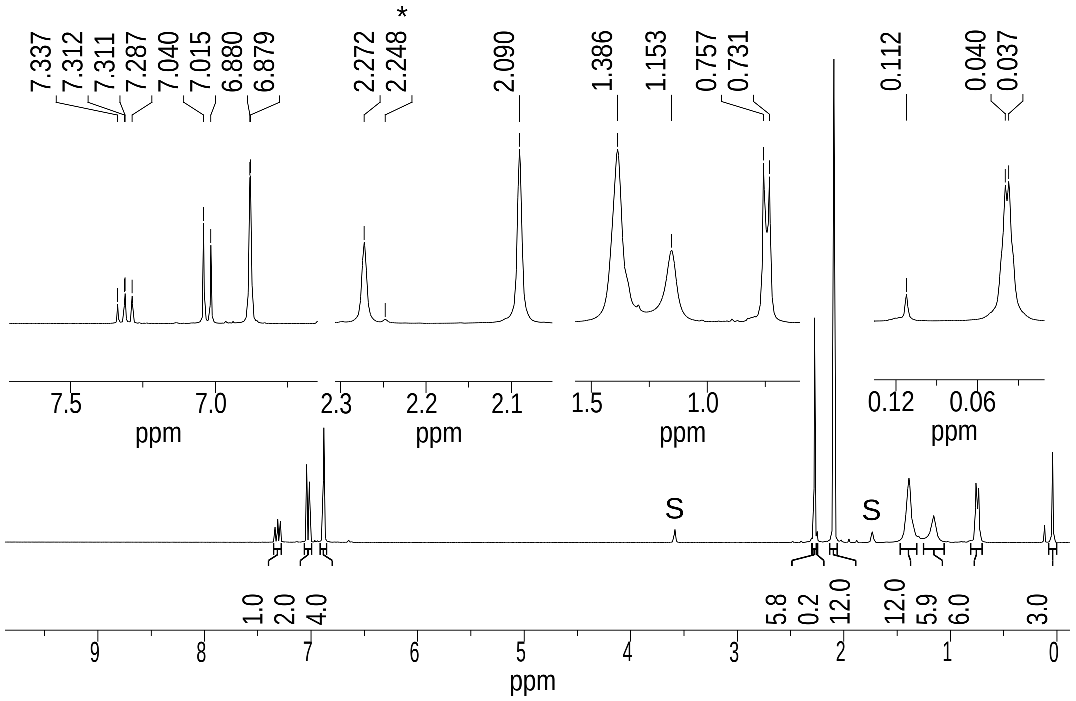

Figure S31. ^{1}H NMR spectrum (300.1 MHz) of **3-Sn** in THF-d_8 at 298 K; the residual proton signals of the deuterated solvent are marked with the character S. Enlarged excerpts of the spectrum are shown in the insets; the signal noted with an asterisk arises from traces of the metallostannylene [(κ^3-tmps)(CO)$_3$Nb–SnAr^{Mes}] (**4-Sn**).

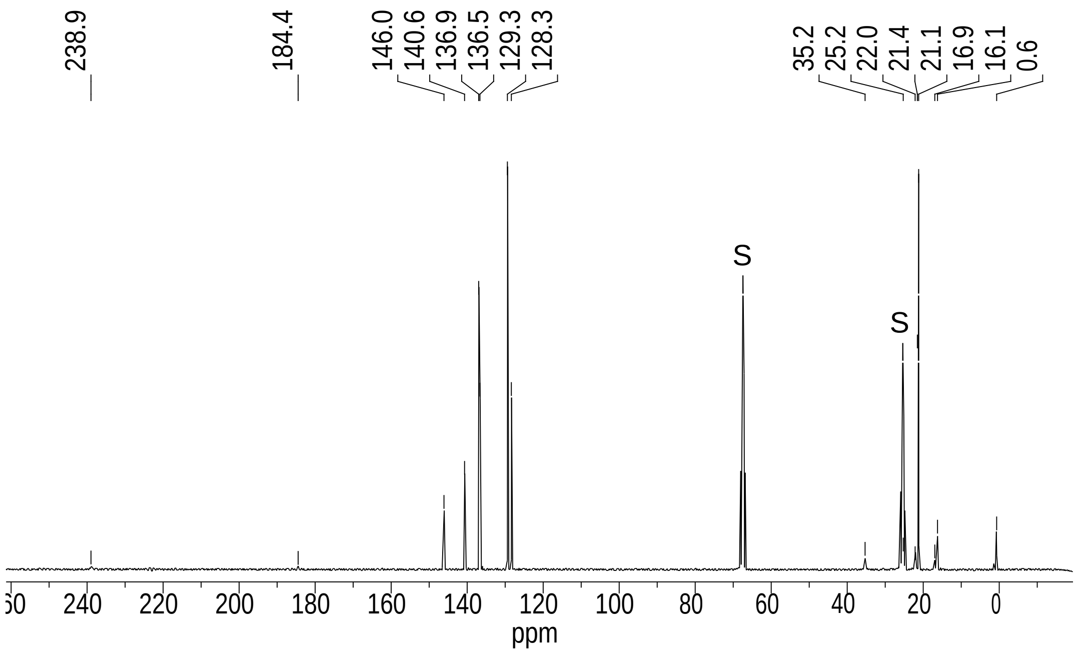

Figure S32. ^{13}C{^{1}H} NMR spectrum (75.47 MHz) of **3-Sn** in THF-d_8 at 298 K; the character S marks the ^{13}C signals of the deuterated solvent.

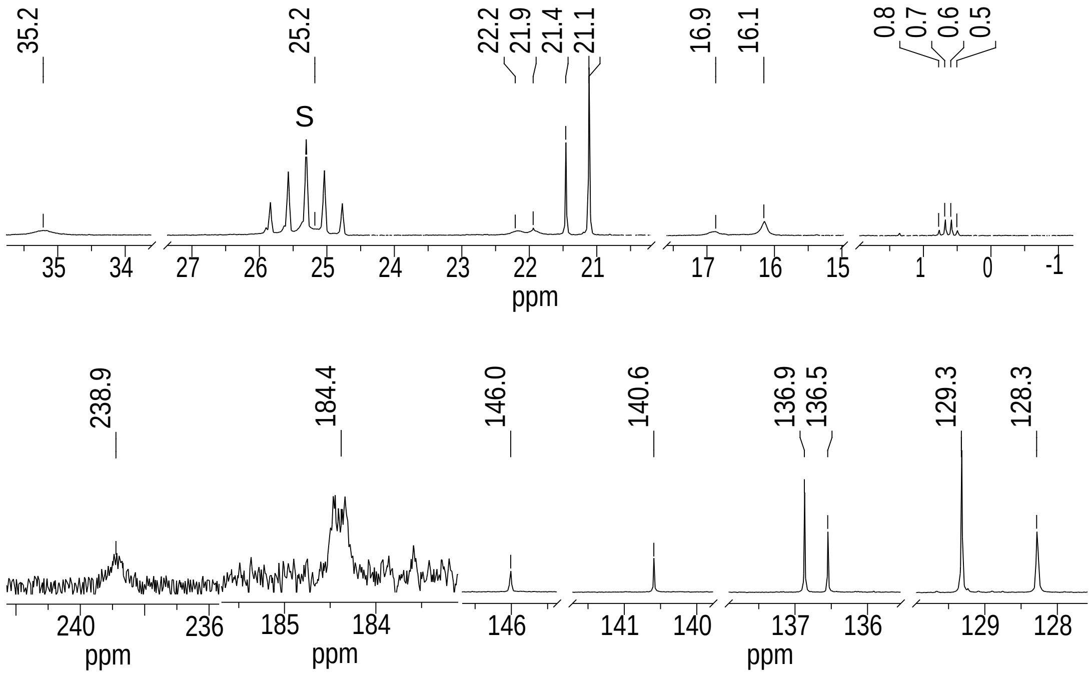

Figure S33. Expanded sections of the $^{13}C\{^{1}H\}$ NMR spectrum (75.47 MHz) of **3-Sn** in THF-d_8 at 298 K shown in *Figure S32*; the ^{13}C signal of the deuterated solvent is marked with the character S.

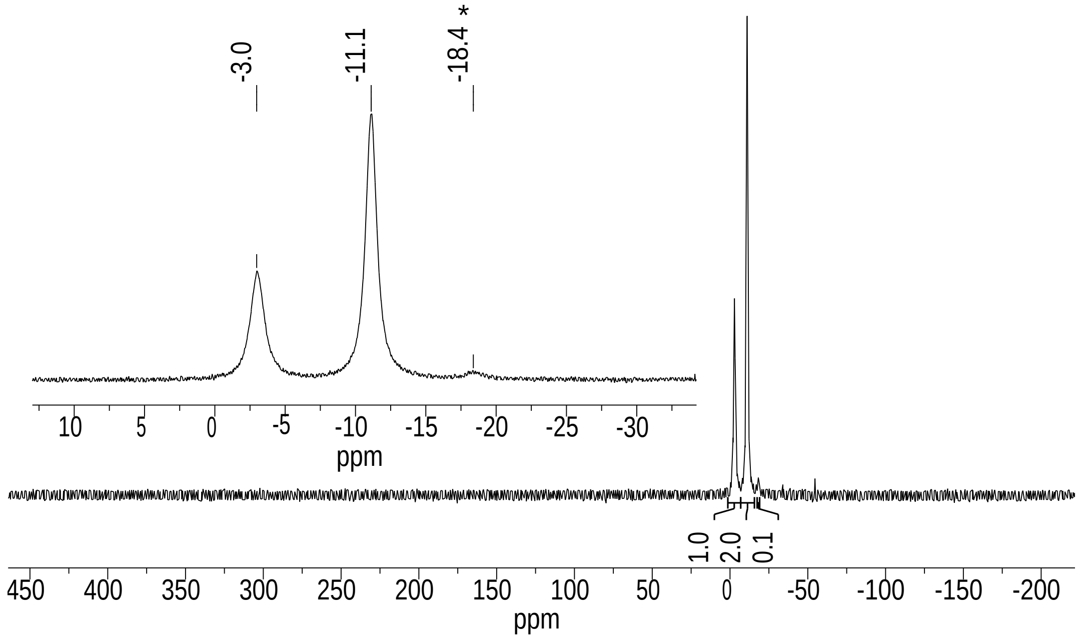

Figure S34. $^{31}P\{^{1}H\}$ NMR spectrum (121.5 MHz) of compound **3-Sn** in THF-d_8 at 298 K; the signal marked with an asterisk in the enlarged excerpt of the spectrum originates from the metallostannylene [(κ^3-tmps)(CO)$_3$Nb–SnAr^{Mes}] (**4-Sn**).

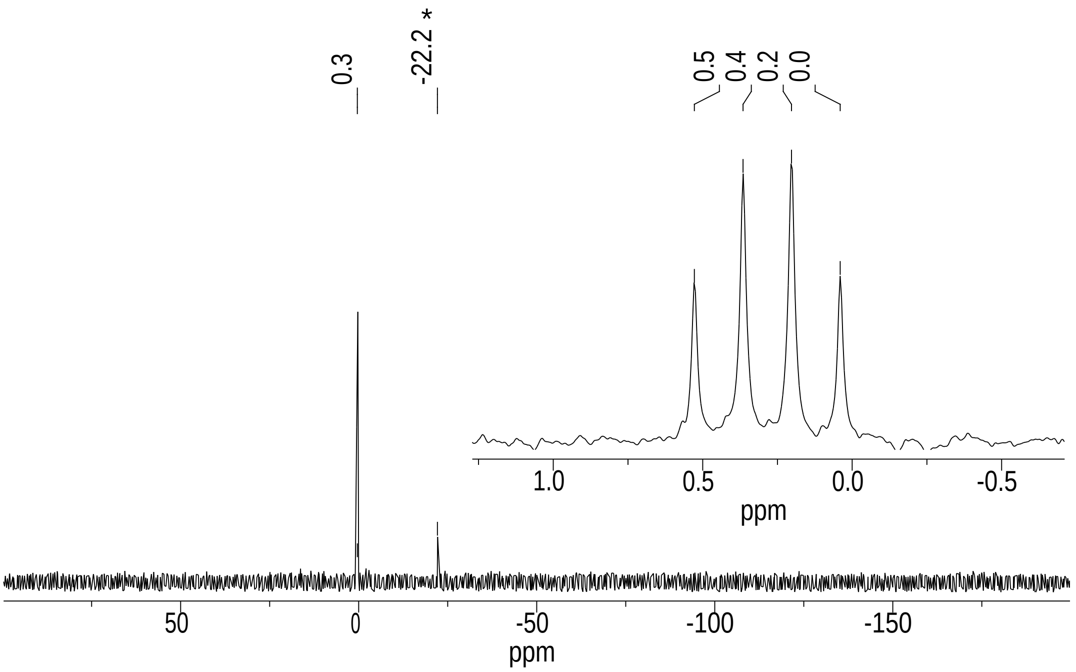

Figure S35. $^{29}Si\{^1H\}$ NMR spectrum (59.63 MHz) of **3-Sn** in THF-d_8 at 298 K; the signal marked with an asterisk (*) originates from oligomeric siloxanes coming from the polymer syringe used for the sample preparation. An enlarged excerpt of the spectrum with the signal at δ = 0.3 ppm is shown in the inset.

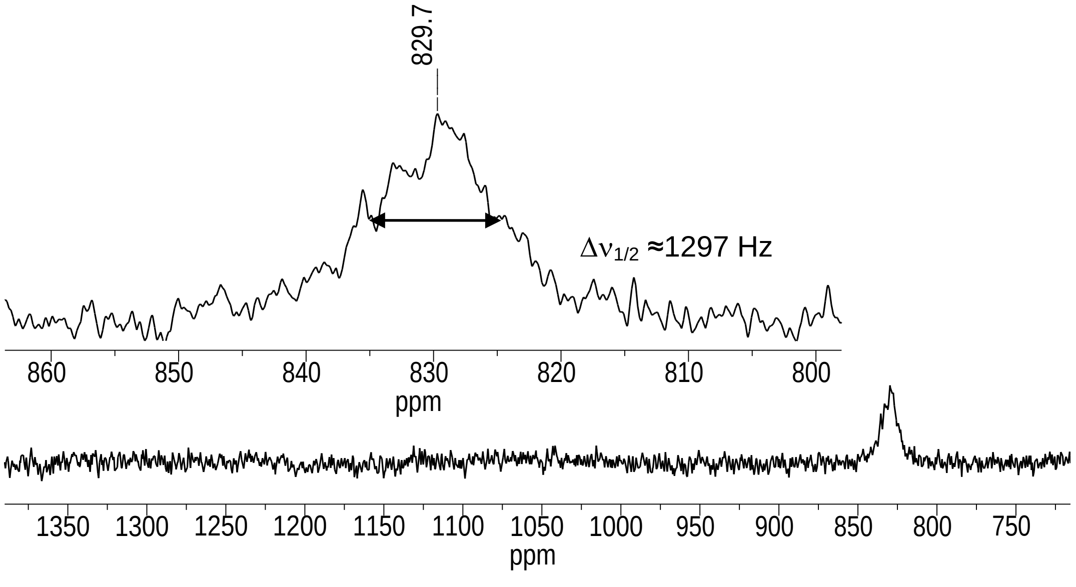

Figure S36. $^{119}Sn\{^1H\}$ NMR spectrum (111.9 MHz) of **3-Sn** in THF-d_8 at 243 K. The inset shows an enlarged excerpt of the spectrum.

2.5 Reaction of 3-Ge with water

0.44 mL of a solution of water in THF (c = 0.28 mmol mL^{-1}, 0.12 mmol, 1.0 equiv.) were added to a deep magenta solution of 100 mg (0.12 mmol) of **3-Ge** in 4 mL of THF at 0 °C. The solution was stirred at room temperature for 1 h. No color change was observed and IR monitoring of the reaction did not reveal a conversion of **3-Ge**. The solution was then heated to 60 °C for 2 h. Again, no color change and no conversion of the germylidyne complex occurred according to IR spectroscopy. At this point, 2 mL of water were added (ca. 111 mmol, 925 equiv.), and the deep magenta solution was heated at 60 °C for 3 h, upon which the color of the solution changed from deep magenta to pale orange. All volatiles were removed under reduced pressure, and the brownish residue was dried under vacuum for 3 h. Extraction with benzene (3 × 2 mL), gave a brownish, insoluble residue and a pale orange extract. The extract was evaporated to dryness *in vacuo* to give the germandiol $Ge(Ar^{Mes})H(OH)_2$ as a pale yellow solid, which was dried for 1 h under fine vacuum. Yield: 50 mg (0,12 mmol, 100 %).

IR (THF, cm^{-1}): $\tilde{\nu}$ = 3600 (s), 3398 (s, br) (ν_{OH}), 2104 (m) (ν_{GeH}).

^{1}H NMR (300.1 MHz, benzene-d_6, 298 K, ppm, *Figure S37*): δ = 0.91 (d, ^{3}J(H,H) = 3.5 Hz, 2H, GeH(O*H*)$_2$), 2.11 (s, 18H, 2 × $C^{2,6}$-*Me* + 2 × C^{4}-*Me*, Mes), 5.61 (t, ^{3}J(H,H) = 3.5 Hz, 1H, Ge*H*(OH)$_2$), 6.79 (s, 4H, 2 × $C^{3,5}$-*H*, Mes), 6.89 (d, ^{3}J(H,H) = 7.5 Hz, $C^{3,5}$-*H*, C_6H_3), 7.21 (t, ^{3}J(H,H) = 7.5 Hz, 1H, C^{4}-*H*, C_6H_3).

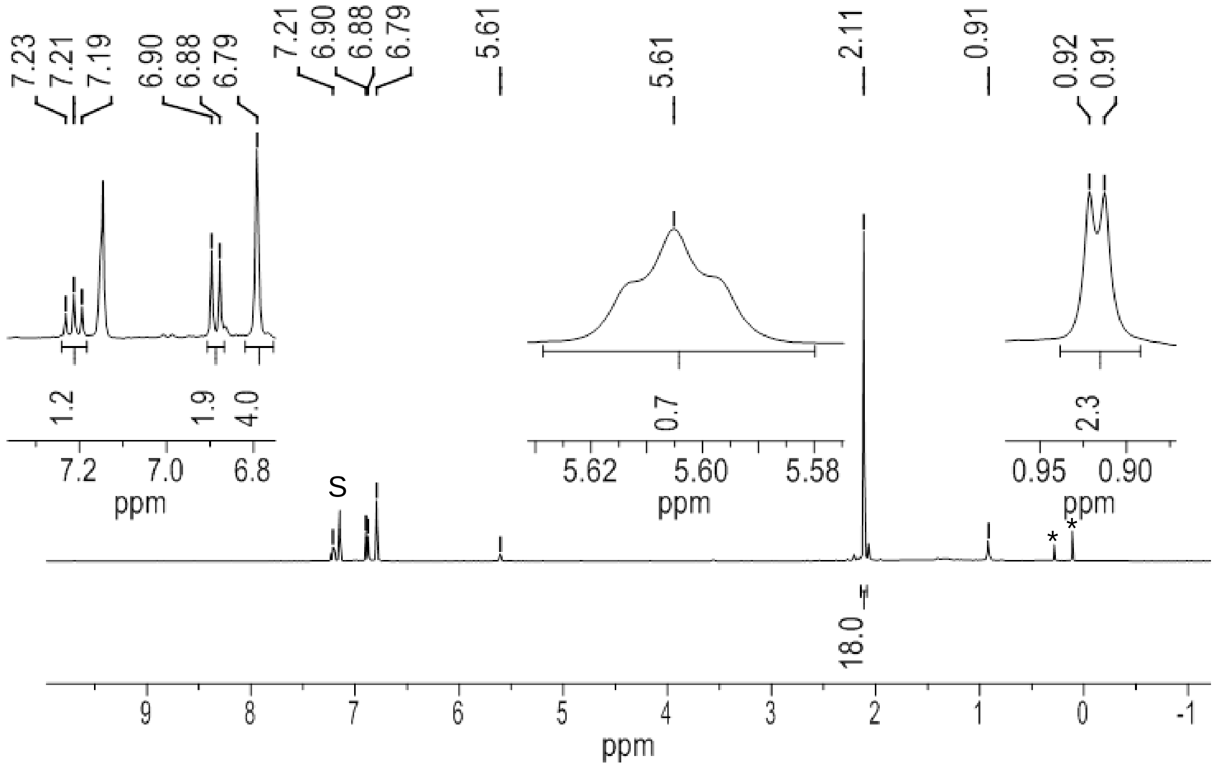

Figure S37. ^{1}H NMR spectrum (300.1 MHz) of $Ge(Ar^{Mes})H(OH)_2$ in benzene-d_6 at 298 K; the character S marks the residual proton signal of the deuterated solvent; the signals marked with an asterisk (*) originate from tiny

impurities present in the recovered deuterated solvent, which was used for recording this ^{1}H NMR spectrum. Enlarged excerpts of the spectrum are shown in the insets.

2.6 [Cp(CO)$_3$Nb=Ge(Ar^{Mes})Cl)] (5-Ge)

A Schlenk tube was charged with 100 mg (0.37 mmol) of [CpNb(CO)$_4$], 156 mg (0.37 mmol, 1 eq.) of Ge(Ar^{Mes})Cl and 15 mL of THF, and attached to a mercury bubbler. The orange solution was then irridiated with a blue light LED (λ = 465 nm, 2.5 W), which was placed in direct contact with the reaction vessel. The color of the reaction mixture changed to red-brown and gas-evolution was observed. Complete consumption of the starting material and a quite selective formation of **5-Ge** after 4h of irradiation was confirmed by IR spectroscopy. The solvent was removed under reduced pressure and the resulting brown residue was washed with *n*-pentane (4 × 5 mL), and then extracted with boiling *n*-hexane (4 × 10 mL). Upon cooling the extract to room temperature, red-orange, spectroscopically pure crystals of **5-Ge** separated from the solution, which were filtered from the orange mother liquor and dried *in vacuo* for 1 h. Yield: 62 mg (0.09 mmol, 25 %).

Compound **5-Ge** is almost insoluble in *n*-pentane at room temperature, moderately soluble in toluene and boiling *n*-hexane, and readily soluble in THF.

IR (*n*-hexane, cm^{-1}): $\tilde{\nu}$ = 1984 (vs), 1923 (s), 1905 (vs) (ν_{CO}).

IR (THF, cm^{-1}, *Figure S38*): $\tilde{\nu}$ = 1980 (vs), 1910 (s, sh), 1899 (vs) (ν_{CO}).

^{1}H NMR (300.1 MHz, benzene-d_6, 298 K, ppm): δ = 2.10 (s, 6H, 2 × C^{4}-*Me*, Mes), 2.28 (s, 12H, 2 × C^{2,6}-*Me*, Mes), 4.80 (s, 5H, C_5H_5), 6.84 (s, 4H, 2 × C^{3,5}-*H*, Mes), 6.95 (d, ^{3}J(H,H) = 7.5 Hz, C^{3,5}-*H*, C_6H_3), 7.19 (t, ^{3}J(H,H) = 7.5 Hz, 1H, C^{4}-*H*, C_6H_3).

$^{13}C\{^{1}H\}$ NMR (75.47 MHz, benzene-d_6, 298 K, ppm, *Figure S39*): δ = 21.1 (s, 2C, 2 × C^{4}-*Me*, Mes), 21.8 (br, $\Delta\nu_{1/2}$ ≈ 7 Hz, 4C, 2 × C^{2,6}-*Me*, Mes), 93.5 (s, 5C, C_5H_5), 128.9 (2C, $C^{3,5}$-H, C_6H_3), 129.0 (br, $\Delta\nu_{1/2}$ ≈ 60 Hz, 4C, 2 × $C^{3,5}$-H, Mes), 130.9 (s, 1C, C^{4}-H, C_6H_3), 136.0 (s, 2C, 2 × C^{1}, Mes), 137.8 (br, $\Delta\nu_{1/2}$ ≈ 26 Hz, 4C, 2 × $C^{2,6}$, Mes), 138.3 (s, 2C, 2 × C^{4}, Mes), 144.1 (s, 2C, $C^{2,6}$, C_6H_3), 158.7 (s, 1C, C^{1}, C_6H_3).[S13]

[S13] No two-dimensional $^{1}H,^{13}C$ correlated NMR spectra of **5-Ge** using the HSCQ and HMBC method were recorded. Therefore, assignment of the ^{13}C NMR signals of **5-Ge** is tentative and was based a) on the broadness of some signals resulting from incipient hindrance to rotation of the Ar^{Mes} substituent about the Ge-C$_{aryl}$ bond and b) on comparison of the relative position and intensity of the signals with those of the molybdenum compounds [Cp(CO)$_2$(H)Mo=Ge(Ar^{Mes})X)] (X = OH, OMe, Cl), for which an unambiguous assignment of all ^{13}C NMR signals had been carried out using $^{1}H,^{13}C$ correlation spectroscopy (see K. W. Stumpf, *Germylidene and Germylidyne Complexes of Molybdenum*, Dissertation (ISBN 978-3-8439-1849-7), University of Bonn, Verlag Dr. Hut, **2014**). The ^{13}C NMR signals of the carbonyl ligands could not be detected after 5120 scans at a concentration of 0.06 mmol mL^{-1}.

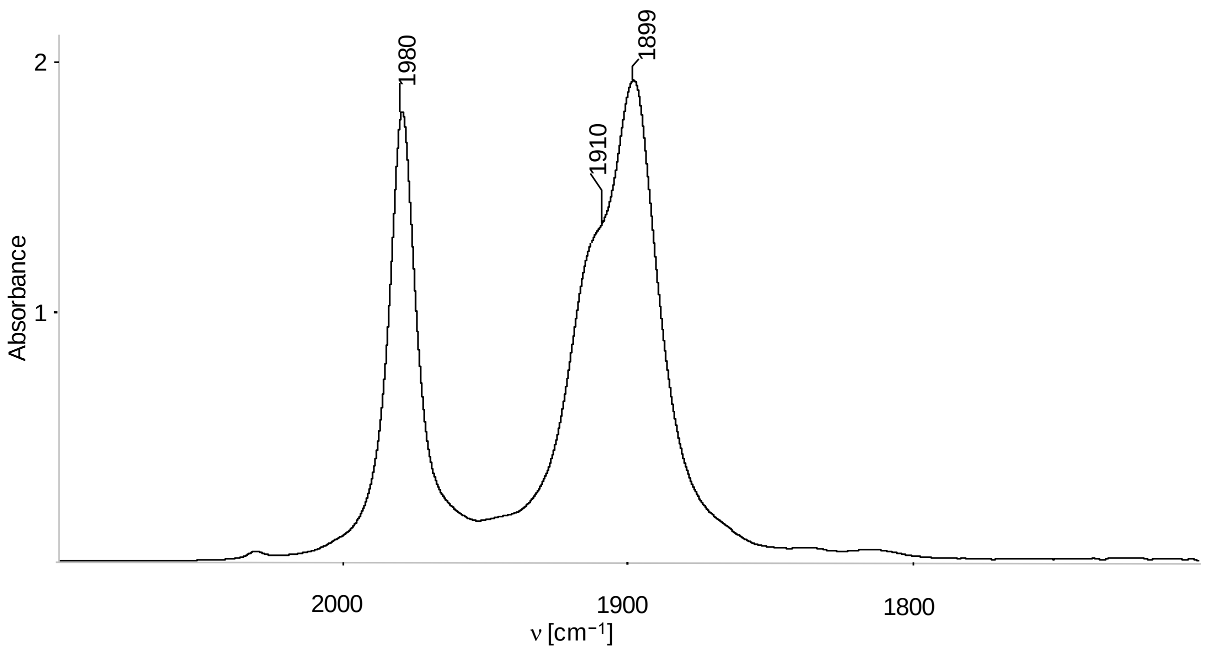

Figure S38. FT-IR spectrum of **5-Ge** in THF in the range of 2100 – 1700 cm^{-1}.

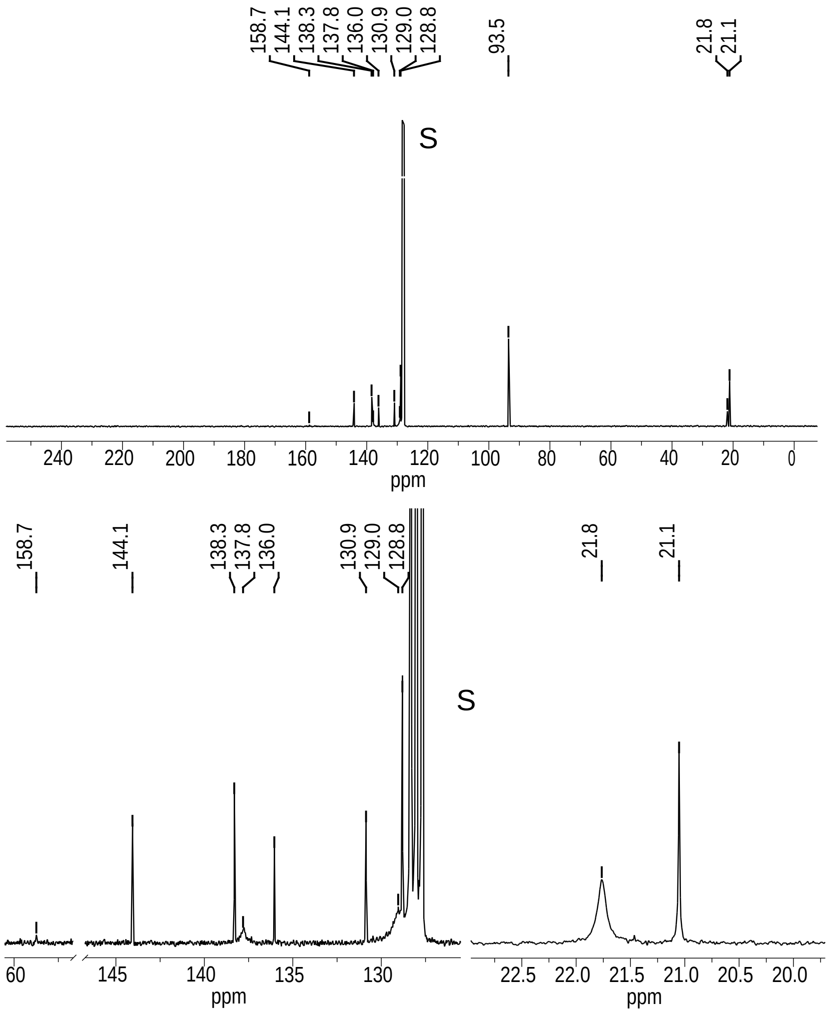

Figure S39. $^{13}C\{^{1}H\}$ NMR spectrum (75.47 MHz) of **5-Ge** in benzene-d_6 at 298 K; the character S marks the ^{13}C signals of the deuterated solvent; enlarged excerpts of the spectrum are shown in the insets.

3 Cyclic Voltammetry

The cyclic voltammetric studies of the ylidyne complexes **2-Si**, **3-Ge** and **3-Sn** were performed with an Autolab Eco electrochemical workstation composed of an Autolab PGSTAT 20 potentiostat/galvanostat. The results were processed with the Autolab software version 4.9. All experiments were carried out under argon atmosphere in a gas-tight specially designed full-glass-three-electrode cell at −11 °C. A glass-carbon disk electrode with a diameter of 2 mm was used as working electrode and a platinum wire with a diameter of 1 mm as counter electrode. A solution of decamethylferrocene (dmfc, 4 mM)[S14] and $[N(^{n}Bu)_4][PF_6]$ (0.1 M) in THF with a platinum wire was used as reference electrode, which was separated from the substrate/electrolyte solution via a Vycor frit of 4 mm thickness.

All experiments were carried out in THF solution containing $[N(^{n}Bu)_4][PF_6]$ as supporting electrolyte in a concentration of 0.1 M. The solvent (THF) was dried upon refluxing over sodium-wire, purged during reflux with argon and distilled under argon. The electrolyte ($[N(^{n}Bu)_4][PF_6]$) was recrystallized twice from ethanol and dried for 24 h at 80 °C using a drying pistol. *iR*-drop compensation was applied for all experiments.

All potentials are given versus the reference electrode mentioned above. For comparison, the half-wave potential of the $[FeCp_2]^+/FeCp_2$ ($fc^{1+/0}$) redox couple was determined versus the reference electrode by separate cyclic voltammetric experiments under the same conditions and found to be $E_{½}$ = +440 mV.

The cyclic voltammogram of **2-Si** displays four irreversible electron transfer processes (*Figure S40* and *Table S1*). The two irreversible processes at very low potentials ($E^{pc}(1)$ = −2110 mV and ($E_{pc}(2)$ = −2332 mV; scan rate (v) = 100 mVs^{-1}) involve consecutive one-electron reductions of the silylidyne complex, and those at $E_{pa}(3)$ = −433 mV and $E_{pa}(4)$ = +55 mV (v = 100 mVs^{-1}) two consecutive, irreversible one-electron oxidations.

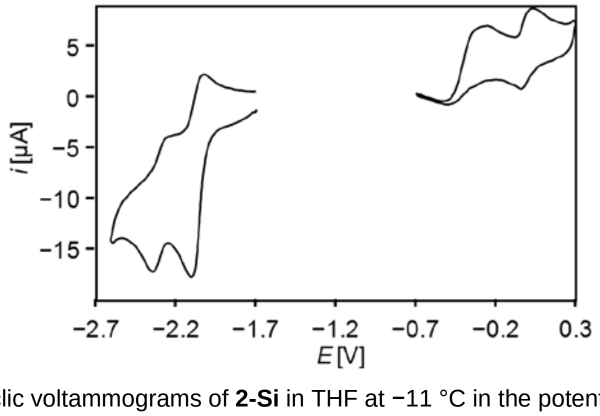

Figure S40. Single scan cyclic voltammograms of **2-Si** in THF at −11 °C in the potential range of −2.7 to +0.3 V at a scan rate of 100 mVs^{-1}; reference electrode: 0.4 mM $dmfc^{+/0}$ / 0.1 M $[N(^{n}Bu)_4][PF_6]$ / THF solution.

[S14] The $dmfc^{1+/0}$ redox couple has been shown to be a superior reference standard for redox potentials compared to the $fc^{1+/0}$ redox couple, see: I. Noviandri, K. N. Brown, D. S. Fleming, P. T. Gulyas, P. A. Lay, A. F. Masters, L. Phillips, *J. Phys. Chem. B* **1999**, *103*, 6713.

Table S1. Selected results of the cyclic voltammetric studies of **2-Si** in THF.[a]

v [mV s^{-1}]	E_{pa}[mV]	E_{pc}[mV]	ΔE_p [mV]	i_{pc}/i_{pa}	$(E_{pa} + E_{pc})/2$ [mV]
100	−2264	−2332	68	1.23	−2298
100	−2038	−2110	72	1.40	−2074
100	−433	−503	70	0.43	−468
100	+55	−5	60	0.77	+25

[a] v: scan rate; ΔE_p: peak potential separation; $\Delta E_p = E_{pa} - E_{pc}$, where E_{pa} is the anodic peak potential and E_{pc} the cathodic peak potential; i_{pc} / i_{pa}: ratio of cathodic and anodic peak current; $E_{½}$: half wave potential. All potentials are given versus the 0.4 mM $dmfc^{1+/0}$ / 0.1 M $[N(^nBu)_4][PF_6]$ / THF reference electrode.

The cyclic voltammograms of the germylidyne complex **3-Ge** display three electron-transfer steps (*Figure S41*). The two steps at $E_{1/2}$ (1) = −2612 mV and $E_{1/2}$ (2) = −405 mV involve a reversible one-electron reduction and oxidation of the germylidyne complex **3-Ge**, respectively, whereas the third step at $E_{pa}(3)$ = + 646 mV (at v = 100 mVs^{-1}) involves an irreversible follow-up oxidation of **3-Ge**$^+$ (*Table 2*). The following criteria were applied to verify the reversibility of the first two electron transfer steps. Firstly, the half-wave potential $E_{1/2}$ was found to be constant and the peak current ratio i_{pc} / i_{pa} = 1 for all scan rates (*Table 2*). Secondly the peak potential separation ΔE_p did not change with increasing scan rate and ranged from 60 mV – 64 mV (*Table 2*). ΔE_p had a slightly higher value than that expected for an ideal one-electron Nernstian process (52 mV at 262 K), but a similar value to that found for decamethylferrocene under the same conditions (the slightly larger ΔE_p values can be attributed to incomplete *iR* compensation). Thirdly a plot of the cathodic peak current i_{pc} against the square root of the scan rate confirmed an almost linear relationship. Exemplarily, the single scan cyclic voltammograms for the reversible one-electron oxidation of **3-Ge** at $E_{1/2}$ = −405 mV are displayed in *Figure S42*, and a plot of the cathodic peak current i_{pc} against the square root of the scan rate for this electron-transfer step is depicted in *Figure S43*. The redox step at $E_{1/2}$ = −405 mV involves a one-electron oxidation of **3-Ge** as confirmed by chemical means. Thus, **3-Ge** did not react with cobaltocene ($E_{1/2}$ in DME = −740 mV) vs. the $dmfc^{+/0}$ redox couple),[S15] whereas a rapid oxidation was observed upon treatment of **3-Ge** with one equivalent of $[Fe(\eta^5\text{-}C_5Me_5)_2][B(Ar^F)_4]$ ($Ar^F = C_6H_3\text{-}3,5\text{-}(CF_3)_2$).

[S15] J. R. Aranzaes, M.-C. Daniel, D. Astruc, *Can. J. Chem.* **2006**, *84*, 288.

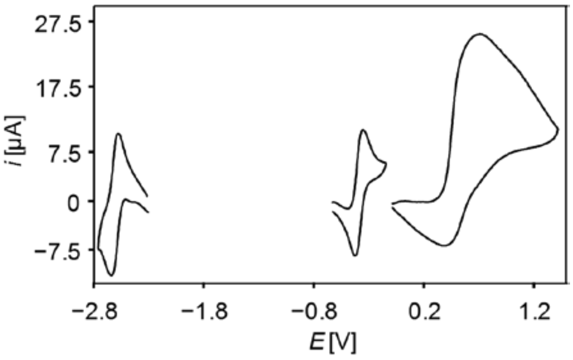

Figure S41. Single scan cyclic voltammograms of **3-Ge** in THF at −11 °C in the potential range of −2.8 to +1.5 V at a scan rate of 100 mVs^{-1}; reference electrode: 0.4 mM $dmfc^{1+/0}$ / 0.1 M $[N(^nBu)_4][PF_6]$ / THF solution.

Table 2. Selected results of the cyclic voltammetric studies of **3-Ge** in THF. [a]

v [mV s^{-1}]	E_{pa}[mV]	E_{pk}[mV]	ΔE_p [mV]	i_{pc}/i_{pa}	$E_{½} = (E_{pa} + E_{pc})/2$ [mV]
100	−2580	−2644	64	0.95	−2612
400	−2583	−2643	60	0.99	−2613
600	−2580	−2644	64	1.00	−2612
800	−2580	−2642	62	0.99	−2611
1000	−2580	−2640	60	1.00	−2610
100	−373	−435	62	0.99	−404
200	−373	−433	60	1.03	−403
400	−375	−435	60	1.04	−405
600	−374	−436	62	1.01	−405
800	−375	−435	60	1.04	−405
1000	−373	−437	64	1.05	−405
100	+646	+434	212	0.31	-

[a] v: scan rate; ΔE_p: peak potential separation; $\Delta E_p = E_{pa} – E_{pc}$, where E_{pa} is the anodic peak potential and E_{pc} the cathodic peak potential; i_{pc} / i_{pa}: ratio of cathodic and anodic peak current; $E_{½}$: half wave potential. All potentials are given versus the 0.4 mM $dmfc^{1+/0}$ / 0.1 M $[N(^nBu)_4][PF_6]$ / THF reference electrode.

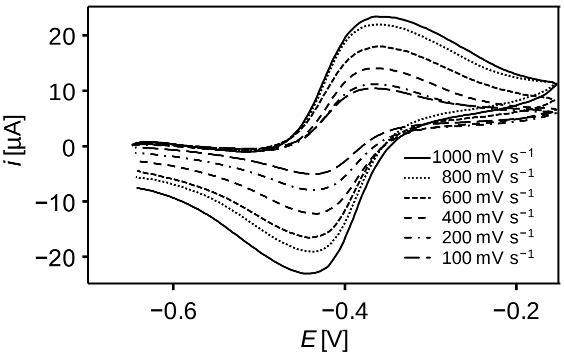

Figure S42. Single scan cyclic voltammograms of **3-Ge** in THF at −11 °C in the potential range of −0.70 to −0.15 V at different scan rates; reference electrode: 0.4 mM dmfc$^{+/0}$ / 0.1 M [N(^{n}Bu)$_4$][PF$_6$] / THF solution.

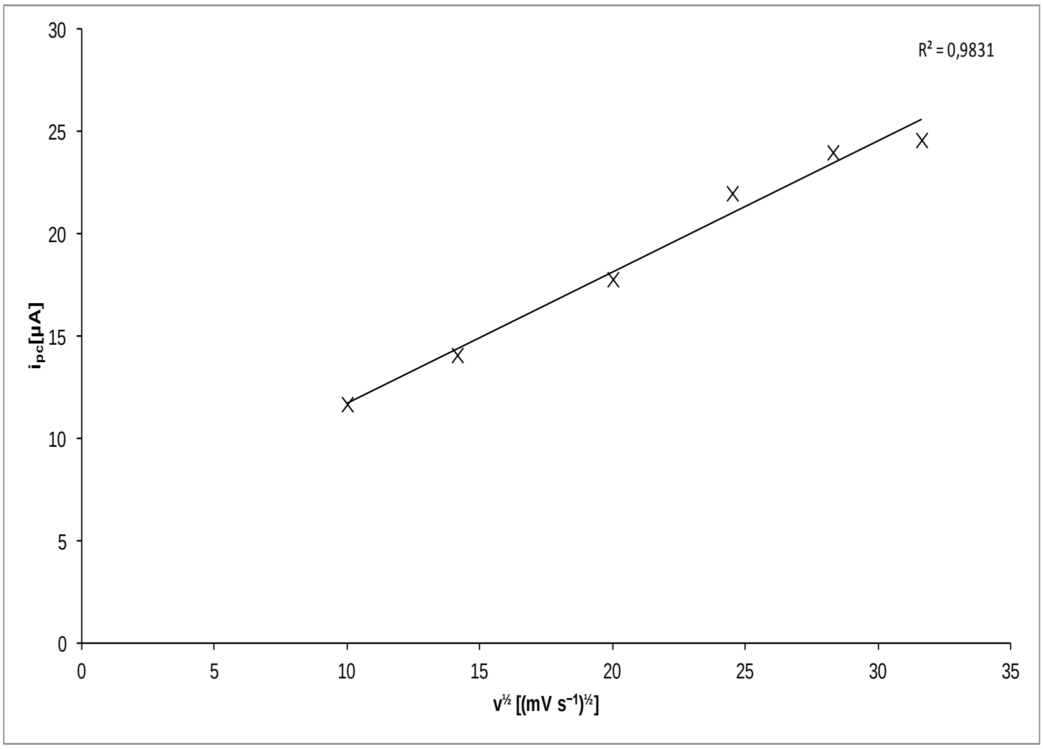

Figure S43. Plot of the cathodic peak current i_{pc} against the square root of the scan rate $v^{½}$ for the reversible one-electron oxidation of **3-Ge** in THF at $E_{1/2}$ = −405 mV.

The cyclic voltammograms of the stannylidyne complex **3-Sn** display as those of **3-Ge** three electron transfer steps (*Figure S44*). However, in contrast to **3-Ge** all redox steps are irreversible (*Table 3*).

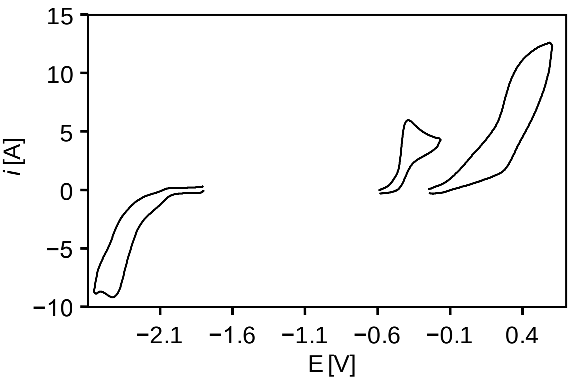

Figure S44. Single scan cyclic voltammograms of **3-Sn** in THF at −11 °C in the potential range of −2.60 to +0.65 V at a scan rate of 100 mVs^{-1}; reference electrode: 0.4 mM $dmfc^{+1/0}$ / 0.1 M $[N(^{n}Bu)_4][PF_6]$ / THF solution.

Table 3. Selected results of the cyclic voltammetric studies of **3-Sn** in THF.[a]

v [mV s^{-1}]	E_{pa}[mV]	E_{pk}[mV]	ΔE_p [mV]	i_{pc}/i_{pa}	$(E_{pa} + E_{pc})/2$ [mV]
100	−2322	−2432	110	1.83	−2377
100	−406	−464	58	0.41	−435
100	+503	+433	70	-	+468

[a] v: scan rate; ΔE_p: peak potential separation; $\Delta E_p = E_{pa} - E_{pc}$, where E_{pa} is the anodic peak potential and E_{pc} the cathodic peak potential; i_{pc} / i_{pa}: ratio of cathodic and anodic peak current; $E_{½}$: half wave potential. All potentials are given versus the 0.4 mM $dmfc^{1+/0}$/ 0.1 M $[N(^{n}Bu)_4][PF_6]$ / THF reference electrode.

4 Crystal structure determination

Red-brown, block-like crystals of **2-Si** suitable for X-ray diffraction analysis were obtained by slow evaporation of a benzene solution of the compound in the glovebox for one week. Plate-like, dark red crystals of **3-Ge**(THF) were grown by slow diffusion of *n*-hexane into a concentrated THF solution at −25 °C. Dark brown, plate like crystals of **3-Sn**(toluene) were formed upon slow diffusion of *n*-hexane into a concentrated toluene solution of the stannylidyne complex at −25 °C. Red-orange, rod-like cyrstals of **5-Ge** were obtained upon slow cooling of a saturated boiling *n*-hexane solution to room temperature.

The data collections of **2-Si**, **3-Sn**(C_7H_8) and **5-Ge** were performed on a Bruker X8-KappaApexII diffractometer, and the data collection of **3-Ge**(C_4H_8O) on a Nonius KappaCCD diffractometer, using graphite monochromated Mo-Kα radiation (λ = 0.71073 Å). The diffractometers were equipped with a low–temperature device (Bruker Kryoflex, 100 K; Oxford Cryostream 600 Series, 123 K). Intensities were measured by fine-slicing φ- and ω-scans and corrected for background, polarization and Lorentz effects. A numerical absorption correction was applied for the data sets. The structures were solved by direct methods and refined anisotropically by the least-squares procedure implemented in the ShelX program system.[S16] The hydrogen atoms were included isotropically using the riding model on the bound carbon atoms.

CCDC numbers CCDC 1553387 (**2-Si**), CCDC 1553388 (**3-Ge**), CCDC 1553389 (**3-Sn**) and 1555671 (**5-Ge**) contain the supplementary crystallographic data for this paper, which can be obtained free of charge from the Cambridge Crystallographic Data Centre via www.ccdc.cam.ac.uk/data_request/cif.

[S16] Sheldrick, G. M. ShelXS97 and ShelXL97, University of Göttingen, Germany, **1997**.

4.1 Crystal data and structure refinement for 2-Si

Empirical formula	$C_{36}H_{76}NbO_2P_3Si_6$
Formula weight	895.32
Temperature/K	100
Crystal system	monoclinic
Space group	$P2_1/c$
a/Å	13.548(5)
b/Å	18.581(9)
c/Å	20.379(7)
α/°	90
β/°	100.01(2)
γ/°	90
Volume/Å^3	5052(4)
Z	4
ρ_{calc} g/cm^3	1.177
μ/mm^{-1}	0.502
F(000)	1912.0
Crystal size/mm^3	0.12 × 0.06 × 0.03
Absorption correction	empirical
Tmin; Tmax	0.537366; 0.745830
Radiation	MoKα (λ = 0.71073)
2Θ range for data collection/°	4.012 to 55.998°
Completeness to theta	0.991
Index ranges	-17 ≤ h ≤ 17, 0 ≤ k ≤ 24, 0 ≤ l ≤ 26
Reflections collected	37575
Independent reflections	12043 [R_{int} = 0.1653, R_{sigma} = 0.4568]
Data/restraints/parameters	12043/0/455
Goodness-of-fit on F^2	0.826
Final R indexes [I>=2σ (I)]	R_1 = 0.0815, wR_2 = 0.1403
Final R indexes [all data]	R_1 = 0.2870, wR_2 = 0.1710
Largest diff. peak/hole / e Å^{-3}	1.09/-1.62

4.2 Crystal data and structure refinement for 3-Ge(THF)

Empirical formula	$C_{40}H_{60}GeNbO_3P_3Si$
Formula weight	875.38
Temperature/K	123.15
Crystal system	monoclinic
Space group	$P2_1$
a/Å	8.4180(3)
b/Å	22.9239(7)
c/Å	11.8653(5)
α/°	90.00
β/°	107.5480(16)
γ/°	90.00
Volume/Å^3	2183.14(14)
Z	2
ρ_{calc} g/cm^3	1.332
μ/mm^{-1}	1.122
F(000)	912.0
Crystal size/mm^3	0.4 × 0.3 × 0.1
Radiation	MoKα (λ = 0.71073)
2Θ range for data collection/°	5.06 to 50.5
Index ranges	-10 ≤ h ≤ 8, -27 ≤ k ≤ 25, -14 ≤ l ≤ 13
Reflections collected	8273
Independent reflections	6030 [R_{int} = 0.0427, R_{sigma} = 0.0526]
Data/restraints/parameters	6030/1/455
Goodness-of-fit on F^2	1.027
Final R indexes [I>=2σ (I)]	R_1 = 0.0283, wR_2 = 0.0666
Final R indexes [all data]	R_1 = 0.0305, wR_2 = 0.0675
Largest diff. peak/hole / e Å^{-3}	0.38/-0.77
Flack parameter	-0.010(7)

4.3 Crystal data and structure refinement for 3-Sn(toluene)

Empirical formula	$C_{43}H_{60}NbO_2P_3SiSn$
Formula weight	941.51
Temperature/K	100
Crystal system	monoclinic
Space group	$P2_1$
a/Å	8.5148(6)
b/Å	23.2579(16)
c/Å	12.0766(8)
α/°	90
β/°	108.779(2)
γ/°	90
Volume/Å^3	2264.3(3)
Z	2
ρ_{calc} g/cm^3	1.381
μ/mm^{-1}	0.970
F(000)	968.0
Crystal size/mm^3	0.12 × 0.09 × 0.04
Absorption correction	empirical
Tmin; Tmax	0.5373; 0.7460
Radiation	MoKα (λ = 0.71073)
2Θ range for data collection/°	5.45 to 55.998°
Completeness to theta	0.995
Index ranges	-11 ≤ h ≤ 10, -30 ≤ k ≤ 30, -15 ≤ l ≤ 15
Reflections collected	20515
Independent reflections	10697 [R_{int} = 0.0408, R_{sigma} = 0.0641]
Data/restraints/parameters	10697/25/475
Goodness-of-fit on F^2	1.233
Final R indexes [I>=2σ (I)]	R_1 = 0.0703, wR_2 = 0.1707
Final R indexes [all data]	R_1 = 0.0782, wR_2 = 0.1737
Largest diff. peak/hole / e Å^{-3}	3.40/-2.85
Flack parameter	0.17(5)

4.4 Crystal data and structure refinement for 5-Ge

Empirical formula	$C_{32}H_{30}ClGeNbO_3$
Formula weight	663.51
Temperature/K	100
Crystal system	orthorombic
Space group	Pnma
a/Å	20.237(3)
b/Å	15.315(2)
c/Å	9.180(1)
α/°	90
β/°	90
γ/°	90
Volume/Å^3	2845.2(7)
Z	4
ρ_{calc} g/cm^3	1.549
μ/mm^{-1}	1.585
F(000)	1344
Crystal size/mm^3	0.10 × 0.03 × 0.02
Absorption correction	empirical
Tmin; Tmax	0.9690; 0.8576
Radiation	MoKα (λ = 0.71073)
2Θ range for data collection/°	2.44 to 28.00
Completeness to theta	0.997
Index ranges	-26 ≤ h ≤ 26, -20 ≤ k ≤ 19, -9 ≤ l ≤ 12
Reflections collected	11778
Independent reflections	3556 [R_{int} = 0.0589]
Data/restraints/parameters	3556/0/187
Goodness-of-fit on F^2	1.066
Final R indexes [I>=2σ (I)]	R_1 = 0.0384, wR_2 = 0.0834
Final R indexes [all data]	R_1 = 0.0599, wR_2 = 0.0907
Largest diff. peak/hole / e Å^{-3}	0.823/-0.779
